# Supplementary material for: Accessibility and quality of drug company disclosures of payments to healthcare professionals and organisations in 37 countries: a European policy review
Source: BMJ Open. 2021 Dec 16;11(12):e053138. doi: 10.1136/bmjopen-2021-053138 (PMC8679071; doi:10.1136/bmjopen-2021-053138)
Supplement: Supplementary data [file bmjopen-2021-053138supp002.pdf]

Online Supplement 2. Responses to stakeholder survey

Part 1. Pharmaceutical industry trade groups

Country	Name of pharmaceutical industry trade group	Reply received	Nature of reply
AUSTRIA	Fachverband der Chemischen Industrie Österreichs	Yes	Answered all questions
BOSNIA AND HERZEGOVINA	Association of Research-based Medicine Producers in Bosnia & Herzegovina	No	N/A
BULGARIA	Association of the Research-based Pharmaceutical Manufacturers in Bulgaria	No	N/A
CROATIA	iF! – Inovativna farmaceutska inicijativa	No	N/A
CYPRUS	The Cyprus Association of Research and Development Pharmaceutical Companies	No	N/A
CZECH REPUBLIC	Asociace inovativního farmaceutického průmyslu	Yes	Answered some questions
DENMARK	LægemiddelindustriforeningenLersø	Yes	Answered some questions
ESTONIA	The Association of Pharmaceutical Manufacturers in Estonia	No	N/A
FINLAND	Lääketeollisuus	Yes	Answered all questions
GERMANY	Freiwillige Selbstkontrolle für die Arzneimittelindustrie	No	N/A
GREECE	Hellenic Association of Pharmaceutical Companies	No	N/A
HUNGARY	Association of Innovative Pharmaceutical Manufacturers	No	N/A
ICELAND	Icelandic Association of the Pharmaceutical Industry	Yes	Holding message
IRELAND	Irish Pharmaceutical Healthcare Association	Yes	Answered all questions
ITALY	Associazione delle imprese del farmaco	No	N/A
LATVIA	Association of International Innovative Pharmaceuticals Producers	Yes	Answered some questions
LITHUANIA	Innovative Pharmaceutical Industry Association	Yes	Answered all questions
LUXEMBOURG	Association pharmaceutique luxembourgeoise	Yes	Answered all questions
NORTH MACEDONIA	Association of Foreign Innovative Manufacturers in Macedonia	Yes	Answered all questions
NORWAY	Legemiddelindustrien	Yes	Answered all questions
POLAND	Związek Pracodawców Innowacyjnych Firm Farmaceutycznych	Yes	Holding message

PORTUGAL	Associação Portuguesa da Indústria Farmacêutica	No	N/A
ROMANIA	Association of International Medicines Manufacturers	No	N/A
RUSSIA	Association of International Pharmaceutical Manufacturers	No	N/A
SERBIA	Innovative Drug Manufacturers' Association	No	N/A
SLOVAKIA	Association of the Innovative Pharmaceutical Industry	No	N/A
SLOVENIA	Forum of International Research and Development Pharmaceutical Companies, EIG	No	N/A
SPAIN	Asociación Nacional Empresarial de la Industria Farmacéutica	Yes	Answered some questions
SWEDEN	Läkemedelsindustriföreningen	Yes	Answered all questions
SWITZERLAND	Science Industries Switzerland	Yes	Holding message
TURKEY	Araştırmacı İlaç Firmaları Derneği	Yes	Answered all questions
UK	Association of the British Pharmaceutical Industry	Yes	Answered all questions
UKRAINE	Association of Pharmaceutical Research and Development	No	N/A
EUROPE	EFPIA	No	N/A

Part 2. Public and multistakeholder bodies overseeing payment disclosure

Country	Name of public or multistakeholder body overseeing payment disclosure	Nature of authority overseeing payment disclosure	Reply received	Nature of reply
BELGIUM	betransparent.be	Multistakeholder body	No	N/A
DENMARK	Danish Medicines Agency	Public body	Yes	Answered some questions
ESTONIA	State Agency of Medicines	Public body	No	N/A
FRANCE	Ministry of Health	Public body	Yes	Holding message
GREECE	National Organisation for Medicines	Public body	No	N/A
HUNGARY	National Institute of Pharmacy and Nutrition	Public body	Yes	Answered all questions
LATVIA	Latvian Health Inspectorate	Public body	Yes	Answered all questions
LITHUANIA	Lithuanian State Medicines Control Agency	Public body	Yes	Answered all questions
THE NETHERLANDS	Vereniging Innovatieve Geneesmiddelen	Multistakeholder body	Yes	Inquiry redirected
PORTUGAL	INFARMED - National Authority of Medicines and Health Products	Public body	Yes	Answered some questions
ROMANIA	National Agency for Medicines and Medical Devices in Romania	Public body	Yes	Holding message
SLOVAKIA	National Health Information Center	Public body	Yes	Answered some questions
TURKEY	Turkish Medicines and Medical Devices Agency	Public body	Yes	Holding message
