# Supplementary material for: Accessibility and quality of drug company disclosures of payments to healthcare professionals and organisations in 37 countries: a European policy review
Source: BMJ Open. 2021 Dec 16;11(12):e053138. doi: 10.1136/bmjopen-2021-053138 (PMC8679071; doi:10.1136/bmjopen-2021-053138)
Supplement: Supplementary data [file bmjopen-2021-053138supp003.pdf]

Online Supplement 3. Examples of deviations of data reporting formats from the EFPIA standard "disclosure template"

Part 1: EFPIA "disclosure template"

ANNEX A (binding)
Standardised disclosure template

ANNEX A - STANDARDISED DISCLOSURE TEMPLATE

Date of publication: ....

	Full Name (Art. 1.01)	**HCPs:** City of Principal Practice **HCOs:** city where registered (Art. 3)	Country of Principal Practice (Schedule 1)	Principal Practice Address (Art. 3)	Unique country identifier *OPTIONAL* (Art. 3)	Donations and Grants to HCOs *(Art. 3.01.1.a)*	Contribution to costs of Events *(Art. 3.01.1.b & 3.01.2.a)*			Fee for service and consultancy *(Art. 3.01.1.c & 3.01.2.c)*			**TOTAL** *OPTIONAL*
							Sponsorship agreements with HCOs / third parties appointed by HCOs to manage an Event	Registration Fees	Travel & Accommodation	Fees	Related expenses agreed in the fee for service or consultancy contract, including travel & accommodation relevant to the contract		
**HCPs**	***INDIVIDUAL NAMED DISCLOSURE - one line per HCP*** *(i.e. all transfers of value during a year for an individual HCP will be summed up: itemization should be available for the individual Recipient or public authorities' consultation only, as appropriate)*												
	Dr A					N/A	N/A	*Yearly amount*	*Yearly amount*	*Yearly amount*	*Yearly amount*		
	Dr B					N/A	N/A	*Yearly amount*	*Yearly amount*	*Yearly amount*	*Yearly amount*		
	etc.					N/A	N/A	*Yearly amount*	*Yearly amount*	*Yearly amount*	*Yearly amount*		
	OTHER, NOT INCLUDED ABOVE - where information cannot be disclosed on an individual basis for legal reasons												
	**Aggregate amount attributable to transfers of value to such Recipients** - *Art. 3.02*					N/A	N/A	*Aggregate HCPs*	*Aggregate HCPs*	*Aggregate HCPs*	*Aggregate HCPs*		*Optional*
	**Number of Recipients in aggregate disclosure** - *Art. 3.02*					N/A	N/A	*number*	*number*	*number*	*number*		*Optional*
	% of the number of Recipients included in the aggreate disclosure in the total number, by category, of Recipients disclosed - *Art. 3.02*					N/A	N/A	%	%	%	%		N/A
**HCOs**	***INDIVIDUAL NAMED DISCLOSURE - one line per HCO*** *(i.e. all transfers of value during a year for an individual HCO will be summed up: itemization should be available for the individual Recipient or public authorities' consultation only, as appropriate)*												
	HCO 1					*Yearly amount*	*Yearly amount*	*Yearly amount*	*Yearly amount*	*Yearly amount*	*Yearly amount*		*Optional*
	HCO 2					*Yearly amount*	*Yearly amount*	*Yearly amount*	*Yearly amount*	*Yearly amount*	*Yearly amount*		*Optional*
	etc.					*Yearly amount*	*Yearly amount*	*Yearly amount*	*Yearly amount*	*Yearly amount*	*Yearly amount*		*Optional*
	OTHER, NOT INCLUDED ABOVE - where information cannot be disclosed on an individual basis for legal reasons												
	**Aggregate amount attributable to transfers of value to such Recipients** - *Art. 3.02*					*Aggregate HCOs*	*Aggregate HCOs*	*Aggregate HCOs*	*Aggregate HCOs*	*Aggregate HCOs*	*Aggregate HCOs*		*Optional*
	**Number of Recipients in aggregate disclosure** - *Art. 3.02*					*number*	*number*	*number*	*number*	*number*	*number*		*Optional*
	% of the number of Recipients included in the aggreate disclosure in the total number, by category, of Recipients disclosed - *Art. 3.02*					%	%	%	%	%	%		N/A

	AGGREGATE DISCLOSURE		
**R&D**	Transfers of Value re Research & Development as defined - *Article 3.04 and Schedule 1*	*TOTAL AMOUNT*	*OPTIONAL*

latest update: 27 June 2019

Note

The screenshot was taken from version the EFPIA Code which was in force the time of writing.[1]

Part 2: Examples of data reporting not following the EFPIA "disclosure template"

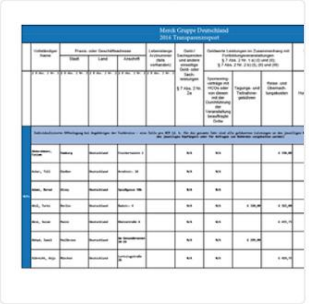

de__merck__2016.pdf
PDF

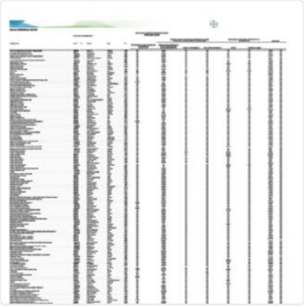

de__bayer__2016.pdf
PDF

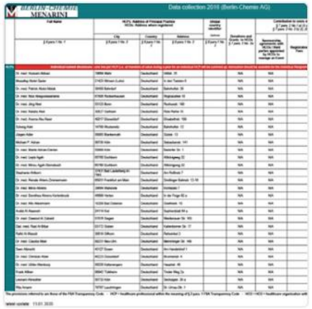

de__berlin-chemie__2016.pdf
PDF

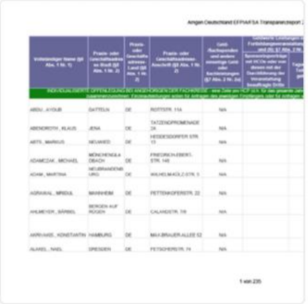

de__amgen__2016.pdf
PDF

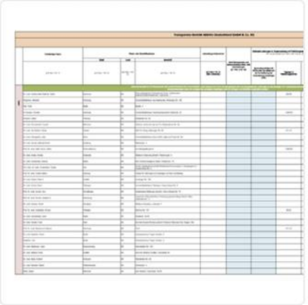

de__abbvie__2016.pdf
PDF

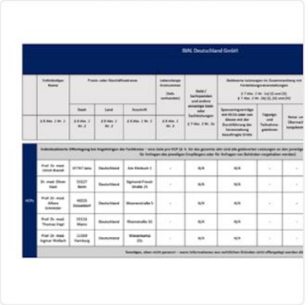

de__bial__2017.pdf
PDF

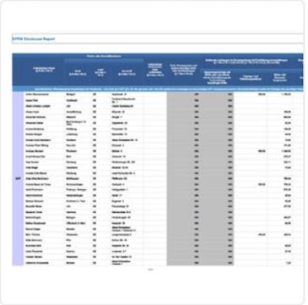

de__biogen__2017.pdf
PDF

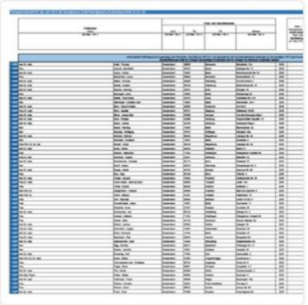

de__mundipharma__2016.pdf
PDF

Note

The screenshots were taken as part of the data curation process involved in creating the eurosfordocs.eu database.[2]

References

1. EFPIA. EFPIA Code of practice 2019 [Available from: https://www.efpia.eu/media/554677/efpia_codes_a5_v3-2021_sm.pdf.
2. Eurosfordocs.eu. Tech documentation, 2021 [Available from: eurosfordocs.eu/on-the-tech-side/tech-documentation/ accessed 4th January 2021.
