# Supplementary material for: Accessibility and quality of drug company disclosures of payments to healthcare professionals and organisations in 37 countries: a European policy review
Source: BMJ Open. 2021 Dec 16;11(12):e053138. doi: 10.1136/bmjopen-2021-053138 (PMC8679071; doi:10.1136/bmjopen-2021-053138)
Supplement: Supplementary data [file bmjopen-2021-053138supp004.pdf]

## Online Supplement 4. Approaches to data presentation decreasing the accessibility of payment disclosures

**Part A: Techniques used when publishing payment data on individual company websites**

1. Publish data as PDF documents (and not csv or XLS files) to make analysis difficult (example: most companies throughout Europe, except where the pharmaceutical industry trade groups created centralised databases).
2. Make the PDF document available only in an online viewer rather than as a separate file to prevent it from being downloaded for analysis (example: Menarini Switzerland).
3. Publish PDF documents consisting of images (and not text) to prevent any searches within the data (e.g. for recipient names) (example: Pfizer Sweden).
4. Reduce the resolution of image-based PDF documents to make them *almost* unreadable without constantly zooming in and out (example: Novo-Nordisk Spain).
5. Create PDF documents with repeated table headers occupying most of each page in the disclosure report. In some cases, if the content is image-based, the resulting PDF can exceed 1800 pages, and over 350 MB, which discourages users from opening or downloading it (example: Novartis Italy).
6. Require users to follow a lengthy process of accepting the “Terms of use” of the disclosed information to discourage engagement with the data (example: Pfizer Spain).

Note: Different techniques can be combined. For example, the disclosure report can be made available only in an online viewer (2), with each page published as an image (3) and in a low resolution (4) (example: Roche Italy).

**Part B: Techniques used when publishing payment data in centralised databases.**

1. Require users to follow a lengthy process of accepting the “terms of use” of the disclosed information to discourage engaging with the data (example: the Czech Transparentní spolupráce database).
2. Enable searching only for specific recipients, without the possibility of searching for companies or recipient categories (e.g. medical specialties) (the Czech Transparentní spolupráce database).
3. Make searches conditional on obtaining recipient ID numbers from another website (example: the Czech Transparentní spolupráce database)
4. Do not include the possibility of downloading the database as a single file to prevent analysis (Examples: the Irish Transfer of Value database – and all centralised industry platforms except Disclosure UK)
