# Supplementary material for: Accessibility and quality of drug company disclosures of payments to healthcare professionals and organisations in 37 countries: a European policy review
Source: BMJ Open. 2021 Dec 16;11(12):e053138. doi: 10.1136/bmjopen-2021-053138 (PMC8679071; doi:10.1136/bmjopen-2021-053138)
Supplement: Supplementary data [file bmjopen-2021-053138supp005.pdf]

Online supplement 5. Eurosfordocs.eu – database summary (2017-2019)[1]

Country	Disclosure reports	Successfully extracted (parsed) disclosure reports	Parse ratio	Companies associated with parsed disclosure reports	Number of payments to healthcare professionals and organisations	Value of payments (€)[2]
UK	1[3]	1	100%	141	164,112	1,771,785,871
Germany	112	89	79%	32	103,477	1,524,231,568
Spain	60	48	80%	16	370,444	959,704,223
Italy	60	57	95%	19	143,244	954,063,974
Switzerland	138	117	85%	41	36,503	471,638,889
Sweden	184	168	91%	68	15,434	249,913,018
Ireland	1[3]	1	100%	46	18,312	97,259,959
Total	556	481		160	851,526	6,028,597,501

Notes.

[1] – All data is accurate as of January 2021. Eurosfordocs.eu is updated regularly to reflect occasional changes in disclosure reports published by drug companies.

[2] – All payment values in non-euro currencies were converted to euros based the exchange rate obtained from the CurrencyConverter [1] a Python library for exchange rates.

[3] – The UK and Ireland are the only countries reported in the table in which all drug company payments are included in a single database. In all other countries, disclosure reports are published on individual websites for each company.

References

1. CurrencyConverter. CurrencyConverter 0.14.4 2020 [Available from: https://pypi.org/project/CurrencyConverter/ accessed 19th January 2021.
