# Supplementary material for: Accessibility and quality of drug company disclosures of payments to healthcare professionals and organisations in 37 countries: a European policy review
Source: BMJ Open. 2021 Dec 16;11(12):e053138. doi: 10.1136/bmjopen-2021-053138 (PMC8679071; doi:10.1136/bmjopen-2021-053138)
Supplement: Author's manuscript [file bmjopen-2021-053138.draft_revisions.pdf]

BMJ Open

BMJ Open is committed to open peer review. As part of this commitment we make the peer review history of every article we publish publicly available.

When an article is published we post the peer reviewers' comments and the authors' responses online. We also post the versions of the paper that were used during peer review. These are the versions that the peer review comments apply to.

The versions of the paper that follow are the versions that were submitted during the peer review process. They are not the versions of record or the final published versions. They should not be cited or distributed as the published version of this manuscript.

BMJ Open is an open access journal and the full, final, typeset and author-corrected version of record of the manuscript is available on our site with no access controls, subscription charges or pay-per-view fees (http://bmjopen.bmj.com).

If you have any questions on BMJ Open's open peer review process please email info.bmjopen@bmj.com

BMJ Open

Transparency in name only? Examining the accessibility and quality of drug company payment disclosures in Europe

Journal:	*BMJ Open*
Manuscript ID	bmjopen-2021-053138
Article Type:	Original research
Date Submitted by the Author:	05-May-2021
Complete List of Authors:	Ozieranski, Piotr; University of Bath, Department of Social and Policy Sciences<br>Martinon, Luc; Euros for Docs<br>Jachiet, Pierre-Alain; Euros for Docs, Department of Social and Policy Sciences<br>Mulinari, Shai; Lunds Universitet, Sociology
Keywords:	ETHICS (see Medical Ethics), Health policy < HEALTH SERVICES ADMINISTRATION & MANAGEMENT, Protocols & guidelines < HEALTH SERVICES ADMINISTRATION & MANAGEMENT

SCHOLARONE™
Manuscripts

I, the Submitting Author has the right to grant and does grant on behalf of all authors of the Work (as defined in the below author licence), an exclusive licence and/or a non-exclusive licence for contributions from authors who are: i) UK Crown employees; ii) where BMJ has agreed a CC-BY licence shall apply, and/or iii) in accordance with the terms applicable for US Federal Government officers or employees acting as part of their official duties; on a worldwide, perpetual, irrevocable, royalty-free basis to BMJ Publishing Group Ltd ("BMJ") its licensees and where the relevant Journal is co-owned by BMJ to the co-owners of the Journal, to publish the Work in this journal and any other BMJ products and to exploit all rights, as set out in our licence.

The Submitting Author accepts and understands that any supply made under these terms is made by BMJ to the Submitting Author unless you are acting as an employee on behalf of your employer or a postgraduate student of an affiliated institution which is paying any applicable article publishing charge ("APC") for Open Access articles. Where the Submitting Author wishes to make the Work available on an Open Access basis (and intends to pay the relevant APC), the terms of reuse of such Open Access shall be governed by a Creative Commons licence – details of these licences and which Creative Commons licence will apply to this Work are set out in our licence referred to above.

Other than as permitted in any relevant BMJ Author's Self Archiving Policies, I confirm this Work has not been accepted for publication elsewhere, is not being considered for publication elsewhere and does not duplicate material already published. I confirm all authors consent to publication of this Work and authorise the granting of this licence.

For peer review only

Transparency in name only? Examining the accessibility and quality of drug company payment disclosures in Europe

Piotr Ozieranski [1]
Luc Martinon[2]
Pierre-Alain Jachiet[2]
Shai Mulinari [3]

[1] Department of Social and Policy Sciences, University of Bath
[2] Euros for Docs
[3] Department of Sociology, Lund University

Correspondence to:
Piotr Ozieranski
Department of Social and Policy Sciences
University of Bath
BA2 7AY
Email: p.ozieranski@bath.ac.uk
Phone: +44 (0) 1225 384204

Key words: pharmaceutical industry, self-regulation, public regulation, transparency, financial disclosure, conflicts of interest

Wordcount: 4985

For peer review only

Abstract

Objectives: To examine the accessibility and quality of drug company payment data in European countries with different approaches to disclosure.

Design: Comparative policy analysis of payment data disclosed by drug companies, industry trade groups, and public or public-private authorities.

Setting: 37 European countries.

Participants: European Federation of Pharmaceutical Industries and Associations, its national trade group and drug company members; eurosfordocs.eu, an independent database integrating company payments from seven countries; public and public-private authorities managing payment disclosure.

Main outcome measures: Regulatory approaches to payment disclosure (self-regulation, public regulation, mix of the two); data accessibility (format, structure, searchability, summary statistics, downloadability) and quality (range of disclosed information, payment aggregation, inclusion of taxes, recipient or donor identifiers).

Results: Of 30 countries with self-regulation five had centralised databases, of which only one, Disclosure UK, had considerably higher data accessibility and quality than others. In 23 countries with self-regulation, disclosures were published as PDFs on individual company websites, preventing members of the public from being able to understand payments comprehensively. Eurosfordocs.eu had greater accessibility than any industry-run database and integrated between 56% and 100% of the value of payments in countries with relevant data available. Nevertheless, eurosfordocs.eu shared key quality shortcomings with the underlying industry data, including ambiguities in identifying payments and their recipients. Of 15 countries with public regulation or a mix of self- and public regulation 13 had centralised databases, which had widely ranging accessibility and quality, and some of the same shortcomings as industry-run databases. The French database, Transparence Santé, had clearly the highest data accessibility and quality, also exceeding that of Disclosure UK.

Conclusions: Payment disclosure itself does not equal transparency. Without improving the transparency of payment data, it cannot be used effectively to investigate conflicts of interest. Some improvements quality are easily achievable while others will require major regulatory change.

Strengths and limitations

- We investigate the quality and accessibility of drug company payment disclosure data in 37 European countries.
- We use a set of measures relevant for countries with industry self-regulation, public regulation, and a combination of the two.
- We present our results as a “heat map” showing the least and most problematic aspects of payment data accessibility and quality.

- One key limitation is that that we did not quantify some aspects of the accessibility and quality of payment data.

For peer review only

1. Introduction

Conflicts of interest (COIs) can bias healthcare research, practice, education, and policy.[1-3] The last decade has seen a global trend towards addressing concerns about COIs by publishing drug company payments to the healthcare sector.[4-8] It is best exemplified by the US Sunshine Act, establishing Open Payments, a database which has triggered extensive research on payment distribution,[9 10] and its links with drug prescription[11] and cost.[12 13] Open Payments increases transparency of COIs by enabling cross-checking information collected by professional organisations,[14] conference organisers,[15] and scientific journals.[16] It also aids investigations into undue influence or corruption by helping identify unusual payment patterns.[17 18]

Unlike the US, in most European countries payments to healthcare professionals and organisations are disclosed via industry self-regulation, managed by the European Federation of Pharmaceutical Industries and Associations (EFPIA) and its national trade group members.[4 6] Only a few countries, including France, Portugal and Latvia, regulate payment disclosure using government regulation.[4 6] Notwithstanding regulatory approaches taken in specific countries, payment distribution has only been mapped separately in the UK[19 20] and Germany,[21] and comparatively in the UK, Germany, Sweden, Switzerland, Italy, Ireland and Spain.[22] However, France is the sole country where relationships with prescription have been investigated.[23]

Limited payment data scrutiny has detrimental impact on transparency. Notably, corrupt relationships identified via official investigations pertaining to Greece,[24] Poland and Russia[25] might have been revealed earlier by examining payment patterns, following the US' example.[17 18] Similarly, discrepancies in payments reported separately by companies and some healthcare providers[26] and commissioners[27] in England suggest that many organisation-level COIs remain undetected, potentially also elsewhere in Europe.

The likely reasons behind the limited research on payment data are its low accessibility and quality. Regarding accessibility, a study using a convenience sample of nine European countries has found that of six countries with self-regulation five lacked centralised payment databases.[4] The dispersal of disclosures on multiple drug company websites has been identified as an obstacle in examining payments in Germany, one of the countries from the EU sample.[21 28] A recent independent initiative by "activist data scientists" seeking to enhance accessibility of payment data involved creating a database called eurosfordocs.eu. Drawing on the example of a similar database in Germany,[21] eurosfordocs.eu integrates data disclosed separately by many companies in countries pursuing self-regulation.[22 29] Contrastingly, of the four countries pursuing government regulation alone or in combination with self-regulation three had databases integrating payments reported by all companies.[4]

A related aspect of low accessibility – both in countries with self-regulation and government regulation – is the user interface.[4] Of the six European countries with self-regulation only Disclosure UK, the database run by the Association of the British Pharmaceutical Industry (ABPI), was judged as user friendly.[4] However, of the three databases in countries with public regulation or a mix of government and self-regulation the Dutch and Portuguese

For peer review only - http://bmjopen.bmj.com/site/about/guidelines.xhtml

databases were described as "partially" user friendly, while the French was deemed "not" user friendly.[4] Importantly, challenges in the interface of the French database were only addressed by an independent platform called eurosfordocs.fr, stimulating several high-profile journalistic investigations into COIs.[30-32]

Data quality is the second problem potentially constraining disclosure-based research. However, it has only been examined in countries pursuing self-regulation. For example, analyses of Disclosure UK revealed inconsistencies in reporting of payment values and recipients,[19 20] compounded by the absence of unique recipient identifiers.[33] Yet these issues have not been addressed in the newest edition of the ABPI's Code of Practice, which governs payment disclosure by its member companies.[34] Similar shortcomings, including duplicate entries, were identified in German data,[21] indicating that they might characterise self-regulation more broadly.

Therefore, important gaps exist in our understanding of the accessibility and quality of European payment data. First, ongoing debates on the introduction of public regulation in some countries[5] suggest that the only comprehensive European overview of approaches to payment disclosure[6] might have missed key regulatory developments, potentially with implications for data accessibility and quality.

Second, the EFPIA Code of Practice (hereafter: EFPIA Code),[28] sets some common requirements for data accessibility and quality, but it is unclear how closely they are followed in practice by its member trade groups. Existing research suggests that although some trade groups only meet the minimum standards (e.g. by publishing data on their websites), others might exceed them (e.g. by creating centralised databases).[4] The need for establishing a comprehensive European pattern of compliance with EFPIA's self-regulatory rules regarding payment disclosure is underscored by studies of self-regulation of drug marketing in Sweden and the UK suggesting failure to meet some of its own key promises.[35 36]

Third, while some shortcomings of data quality have been identified in the independent database integrating payments made by companies in Germany,[21] no similar evaluation has been undertaken in relation to eurosfordocs.eu, the largest database of this kind in Europe.

Fourth, regulatory approaches in many European countries have escaped scrutiny[4] and therefore it is unclear whether payment data reported in these countries shares the strengths and weaknesses identified elsewhere. Consequently, although some aspects of government regulation, such as full disclosure by individual health professionals, have been demonstrated as superior in relation to self-regulation,[4 6] we cannot conclude definitively whether this is reflected by payment data accessibility or quality.[4]

Finally, criteria used to evaluate data accessibility and quality need to be expanded and refined, as some of them, such as "user-friendliness" have attracted a contrasting appraisal of the same disclosure database by other expert commentators.[4 37]

The dearth of comparative research on the accessibility and quality of payment disclosures has important practical implications. Crucially, it means that most European countries are

For peer review only - http://bmjopen.bmj.com/site/about/guidelines.xhtml

likely to develop their government regulation in isolation, and the limited policy learning and diffusion risks repeating mistakes made by others or not sharing effective and efficient solutions. Notably, most countries brought in relevant government regulation around when the Sunshine Act was introduced in the US and the self-regulation was adopted in Europe and much fewer have adopted it recently.[4] Similarly, EFPIA has only recently started collecting summary statistics from countries pursuing self-regulation, without, however, covering accessibility or quality.[38]

We have two objectives. First, to identify regulatory approaches to drug company payment disclosure in European countries, including specific arrangements for making payments available to the public. Second, to examine the accessibility and quality of payment data disclosed via self-regulation, integrated within eurosfordocs.eu, and disclosed via public regulation.

2. Methods

2.1. Data collection

2.1.1. Identification of regulatory approaches

To identify regulatory approaches to payment disclosure in Europe, PO and LM first identified available peer-reviewed English-language research on the regulation of drug company payment disclosure. To this end, we searched Scopus using the terms “Sunshine Act”, “Open Payments”, as well as “European Federation of Pharmaceutical Industries and Associations” and “EFPIA” combined with “disclosure”. We applied the same terms in the Google search engine to identify “grey literature”, including non-peer reviewed reports.

Second, PO and LM conducted iterative searches on websites dedicated to industry payment disclosure, including EFPIA’s website, the websites of its national trade group members, and industry transparency initiatives. We also examined the country profiles published by MediSpend[39] and the websites of four major companies with presence across Europe (Amgen, GSK, Merck Serono and Bayer) providing access to company disclosure methodologies reflecting local regulatory requirements. Finally, we considered the websites of public or public-private authorities which the previous steps identified as involved in regulating payment disclosure.

Third, PO surveyed industry trade groups and public or public-private bodies regulating payment disclosure (Online Supplement 1). The first round of standardised questions was emailed in mid-November 2020, followed up by reminder messages in late December 2020, asking recipients to provide answers by the end of the 1st week of January 2021. Of 34 approached pharmaceutical trade groups 17 replied. Of those, 14 answered at least some of the questions, while the remaining ones sent holding messages. Of 13 approached public or public/public-private bodies ten replied. Of those, six answered at least some of the questions, three sent holding messages, and one redirected us to another institution.

For peer review only - http://bmjopen.bmj.com/site/about/guidelines.xhtml

2.1.2. Data on accessibility and quality of payment disclosures

First, in countries with self-regulation of payment disclosure, we considered industry codes of conduct, reports, press releases, trade group websites and industry-run databases. Second, LM and PAJ generated observations when collecting data from individual company websites for the purposes of creating eurosfordocs.eu, as detailed elsewhere.[22] Third, in countries with disclosure regulated by public or public-private bodies the data included relevant legislation, the websites of bodies managing payment disclosure, and disclosure databases. Fourth, in both countries pursuing self-regulation and public regulation we considered responses from our email survey. Finally, in countries covered by eurosfordocs.eu we also collected – for verification purposes – national-level summary statistics published by EFPIA, industry trade groups, and survey responses from the trade groups.

2.2. Data analysis

2.2.1. Content analysis

Most of the source material, including industry codes and legislation, was available in English. If this was not the case, we translated it to English using Google Translate and Deepl.com. We clarified any linguistic issues by cross-checking with other online sources and consulting with relevant national bodies and colleagues with language expertise.

The regulatory approaches were coded deductively building on an earlier categorisation which distinguished countries pursuing government regulation, industry self-regulation, government regulation existing in parallel to self-regulation, and a mix of the two approaches.[4] We modified this categorisation in light of new regulatory developments, such as the 2016 decision by the Spanish Data Protection Agency[40] making payment disclosure by healthcare professionals compulsory without introducing new government regulation.[22] Specifically, we replaced the "government regulation" category with a new one called "public regulation", comprising government regulation and "regulatory intervention" understood as decisions by data protection agencies.

For the most part, we coded the data deductively. Codes relating to data accessibility were developed based on a categorisation used in earlier comparative European research.[4] Codes relating to data quality were also developed deductively using earlier research findings[20 5] and observations involved in creating eurosfordocs.eu. Inductive coding was only applied in relation to the types of disclosed information and company techniques of decreasing data accessibility as we lacked a pre-existing code list.

The data was coded by PO. We validated the results of the coding by discussions within the research team and resolved any differences by agreement. In developing our analysis we set the characteristics of disclosed data against recommendations from the EFPIA Code. This was not necessary in relation to government regulation or regulatory interventions as they did not introduce any optionality.

For peer review only - http://bmjopen.bmj.com/site/about/guidelines.xhtml

2.2.2. *Descriptive statistical analysis*

As eurosfordocs.eu involved data extraction using disclosures published by individual companies and industry trade groups we estimated the match between the database and the underlying data by comparing the value of payments calculated in specific countries using eurosfordocs.eu with national-level summaries obtained from industry sources.

2.2.3. Outcome measures

We had one outcome measure denoting a regulatory approach to payment disclosure in each country – self-regulation; public regulation pursued either as government regulation or regulatory intervention; a mix of self-regulation and public regulation. As we considered whether self-regulation and public regulation were pursued in parallel, some countries were coded as having both self- and public regulation.

The measures of accessibility and quality reflected the heterogeneity of the forms of payment data presentation. The basic measure of accessibility applied in *all countries* was whether it was disclosed on a centralised database or multiple websites. In addition, for *countries with centralised databases,* we created a "heat map" of accessibility and quality measures to aid data synthesis and interpretation (Table 1).

For peer review only

For peer review only - http://bmjopen.bmj.com/site/about/guidelines.xhtml

Table 1 The heat map of measures of accessibility and quality of payment databases.

Measures of payment data accessibility

		**Higher accessibility**	**←--------------→**	**Lower accessibility**
Database format	How is the database published (e.g. PDF, XLS, CSV, embedded within websites)	Webpage or XLS	Readable PDFs	Image-based PDFs
Database structure	Does the data from all companies follow a single template consistently?	Yes	N/A	No
Database searchability	Can the database be searched? If so, can database searches be carried out without data users providing any additional information?	Yes	Database searchable but additional information needed for searches	No
Summary statistics	Does the database offer users the possibility of generating real-time, dynamic data summaries based on selected database characteristics?	Yes	N/A	No
Downloadability	Can the database be downloaded as a single csv or Excel file for further analysis?	Yes	N/A	No

Measures of payment data quality

		**Higher quality**	**←--------------→**	**Lower quality**
Range of disclosed information	What information is included in relation to donors, recipients and payments?	Webpage or XLS	Readable PDFs	Image-based PDFs
Aggregation of payments	Are payments reported individually (i.e. all payments have separate entries in disclosure documents) or are they aggregated on an annual basis (e.g. per recipient and/or payment category)?	Yes	N/A	No
Inclusion of taxes	Is it clear whether payments are reported inclusive or exclusive of any taxes?	Unclear	No single rule, decided by individual companies, , can be established based on separate documentation	Single rule
Unique identifiers	– do reported donors or recipients have unique identifiers?	All donors or recipients	Some donors or recipients	No unique identifiers

For peer review only - http://bmjopen.bmj.com/site/about/guidelines.xhtml

On top of the measures included in Table 1 we had one additional measure of quality related to *eurosfordocs.eu* given its nature as a database derived from payment disclosures published by drug companies and industry trade groups. Specifically, we examined how closely it matched the value of payments reported in industry data.

Finally, in countries pursuing *self-regulation without centralised databases* we examined whether industry trade groups created gateways leading to disclosure documents. To further illustrate challenges in data accessibility in these countries we also generated lists of examples of, first, deviations from the data presentation format recommended by EFPIA (called "EFPIA disclosure template"[28]) and, second, the ways of presenting payment data which decreased its accessibility. As these problems were clearly widespread and serious we did not measure them quantitatively; instead, we documented their nature and implications for data users.

2.3. Ethics

This study did not require a full ethics approval as no individual payment data was processed. The study's ethical implications of this study were approved via a peer ethics review process at the Department of Social and Policy Sciences, University of Bath in April 2016.

2.4. Patient and public involvement

We did not involve patient groups or the public. Our policy recommendations seek to increase public engagement with payment data by enhancing its accessibility and quality.

3. Results

We first map the regulatory approaches to payment disclosure in Europe. We then examine the accessibility and quality of payment data published by pharmaceutical companies and trade groups under the EFPIA Code. Subsequently, we consider industry data integrated within eurosfordocs.eu. Finally, we analyse data disclosed within public regulation.

3.1. Mapping regulatory approaches to payment disclosure

We identified *self-regulation* in 30 countries (Online Supplement 2). Of those, in all but two, industry trade groups adopt the provisions of the EFPIA Code[28 41] as EFPIA members. In addition, while the Luxembourgish trade group is not an EFPIA member, its member companies also disclose payments following the EFPIA Code. Also, the Danish Denmark trade group developed self-regulation covering "grants and donations" to hospitals,[42] which is separate from the EFPIA Code. In 22 of the 30 countries, self-regulation is the sole regulatory approach, while in the remaining 8 it functions in parallel with public regulation.

For peer review only - http://bmjopen.bmj.com/site/about/guidelines.xhtml

We found *public regulation* of payment disclosure in 12 countries, taking the form of government regulation, while in Greece, an additional regulatory intervention took place in the form of an interpretation of the government regulation by the Data Protection Agency.[43] Only in France, Portugal and Turkey public regulation is the exclusive regulatory approach, while in the remaining 8 countries self-regulation exists in parallel. In 4 of these countries (Denmark, Lithuania, Romania, and Slovakia), self- and public regulation cover different donors, payments or recipients, whereas in the remaining ones (Estonia, Greece, Hungary and Latvia) donors, recipients and payments disclosed via public and self-regulation may overlap.

Self- and public regulation are combined in four countries. In two of these countries, public regulation takes the form of government regulation (Belgium and Finland) or and in two other – regulatory intervention (Spain and the Netherlands). Specifically, Belgium regulates payment disclosure via a "Sunshine Act" but the interpretation of its key provisions has been left to betransparent.be, a multi-stakeholder platform involving industry and professional organisations,[44 45] which also runs the Transparency Register integrating company disclosures. [46 47] In Finland, the Medicines Act stipulates that drug companies "must keep available for public review" a list of all payments to "associations in the fields of medicine and health care" [48] but in practice the disclosure takes place following the EFPIA Code.

In Spain, public regulation involves an intervention by the Data Protection Authority[40] confirming that the publication of named payment recipients does not require recipient consent.[22] Like in Belgium, disclosure is managed by companies following the EFPIA Code. In the Netherlands, payments are disclosed using self-regulatory rules developed by the Foundation for the Code for Pharmaceutical Advertising. Like in Belgium, the central platform involves multistakeholder collaboration, in this case between the industry and healthcare providers.[49] However, state authorities triggered the policy debate on payment disclosure and, having considered self-regulation preferable to public regulation, they lent it financial support and monitor its performance.[50 51] Like in Spain, the Dutch Data Protection Authority confirmed that recipient consent is not required.[51]

3.2. Data disclosed via self-regulation by pharmaceutical companies and trade groups

We were able to collect information on accessibility and quality of payment data in 28 of the 30 countries pursuing self-regulation. Regarding data accessibility, the EFPIA Code allows companies within each country to disclose payments either on a centralised platform or individual websites.[28] However, only 5 trade groups have established databases for all companies, including four countries following the EFPIA Code and one drawing on its own Code of practice (LIF in Denmark).

The most frequent problems identified across all the industry databases were the lack of summary statistics or donor or recipient identifiers. However, the databases differed considerably, with Disclosure UK, run by the ABPI displaying decisively the highest overall accessibility, while the one managed by the Hellenic Association of Pharmaceutical

For peer review only - http://bmjopen.bmj.com/site/about/guidelines.xhtml

Companies (SFEE) – the lowest (Table 2). The differences in data quality were lower, with the SFEE database having the highest, and the Irish database, the lowest data quality. Overall, Disclosure UK had the highest combined data accessibility and quality, while the SFEE database – the lowest.

For peer review only

Table 2 Accessibility and quality of drug company payment data disclosed via centralised industry databases and eurosfordocs.eu[1]

For peer review

			Payment data accessibility					Payment data accessibility			
	**Name of regulation**	**Overseeing authority and web link to disclosure database[2, 3]**	**Disclosure document format**	**Single data template**	**Database searchable**	**Customisable summary statistics included in database**	**Database downloadable for further analysis**	**Information included**	**Aggregation of payments**	**Payments with or without taxes**	**Unique identifiers**
**Self-regulation at the European level**											
EFPIA	EFPIA HCP/HCO Disclosure Code (2014), subsequently subsumed under EFPIA Code	EFPIA	Not regulated	Yes ("EFPIA disclosure template"), but deviations allowed	Not regulated	Not regulated	Not regulated	Donors; recipients; recipient location; payment categories and amounts, year	Annually per payment type	No single rule, decided by individual companies	Optional
**Centralised online industry databases[2]**											
UNITED KINGDON	ABPI Code of Practice	[Association of the British Pharmaceutical Industry]()	Website, XLS	Yes	Yes	No	Yes	Donors; recipients; recipient categories (healthcare professionals but not organisations) and location; categories payment categories and amounts, web links with further	No for healthcare organisations; Annually per payment type for healthcare professionals	No single rule, decided by individual companies, , can be established based on separate documentation	No

For peer review only - http://bmjopen.bmj.com/site/about/guidelines.xhtml

								descriptions for some payments			
CZECH REPUBLIC	Eticky Kodex AIPF	Asociace inovativního farmaceutického průmyslu	Website	Yes	Yes (but donor or recipient identifiers needed)	No	No	Donors; donor location, recipients; recipient location; payment categories and amounts, year	Annually per payment type	No single rule, decided by individual companies, , can be established based on separate documentation	Yes
DENMARK	Ethical rules for the pharmaceutical industry's donations and grants	Lægemiddelindustriforeningen	Readable PDFs	Yes	No	No	Yes	Donors; project name; recipient, product name; funded activity; payment goal; timescale of funded activity, payment amount and form (cash or benefit in kind)	No	Unclear	No
GREECE	SFEE Code of Conduct	Hellenic Association of Pharmaceutical Companies	Website	Yes	No	No	No	Donors; recipients; recipient categories; payment descriptions, categories goals, and amounts; date	No	No single rule, decided by individual companies, , can be established based on separate documentation	No
IRELAND	Code of Practice of the Pharmaceutical Industry	Irish Pharmaceutical Healthcare Association	Website	Yes	Yes	No	No	Donors; recipients; recipient location;	Annually per payment type	No single rule, decided by individual companies, ,	No

								payment categories and amounts		can be established based on separate documentation	
**Industry data integrated within an independent database[2]**											
EUROSFORDOCS.EU	Codes of conduct in countries where data was collected (Ireland, Italy, Germany, Spain, Sweden, Switzerland and the UK)	N/A	Website, XLS	Yes	Yes	Yes	Yes	Donors; recipients; recipient location; payment categories and amounts, year	No for UK healthcare organisations ; Annually per payment type for healthcare professionals and healthcare organisations from other countries	No single rule, decided by individual companies, , can be established based on separate documentation	Spain: yes Rest: no

Notes

[1] – Lighter colours indicate, respectively, higher, and darker colours – lower, data accessibility and quality.

[2] – The disclosure requirements ordinarily cover both healthcare professionals and organisations. The exceptions are the database run by the Danish pharmaceutical industry trade group (donations to hospitals) and the database run by the Greek pharmaceutical industry trade group (only payments to healthcare organisations).

[3] – Web links are accurate as of May 2021.

For peer review only - http://bmjopen.bmj.com/site/about/guidelines.xhtml

In 23 of the remaining countries with self-regulation, disclosures were published on individual websites for each company. In 18 of these countries, trade groups have created gateways to these websites, as recommended by EFPIA.[28] Nevertheless, without *explicit guidance* from EFPIA on the electronic format of disclosure documents in countries with or without gateways disclosures published on company websites were typically PDFs, minimising possibilities for searching through and integrating data from different companies. In addition, some companies presented data without strictly following the EFPIA-recommended "disclosure template"[28], which, again, impeded possibilities for cross-company comparisons (see Online Supplement 3 for examples of these deviations). Some firms apparently manipulated data presentation further, for example, using low-resolution, image-based PDFs, which prevented any searches (see Online Supplement 4 for a summary).

Given the problems identified above, assessing the quality of payment data was practically impossible in countries without centralised databases. Therefore, we do this in the context of eurosfordocs.eu, a database covering drug company disclosures made in countries pursuing self-regulation (Ireland, Italy, Germany, Sweden, Switzerland, and the UK) or a mix of self-and public regulation (Spain).

3.3. Industry data disclosed integrated within eurosfordocs.eu

Eurosfordocs.eu clearly had data accessibility superior to the industry-run databases (Table 2). While the Irish and UK databases were also searchable, eurosfordocs.eu offered many customisable queries using combinations of involving donors and recipient names and payment categories. [52] It was also the only database offering (customisable) summary statistics allowing users to explore the data further. In addition, beyond Disclosure UK it was the only downloadable database for further analysis.

A specific consideration regarding the quality of data included in eurosfordocs.eu is how closely it matches the underlying industry disclosures. Complete data extraction was only possible in the UK and Ireland, two countries with centralised industry-run databases (see Online Supplement 5 for summary data extraction statistics). Elsewhere data scraping prioritised the 20 largest donors known from the countries with complete data; more data was scraped whenever formats used by companies made it possible.[22] Nevertheless, except for Spain, a country with a high proportion of image-based PDFs hindering data extraction, the resulting dataset closely matched the industry's summary country-level data[53] (Table 3).

Table 3 Estimation of the comprehensiveness of industry payment data extracted for eurosfordocs.eu (2019)

Country[1]	Total value of payments reported in industry summary data (€m)[2, 3]	Total value of payments extracted to eurosfordocs.eu (€m)[4, 5]	Difference (€m)	Difference as a share of industry summary data (%)[6]
GERMANY	629	499	130	21%
IRELAND	35	35	0	0%
SWEDEN	90	82	8	9%
SWITZERLAND	167	155	12	7%
SPAIN	601	337	264	44%
UK	619	611	8	1%[5]

[1] – Only countries covered by both eurosfordocs.eu and available national-level summary data generated by industry trade groups are included.
[2] – Sources of national-level summary payment data.

- Germany,[54] Spain,[55] Switzerland[56] – publicly available pharmaceutical industry summary data published by the pharmaceutical industry trade groups.
- Ireland – a combination of an Europe-wide report published by EFPIA[38] and email communication with the Irish pharmaceutical industry trade group. [57]
- Sweden – email communication with the pharmaceutical industry trade group.
- The UK - calculations based on data obtained from Disclosure UK, the centralised database of industry payments run by the Association of the British Pharmaceutical Industry.[58]

[3] – All payment values in non-euro currencies were converted to euros based on the average yearly exchanged rates published by the European Central Bank.
[4] – The source of payment values reported in this column are centralised pharmaceutical industry payment databases (Ireland and the UK) and payment reports covering payments made by individual companies (Germany, Spain, Sweden and Switzerland).
[5] – All payment values in non-euro currencies were converted to euros based the exchange rate obtained from the CurrencyConverter,[59] a Python library for exchange rates.
[6] – Some of the difference between the value of payments based on summary industry data and extracted to eurosfordocs.eu results from the differences in the exchange rates. This is exemplified by the examples of Ireland (both values in euro, no difference) and the UK (original values in the sterling, the difference is caused by different exchange rates used to convert the sterling to euro).

For peer review only - http://bmjopen.bmj.com/site/about/guidelines.xhtml

Nevertheless, other aspects of the quality of data in eurosfordocs.eu have important limitations determined by the underlying company disclosures.

First is the limited amount of disclosed information. For example, eurosfordocs.eu does not present payment distribution within the healthcare system due to the incoherent use or omission of recipient categories by drug companies. Specifically, of all countries covered by eurosfordocs.eu the UK is the only one where the industry trade group categorised healthcare professionals receiving payments,[60] albeit incoherently;[19] healthcare organisations were nowhere categorised.

Second, consistent with the EFPIA Code [28] payments to healthcare professionals ought to be aggregated annually per recipient within each payment category. The same applies to payments to healthcare organisations, with the exception of the UK, where the ABPI mandates that payments to healthcare organisations be reported individually.[60] This UK-specific rule might explain the large difference in the number of payments reported with Germany, a country with a similar overall value of payments (Online Supplement 5). However, it is equally possible that not all companies in the remaining six countries aggregate payments consistently as some list more than one payment per recipient, which might also indicate that although these recipients have the same names, they are different entities.

Third, the reported payment values must be interpreted cautiously as it is unclear whether they include VAT or other taxes without consulting documentation ("methodological notes") which the EFPIA Code requires to be published separately by each company.[28]

Finally, while EFPIA introduces the option of unique recipient identifiers in disclosed payment data [28], only the Spanish industry trade group followed this recommendation. In the remaining countries, the number of recipients per company and, consequently, the value of payments per recipient remains unknown. As companies apply inconsistent naming approaches in disclosures made by the same or different companies the same recipient can have different names, and, conversely, different recipients may have the same name. Further, the same recipient can also be identified at different levels of aggregation (e.g. hospital wards, departments or hospitals), with self-regulation at least in some countries placing the onus of identifying multiple records on payment recipients and not companies.[61] More broadly, without identifiers payment data cannot be connected to other databases, which prevents studying, for example, associations between payment and prescription patterns.

3.4. Data disclosed via public regulation or a mix of public and self-regulation

Of the 15 countries with public regulation or a mix of self-and public regulation, all but two had centralised databases. The exceptions were Finland and Spain, where disclosures were made on individual drug company websites, consistent with the requirements of the EFPIA Code. Of the thirteen countries with centralised databases one had a database which was not publicly available (Turkey) and two others had separate databases for different payment

For peer review only - http://bmjopen.bmj.com/site/about/guidelines.xhtml

categories (Denmark) and healthcare professionals and organisations (Greece). As the information included in the two databases in Denmark and Greece does not differ in the context of our outcome measures, we consider them jointly (Table 4).

For peer review only

For peer review only - http://bmjopen.bmj.com/site/about/guidelines.xhtml

Table 4 Accessibility and quality of drug company payment data disclosed via public regulation or a mix of self-regulation and public regulation[1]

	Name of regulation[2,3]	Overseeing authority and database web link[4]	Disclosure document format	Single data template	Database searchable	Customisable summary statistics	Database downloadable for further analysis	Characteristics included[5]	Aggregation of payments	Payments with or without taxes	Unique identifiers
FRANCE	Law No. 2011-2012 (Law Bertrand)	Ministry of Social Affairs and Health	Webpage	Yes	Yes	No	Yes	Donors; donor categories; recipients; recipient categories; payment categories and amounts; date; recipient address	No	Inclusive of VAT	Recipients (partial), donors (multiple entries for subsidiaries)
LATVIA	Regulation No. 378 (2014)	Health Inspectorate	XLS	Yes	No	No	Yes	Donors; recipients; recipient categories; payment name, description, category and amount; date; recipient address	No	Unclear	Donors, recipients
SLOVAKIA	Act No. 362/2011 on Medicines and Medical Devices	National Health Information Center	XLS	Yes	No	No	Yes	Donors; recipients; recipient categories (only healthcare professionals); payment descriptions, categories and amounts; clinical trial numbers; product names; recipient address; date	No	Unclear	Payments (clinical trial numbers)
LITHUANIA	Law on Pharmacy of the Republic of Lithuania (relevant provisions added in 2019), Ministerial Order No. V-1537 (2020)	State Medicines Control Agency	XLS	Yes	No	No	Yes	Donors; recipients; recipient categories; payment name; date; recipient address	No	Unclear	Recipients (publicly available), donors (collected but not publicly available)

For peer review only - http://bmjopen.bmj.com/site/about/guidelines.xhtml

PORTUGAL	Decree Law 20/2013 and 128/2013	National Authority of Medicines and Health Products	Webpage	Yes	Yes	No	No	Donors; donor categories; recipients; payment descriptions and amounts; years	No	Inclusive of VAT	No
ROMANIA	Orders of the Minister of Health 194/2015 and 874/2015	National Agency for Medicines and Medical Devices	Webpage	Yes	Yes	No	No	Donors; recipients; recipient categories; payment descriptions, categories, and amounts; recipient address; date	No	Unclear	No
BELGIUM	Sunshine Act of 2016	Federal Agency for Medicines and Health Products	Webpage	Yes	Yes	No	No	Donors; recipients; recipient categories; payment categories and amounts; recipient address; years	Annually per payment type	Unclear	Donors, recipients
DENMARK	Health Act of 2014, Executive Order No. 1153	Danish Medicines Agency 1) conferences abroad ; 2) professional affiliations	Webpage	Yes	Yes	No	No	Payments related to conferences abroad – donors; recipients; recipient categories; recipient address;; Payments related to professional affiliations – donors; recipients; recipient categories; recipient address; payment amounts	Annually per payment type	Unclear	Recipients
HUNGARY	Act XCVIII of 2006 (relevant provisions introduced in 2011)	National Institute of Pharmacy and Nutrition	Webpage	Yes	Yes	No	No	Donors; payment names, descriptions and amounts; date; recipient address	No	Unclear	No

THE NETHERLANDS	Code of Conduct for Pharmaceutical Advertising (2012)	Foundation for the Code for Pharmaceutical Advertising	Webpage	Yes	Yes (but recipient identifiers needed)	No	No	Donors; recipients; recipient categories; payment categories and amounts; year	Annually per payment type	Unclear	Recipients
ESTONIA	Medicinal Products Act of 2005 (relevant provisions introduced in 2013)	State Agency of Medicines	XLS	Yes	No	No	Yes	Donors; payment categories and amounts; payment location (country), year	Annually per donor	Unclear	No
GREECE	Law 4316/2014; Opinion No. 5/2016 and 2/2017 of the Hellenic Data Protection Authority; circular No. 17770/2016 of the National Authority for Medicines	National Organisation for Medicines 1) payments to individual conference participants; 2) payments to organisational conference organisers; individual drug company websites	PDFs – image-based	Yes	No	No	Yes	Payments to individual conference participants - donors, recipients, payment categories (types of conference expenditure) and amounts; year Payments to organisational conference organisers – donors; payment amounts; year	Payments to individual conference participants – annually per recipient; Payments to organisational conference organisers – donors	Unclear	Donors
TURKEY	Regulation on Promotional Activities of Medicinal Products for Human Use 2015	Ministry of Health (database not publicly available)	Unclear	Unclear	Unclear	Unclear	Unclear	Unclear	Unclear	Unclear	Unclear

Notes.

[1] – Lighter colours indicate, respectively, higher, and darker colours – lower, data accessibility and quality.

[2] – This column provides the dates when public regulation of payment disclosure was first introduced. If public regulation of payment disclosure forms part of a larger piece of government regulation, it is specified – where appropriate – whether the regulation of payment disclosure was introduced as a change already existing government regulation. The dates reported here do not cover changes to or refinements of provisions focusing on payment disclosure.

[3] – The disclosure requirements ordinarily cover both healthcare professionals and organisations. The exceptions are the Danish databases (only healthcare professionals) and the Turkish database (it is unclear whether disclosure requirements also cover healthcare organisations).

[4] – Web links are accurate as of May 2021.
[5] – The recipient addresses ordinarily refer to the location of the payment recipient. In the case of Hungarian, Latvian and Lithuanian databases we considered that the event addresses were equivalent to recipient addresses.

For peer review only

Regarding overall data accessibility, the French database, Transparence Santé, was a clear frontrunner. The remaining ones had varying strengths and weaknesses, with the lack of customisable statistics, searchability and downloadability being key problems. However, the databases all had similar overall accessibility, with the exception of the Greek database, which presented disclosure documents as image-based PDFs, precluding any searches or analysis. Transparence Santé also had the best data quality, although the distance between the other databases was smaller. These databases had a similar level of overall quality, with only five disclosing a greater range of donor or recipient characteristics than those covered by the EFPIA Code. The Estonian database was an outlier with the lowest quality based on our measures.

Transparence Santé had combined data accessibility and quality considerably exceeding that of Disclosure UK, the frontrunner industry database, with the Latvian and Lithuanian databases having similar overall levels of accessibility and quality.

4. Discussion

By examining 37 European countries we demonstrate that payment data disclosure does not automatically increase transparency of financial relationships between drug companies and the healthcare sector.[4 5] Consistent with research on disclosure of aspects of health policymaking by both public[6 57] and private-sector actors,[62] we find that achieving "practical transparency" is no less important than introducing transparency rules themselves.[63 64]

Although Open Payments has attracted some criticism,[65] its quality and accessibility is vastly superior to European data disclosed either via either self- or public regulation. Although EFPIA calls payment data generated via self-regulation "open to public scrutiny",[66] establishing the entanglement of any recipient, let alone a system-level picture, is impossible given the prevalent dispersal of disclosure documents on drug company websites. Importantly, disclosures published as PDFs fall below Australian industry-endorsed regulations requiring firms to use an analysable format.[5] Compared with previous research, little progress has been made in establishing centralised industry databases.[4] Consequently, self-regulation cannot address "the issues of perceived conflict of interest",[67] as promised by EFPIA.

Eurosfordocs.eu radically enhances data accessibility in countries without centralised industry databases by integrating disclosures made by multiple companies. By bringing together data from several countries, eurosfordocs.eu also enables comparative investigations of payment patterns reflecting the interconnected nature of European pharmaceutical markets and accelerating EU-wide health initiatives.[68] Although the customisable opportunities for data exploration are new to the public, they have been offered as a consultancy service to drug companies.[69 39] Consequently, payment data has functioned predominantly as a commodity used to monitor internal compliance with disclosure requirements and potentially inform marketing strategies targeting healthcare professionals.[70] Regarding data quality, insights gained through integrating payment data for the purposes of eurosfordocs.eu reinforce conclusions from UK research highlighting

For peer review only - http://bmjopen.bmj.com/site/about/guidelines.xhtml

that mapping payment distribution within a healthcare system requires forensic online checks of the recipient of each payment.[20]

It is well-documented that public regulation of payment disclosure can address key limitations of self-regulation, for example, by extending disclosure requirements to other industries or introducing compulsory disclosure.[4 5] Our study shows that its key advantage involves compulsory compliance, which contrasts sharply with many companies or industry trade groups following only the minimum requirements from the EFPIA Code.

Nevertheless, public regulation has not seen consistent progress in data quality and accessibility. Two of the countries investigated earlier, France and Portugal, still have limited data accessibility, and only Latvia demonstrated major improvement by introducing a centralised database.[4] In addition, public regulation often shares some of the key practical shortcomings of self-regulation. For example, without unique identifiers it is difficult to overcome the problem of duplicate entries in large databases.[20 21] or connect payment data to other databases, which prevents studying, for example, associations between payment and prescription patterns.[11] Further, the lack of payment itemisation prevents examining key aspects of marketing strategies, sometimes involving relatively "small payments", which have been associated with increased prescribing in the US.[71 72]

4.1. Limitations

This study has several limitations. The countries we classified as combining self-regulation and public regulation could be interpreted differently. While the Netherlands has been identified as a mixed approach by some,[4] others view it as a self-regulatory one.[5 6] Similarly, Spain could be interpreted as pursuing self-regulation, while Belgium – public regulation. However, the nature of interactions between public authorities and private actors in these countries is close to what was identified in the Netherlands, distinguishing them clearly from "pure" examples of either self- or public regulation.

Our measures of data accessibility could be expanded. For example, some disclosure databases are difficult to find, including web links to the Greek and Latvian databases published within news releases, with no permeant online location. Further, by emphasising on comparability between different types of data we did not measure "user-friendliness" directly as this would have required a more qualitative judgement.[4] Data quality could be scrutinised further by considering the comprehensiveness of disclosed information, for example, the types of disclosed donors, payments and recipients. However, given the difficulties involved in comparing the scope of disclosure in national regulations this would require a separate study.[4] Further, arriving at more nuanced overall evaluations of data accessibility and quality would require weighted measures based on qualitative insights from data users.

Our focus on the database level might obscure cross-company differences. For example, companies achieve widely varying consent rates from healthcare professionals, which suggests that similar differences can occur in relation to data quality and accessibility.[22] We did not quantify some company-level aspects of data accessibility (e.g. the share of image-based PDFs published by companies) and quality (e.g. the share of duplicate entries,

For peer review only - http://bmjopen.bmj.com/site/about/guidelines.xhtml

consistency of the use of donor or recipient categories, categories, missing data, mistakes in the data, such as negative values). Undertaking these calculations would have necessitated extensive forensic work[20] which was not possible within the resources available to this project. However, these problems are likely to be widespread and serious.[19-21]

More broadly, we only considered databases originating in the pharmaceutical industry and public authorities, excluding, for example, conflicts of interest regulation by professional bodies. Neither did we examine self-regulation in the broader life sciences sector, including manufacturers of generic medicines or medical devices.

4.2. Conclusions and policy recommendations

In Europe, drug company payments are typically disclosed in ways precluding public engagement with the data. We formulate suggestions for improvement (Table 5) also relevant for non-European countries, such as Japan, experiencing similar problems with payment data accessibility and quality.[73]

For peer review only

Table 5 How can the public authorities and the pharmaceutical industry improve the transparency of payment data?

	**Recommendations for improving accessibility of payment data**
1	Create national-level databases searchable for companies, recipients, and payment categories.
2	Make the databases in the csv or XLS format for further analysis.
3	Enable users to explore the data by allowing them to generate enable data summaries placing payments made or received in a broader context (e.g. payments made by other companies or received by the same or other recipient categories, such as medical specialty)
	**Recommendations for improving quality of payment data**
4	Publish unique identifiers for payment recipients shared by all companies and used consistently over time.
5	Introduce clear rules on the levels of aggregation for identifying recipients (e.g. clinic, ward, or hospital) to enhance the consistency of reporting.
6	Introduce categories of recipients to enable mapping the distribution of payments in the healthcare system. The categories relating to healthcare professionals could include a standardised list of medical specialties. The categories covering healthcare organisations could reflect their functions in the healthcare system as providers, commissioners, or professional organisations.
7	State clearly whether reported payments should include VAT or other taxes so that payment values from different companies can be compared reliably.
8	Publish each payment individually instead of aggregating them annually per recipient.
9	Publish payment descriptions so that the public can understand the activities they fund as well as their context. This requirement would follow the self-regulatory rules existing in relation to the disclosure of payments to patient organisations.
10	Enforce and publish detail of data quality checks: eliminate missing values, payments with the value of zero, and ensure that each recipient has a unique name and is reported at the same level of aggregation by all companies. Other data quality checks should involve cross-checking recipient name and address information to ensure consistency and avoid duplicate reporting.

For peer review only

Payment data accessibility could be enhanced very easily, with only minor revisions of the existing regulatory approaches. The top priority should involve creating centralised databases with expanded possibilities for data exploration allowing for contextualising payments made or received, potentially indicating conflicts of interest or undue influence.

Improvement of payment data quality would require more considerable regulatory changes. To allow meaningful engagement with the data it is vital to introducerecipient and donor identifiers. Similarly, recipient characteristics should be expanded to accommodate the ways in which the public engages with the healthcare system, including information on the types of healthcare organisations and professionals.[20] Further, to allow for meaningful interpretation payments should be reported together with information on products in relation to which they were made, following the example of Open Payments.[20] Similarly, drawing on EFPIA's self-regulation of payments to patient organisations, payments should be accompanied by meaningful descriptions covering their intended goals and funded activities (e.g.. specific conferences or projects).[74 75] No less important is granular disclosure allowing for capturing payments of different sizes. Importantly, to ensure the transparency and accountability of decision-making payment data should be connected – via recipient and product identifiers – to other relevant publicly available datasets including those detailing the patterns of prescription and procurement within publicly funded health systems. The integration and management of payment data should be supported by strong compliance mechanisms, including penalties for providing data of inadequate quality.

In countries following self-regulation, improving data accessibility and quality need to be combined with eliminating the possibility for non-disclosure by payment recipients as it translates into high levels of missing data.[22] This regulatory change could follow the interpretation of European data protection legislation (GDPR) by the Spanish Data Protection Authority stipulating that payment data does not constitute personal data.[22]

For peer review only

For peer review only - http://bmjopen.bmj.com/site/about/guidelines.xhtml

Contributors

PO is Senior Lecturer at the Department of Social and Policy Sciences, University of Bath. PO conceived and wrote the paper, collected and analysed the data. LM is a data scientist and the President of the Euros for Docs Association. LM created the eurosfordocs.eu database, analysed the data and contributed to writing. PAJ formerly presided the Euros for Docs Association. PAJ collaborated with LM on creating eurosfordocs.eu. PAJ conceived the paper and contributed to writing. SM is Associate Professor at the Department of Sociology, Lund University. SM conceptualized the paper and contributed to writing.

Acknowledgements

We would like to thank all pharmaceutical industry trade groups and public authorities which have responded to our request for information and were willing to explain the nature of local regulatory solutions. We thank, in particular, the ABPI's Disclosure UK Team for their continued interest in our research and openness in sharing details of their work.

We would like to express gratitude to all pharmaceutical industry trade groups and public authorities which have responded to our request for information and were willing to explain the nature of local regulatory solutions. We thank, in particular, the ABPI's Disclosure UK Team for their continued interest in our research as well as professionalism and openness in sharing the fine detail of their important work. We extend our thanks to colleagues who took the time to read drafts of this paper or offer linguistic assistance: Marcell Csanadi, Eniko Csanadi, Alice Fabbri, Kevin Jean, Fatma Korkmaz, Olga Loblova, Savvas Morris, Emily Rickard, Eszter Saghy, Christos Vasilakis, and Weronika Ozieranska.

Funding

This study (SM as PI and PO as Co-I) was supported by the grant 'Following the money: cross-national study of pharmaceutical industry payments to medical associations and patient organisations', awarded by The Swedish Research Council (VR), no. 2020-01822.

Details of the role of study sponsors

The funding body played no part in the design or conduct of this study

Statement of independence of researchers from funders

All researchers working on this study are independent from the funding body.

Competing interests

For peer review only

We have read and understood the BMJ Group policy on declaration of interests and declare the following interests:

PO's PhD student was supported by a grant from Sigma Pharmaceuticals, a UK pharmacy wholesaler and distributor (not a pharmaceutical company). The PhD work funded by Sigma Pharmaceuticals is unrelated to the subject of this paper.
LM and PAJ are members of Euros for Docs, a non-profit organization registered in France that seeks to promote transparency of drug company funding in the healthcare sector by making payment data accessible and complete across Europe.
PAJ is employed by Haute Autorité de Santé, the French independent health technology assessment organisation.
SM's partner is employed by PRA Health Sciences, a global Contract Research Organization whose costumers include many pharmaceutical companies.

Patient consent

None required.

Ethical approval

No ethical approval was needed. The ethical implications of this study article were approved via a peer ethics review process at the Department of Social and Policy Sciences, University of Bath in April 2016. This study did not require a full ethical approval as it relied on publicly available data aggregated at the organisational or country level.

Data sharing

We have included all relevant data as supplementary information forming part of this submission.

Exclusive license

The Corresponding Author has the right to grant on behalf of all authors and does grant on behalf of all authors, a worldwide licence to the Publishers and its licensees in perpetuity, in all forms, formats and media (whether known now or created in the future), to i) publish, reproduce, distribute, display and store the Contribution, ii) translate the Contribution into other languages, create adaptations, reprints, include within collections and create summaries, extracts and/or, abstracts of the Contribution, iii) create any other derivative work(s) based on the Contribution, iv) to exploit all subsidiary rights in the Contribution, v) the inclusion of electronic links from the Contribution to third party material where-ever it may be located; and, vi) licence any third party to do any or all of the above."

Transparency declaration

For peer review only

The guarantor (Piotr Ozieranski) affirms that this manuscript is an honest, accurate, and transparent account of the study being reported; that no important aspects of the study have been omitted; and that any discrepancies from the study as planned (and, if relevant, registered) have been explained.

For peer review only

References

1. Lo B, Field MJ. *Conflict of Interest in Medical Research, Education, and Practice* 2009
2. Lundh A, Lexchin J, Mintzes B, et al. Industry sponsorship and research outcome. *Cochrane Database of Systematic Reviews* 2017(2) doi: 10.1002/14651858.MR000033.pub3
3. Torgerson T, Wayant C, Cosgrove L, et al. Ten years later: a review of the US 2009 institute of medicine report on conflicts of interest and solutions for further reform. *BMJ Evidence-Based Medicine* 2020:bmjebm-2020-111503. doi: 10.1136/bmjebm-2020-111503
4. Fabbri A, Santos Al, Mezinska S, et al. Sunshine Policies and Murky Shadows in Europe: Disclosure of Pharmaceutical Industry Payments to Health Professionals in Nine European Countries. *International Journal of Health Policy and Management* 2018;7(6):504-09. doi: 10.15171/ijhpm.2018.20
5. Grundy Q, Habibi R, Shnier A, et al. Decoding disclosure: Comparing conflict of interest policy among the United States, France, and Australia. *Health Policy* 2018;122(5):509-18. doi: https://doi.org/10.1016/j.healthpol.2018.03.015
6. Mental Health Europe. Sunshine and transparency laws and regulations and codes across Europe 2017 [updated April 2017. Available from: https://mhe-sme.org/wp-content/uploads/2017/09/Mapping-of-Sunshine-Laws-in-Europe.pdf.
7. Santos A. The sun shines on Europe: transparency of financial relationships in the healthcare sector 2017 [10.
8. McDermott Will & Emery. Snapshot of Sunshine rules in EU countries for the pharmaceutical industry 2014 [updated June 2014. Available from: https://mcdermott-will-emery-2793.docs.contently.com/v/special-report-snapshot-of-sunshine-rules-in-eu-countries-for-the-pharmaceutical-industry1.
9. Moynihan R, Albarqouni L, Nangla C, et al. Financial ties between leaders of influential US professional medical associations and industry: Cross sectional study. *The BMJ* 2020;369 doi: 10.1136/bmj.m1505
10. Garstka ME, Monlezun D, DuCoin C, et al. The Sunshine Act and Surgeons: A Nation-Wide Analysis of Industry Payments to Physicians. *Journal of Surgical Research* 2019;233:41-49. doi: https://doi.org/10.1016/j.jss.2018.07.004
11. Yeh JS, Franklin JM, Avorn J, et al. Association of Industry Payments to Physicians With the Prescribing of Brand-name Statins in Massachusetts. *JAMA Internal Medicine* 2016;176(6):763-68. doi: 10.1001/jamainternmed.2016.1709
12. Mejia J, Mejia A, Pestilli F. Open data on industry payments to healthcare providers reveal potential hidden costs to the public. *Nature Communications* 2019;10(1):4314. doi: 10.1038/s41467-019-12317-z
13. Mitchell AP, Trivedi NU, Gennarelli RL, et al. Are financial payments from the pharmaceutical industry associated with physician prescribing? A systematic review. *Annals of Internal Medicine* 2020;Epub ahead of print doi: doi:10.7326/M20-5665 [published Online First: 24 Nov 2020]
14. Wayant C, Walters C, Zaaza Z, et al. Evaluation of Financial Conflicts of Interest Among Physician-Authors of American College of Rheumatology Clinical Practice Guidelines. *Arthritis & rheumatology* 2020;72(9):1427-34. doi: 10.1002/art.41224
15. Journal of Oral and Maxillofacial Surgery. *Journal of Oral and Maxillofacial Surgery*

For peer review only

16. El-Rayess H, Khamis AM, Haddad S, et al. Assessing concordance of financial conflicts of interest disclosures with payments' databases: a systematic survey of the health literature. *Journal of clinical epidemiology* 2020;127:19-28. doi: 10.1016/j.jclinepi.2020.06.040
17. Ornstein C, Weber T, Jones RG. We Found Over 700 Doctors Who Were Paid More Than a Million Dollars by Drug and Medical Device Companies, 2019.
18. Department of Justice - Office of Public Affairs. Medtronic to Pay Over $9.2 Million To Settle Allegations of Improper Payments to South Dakota Neurosurgeon, 2020.
19. Mulinari S, Ozieranski P. Disclosure of payments by pharmaceutical companies to healthcare professionals in the UK: analysis of the Association of the British Pharmaceutical Industry's Disclosure UK database, 2015 and 2016 cohorts. *BMJ Open* 2018;8(10):e023094. doi: 10.1136/bmjopen-2018-023094
20. Ozieranski P, Csanadi M, Rickard E, et al. Analysis of Pharmaceutical Industry Payments to UK Health Care Organizations in 2015. *JAMA Network Open* 2019;2(6):e196253-e53. doi: 10.1001/jamanetworkopen.2019.6253
21. Stoll M, Hubenschmid L, Koch C, et al. Voluntary disclosures of payments from pharmaceutical companies to healthcare professionals in Germany: a descriptive study of disclosures in 2015 and 2016. *BMJ Open* 2020;10(9):e037395. doi: 10.1136/bmjopen-2020-037395
22. Mulinari S, Martinon L, Jachiet P-A, et al. Pharmaceutical industry self-regulation and non-transparency: country and company level analysis of payments to healthcare professionals in seven European countries. *Health Policy* 2021;Forthcoming
23. Goupil B, Balusson F, Naudet F, et al. Association between gifts from pharmaceutical companies to French general practitioners and their drug prescribing patterns in 2016: retrospective study using the French Transparency in Healthcare and National Health Data System databases. *BMJ* 2019;367:l6015. doi: 10.1136/bmj.l6015
24. U.S. Attorney's Office - District of New Jersey. Novartis AG and Subsidiaries to Pay $345 Million to Resolve Foreign Corrupt Practices Act Cases 2020 [updated 25th June 2020. Available from: https://www.justice.gov/usao-nj/pr/novartis-ag-and-subsidiaries-pay-345-million-resolve-foreign-corrupt-practices-act-cases.
25. Securities and Exchange Commission. SEC Charges Eli Lilly and Company with FCPA Violations 2012 [updated 20th December 2012. Available from: https://www.sec.gov/news/press-release/2012-2012-273htm.
26. Moberly T. NHS joint working with industry is out of public sight. *BMJ* 2019;364:l1353. doi: 10.1136/bmj.l1353
27. Moberly T. CCGs fail to declare pharma funding. *BMJ* 2018;360:j5911. doi: 10.1136/bmj.j5911
28. EFPIA. EFPIA Code of practice 2019 [Available from: https://www.efpia.eu/media/554677/efpia_codes_a5_v3-2021_sm.pdf.
29. Eurosfordocs.eu. Metabase (access to the database will be provided to the BMJ Editors and Reviewers upon request - please email contact@eurosfordocs.fr to obtain the credentials) 2021 [Available from: https://eurosfordocs.eu/metabase/.
30. Anon. Hôpitaux : les CHU ont reçu 170 millions d'euros de laboratoires pharmaceutiques en 2018 2020 [updated 10th January 2020. Available from: https://www.lemonde.fr/sante/article/2020/01/10/hopitaux-les-chu-ont-recu-170-millions-d-euros-de-laboratoires-pharmaceutiques-en-2018_6025479_1651302.html

For peer review only

31. Anon. Procès du Mediator : « Le terreau des conflits d'intérêts à l'hôpital reste fertile » 2020 [updated 4th February 2020. Available from: https://www.lemonde.fr/idees/article/2020/02/04/le-terreau-des-conflits-d-interets-a-l-hopital-reste-fertile_6028302_3232.html.
32. Condomines A. Qu'est-ce que la plateforme EurosForDocs, qui recense les liens d'intérêts avec les industriels pharmaceutiques? 2021 [updated 4th January 2021. Available from: https://www.liberation.fr/checknews/2021/01/04/qu-est-ce-que-la-plateforme-eurosfordocs-qui-recense-les-liens-d-interets-avec-les-industriels-pharm_1809498.
33. Eaton L. Declarations of pharma funding to doctors are hit by covid-19 crisis. *BMJ* 2020;370:m2689. doi: 10.1136/bmj.m2689
34. ABPI. ABPI Code of Practice 2021 [Available from: https://www.pmcpa.org.uk/media/3292/2021-abpi-code-of-practice-technical-release.pdf.
35. Zetterqvist AV, Merlo J, Mulinari S. Complaints, Complainants, and Rulings Regarding Drug Promotion in the United Kingdom and Sweden 2004–2012: A Quantitative and Qualitative Study of Pharmaceutical Industry Self-Regulation. *PLOS Medicine* 2015;12(2):e1001785. doi: 10.1371/journal.pmed.1001785
36. Mulinari S, Davis C, Ozieranski P. Failure of Responsive Regulation? Pharmaceutical Marketing, Corporate Impression Management and Off-Label Promotion of Enzalutamide in Europe. *Journal of White Collar and Corporate Crime* 2020:2631309X097047. doi: 10.1177/2631309x20970477
37. Adlington K, Godlee F. Disclosure UK: transparency should no longer be an optional extra. *BMJ* 2016;354:i3730. doi: 10.1136/bmj.i3730
38. EFPIA. Report on Ethics & Compliance Activities 2020 [Available from: https://www.efpia.eu/media/554639/efpia-code-report-2019.pdf.
39. MediSpend. Global Compliance Platform 2021 [Available from: https://www.medispend.com/solutions/global-compliance/ accessed 9th January 2021 2021.
40. Agencia Española de Protección de Datos. INFORME 1438818/2016 2016 [
41. EFPIA. EFPIA HCP/HCO DISCLOSURE CODE. EFPIA CODE ON DISCLOSURE OF TRANSFERS OF VALUE FROM PHARMACEUTICAL COMPANIES TO HEALTHCARE PROFESSIONALS AND HEALTHCARE ORGANISATIONS 2014 [Available from: https://www.efpia.eu/media/25837/efpia-disclosure-code.pdf.
42. Ethical Committe for the Pharmaceutical Industry. Ethical rules for the pharmaceutical industry's donations and grants (Donation Code) N/A [Available from: https://www.enli.dk/media/49893/ethical-rules-on-donations-and-grants-version-20.pdf.
43. Amgen. Methodological Note. EFPIA & Local Industry Association Disclosure Codes 2020 [Available from: https://search.disclosureuk.org.uk/Public/GetMethodNoteFileForDownload?year=2019&pharmaCompanyId=29 accessed 25th February 2020.
44. betransparent.be. About us 2020 [Available from: https://www.betransparent.be/en/about-us/ accessed 18th January 2020.
45. Sunshine act 2016 [Available from: https://www.betransparent.be/wp-content/uploads/2017/01/Moniteur-Belge-2016-12-27-Belgisch-Staatsblad.pdf.

For peer review only

46. betransparent.be. Transparency in the interest of the patient 2020 [Available from: https://www.betransparent.be/en/about-the-transparency-register/ accessed 18th January 2020.
47. GSK. Engaging with healthcare professionals - Belgium 2020 [Available from: https://www.gsk.com/en-gb/responsibility/operating-responsibly/engaging-with-healthcare-professionals/europe/belgium/ accessed 18th January 2021.
48. Medicines Act 395/1987 1987 [Available from: https://www.fimea.fi/documents/160140/765540/18580_Laakelaki_englanniksi_paivitetty_5_2011.pdf.
49. Stichting Transparantieregister Zorg. Over het Transparantieregister 2020 [Available from: https://www.transparantieregister.nl/over-het-transparantieregister accessed 18th January 2020.
50. Stichting Transparantieregister Zorg. Hoe werken we? 2020 [Available from: https://www.transparantieregister.nl/over-het-transparantieregister/hoe-werken-we accessed 28th February 2021.
51. Stichting Transparantieregister Zorg. Veelgestelde vragen 2020 [Available from: https://www.transparantieregister.nl/veelgestelde-vragen.
52. Eurosfordocs.eu. Tech documentation 2021 [Available from: https://eurosfordocs.eu/on-the-tech-side/tech-documentation/ accessed 9th January 2021.
53. Anonymous. Pharmaceutical industry self-regulation and non-transparency: country and company level analysis of payments to healthcare professionals in seven European countries. *Under review in Health Policy* 2021
54. FSA. Transparenzkodex 2020 [updated 30th June 2020. Available from: https://www.fsa-pharma.de/de/mitteilungen/presse/archiv/transparenzveroffentlichungen-2019/.
55. FARMAINDUSTRIA. Las compañías farmacéuticas dedicaron el año pasado 483 millones a actividades de investigación y formación científica con organizaciones y profesionales sanitarios 2020 [updated 26th June 2020. Available from: https://www.farmaindustria.es/web/prensa/notas-de-prensa/2020/06/26/las-companias-farmaceuticas-dedicaron-el-ano-pasado-483-millones-a-actividades-de-investigacion-y-formacion-cientifica-con-organizaciones-y-profesionales-sanitarios/.
56. ScienceIndustries. Disclosure obligation since 2016 2020 [updated 22nd September 2020. Available from: https://www.scienceindustries.ch/en/article/13958/disclosure-obligation-since-2016.
57. IPHA. Email communication, 2020.
58. ABPI. Disclosure UK 2020 [Available from: https://www.abpi.org.uk/our-ethics/disclosure-uk/.
59. CurrencyConverter. CurrencyConverter 0.14.4 2020 [Available from: https://pypi.org/project/CurrencyConverter/ accessed 19th January 2021.
60. ABPI. Code of practice for the pharmaceutical industry 2019 [Available from: https://www.abpi.org.uk/media/6655/abpi-code-of-practice-2019.pdf.
61. ABPI. Email communication, 2021.
62. Csanádi M, Ozierański P, Löblová O, et al. Shedding light on the HTA consultancy market: Insights from Poland. *Health Policy* 2019;123(12):1237-43. doi: https://doi.org/10.1016/j.healthpol.2019.08.008

For peer review only

63. Ozierański P, Löblová O, Nicholls N, et al. Transparency in practice: Evidence from 'verification analyses' issued by the Polish Agency for Health Technology Assessment in 2012–2015. *Health Economics, Policy and Law* 2019;14(2):182-204. doi: 10.1017/S1744133117000342 [published Online First: 2018/01/08]
64. Bullement A, Taylor M, McMordie ST, et al. NICE, in Confidence: An Assessment of Redaction to Obscure Confidential Information in Single Technology Appraisals by the National Institute for Health and Care Excellence. *PharmacoEconomics* 2019;37(11):1383-90. doi: 10.1007/s40273-019-00818-0
65. Feldman AM, Mann DL. Restoring public trust in scientific research by reducing conflicts of interest. *The Journal of Clinical Investigation* 2019;129(10):3971-73. doi: 10.1172/JCI131448
66. EFPIA. Annual Review of 2011 and Outlook for 2012 2011 [Available from: https://www.efpia.eu/media/25261/annual-review-of-2011-and-outlook-for-2012.pdf.
67. EFPIA. Annual Review of 2011 and Outlook for 2012. 2011
68. European Commission. COMMUNICATION FROM THE COMMISSION TO THE EUROPEAN PARLIAMENT, THE COUNCIL, THE EUROPEAN ECONOMIC AND SOCIAL COMMITTEE AND THE COMMITTEE OF THE REGIONS Pharmaceutical Strategy for Europe 2020 [Available from: https://eur-lex.europa.eu/legal-content/EN/TXT/?uri=CELEX:52020DC0761.
69. IQVIA. Get to better business processes with embedded compliance 2021 [Available from: https://www.iqvia.com/solutions/integrated-global-compliance/commercial-compliance/ accessed 9th January 2021 2021.
70. Anand R, Collier D, Jiang Y, et al. Leveraging CMS Open Payments Data to Identify Channel Preferences and Gather Competitive Intelligence, Thereby Improving HCP Targeting ND [Available from: https://www.pmsa.org/jpmsa-vol05-article06.
71. DeJong C, Aguilar T, Tseng C-W, et al. Pharmaceutical Industry–Sponsored Meals and Physician Prescribing Patterns for Medicare Beneficiaries. *JAMA Internal Medicine* 2016;176(8):1114-22. doi: 10.1001/jamainternmed.2016.2765
72. Fleischman W, Agrawal S, King M, et al. Association between payments from manufacturers of pharmaceuticals to physicians and regional prescribing: cross sectional ecological study. *BMJ* 2016;354:i4189. doi: 10.1136/bmj.i4189
73. Ozaki A, Saito H, Senoo Y, et al. Overview and transparency of non-research payments to healthcare organizations and healthcare professionals from pharmaceutical companies in Japan: Analysis of payment data in 2016. *Health Policy* 2020;124(7):727-35. doi: https://doi.org/10.1016/j.healthpol.2020.03.011
74. Rickard E, Ozieranski P, Mulinari S. Evaluating the transparency of pharmaceutical company disclosure of payments to patient organisations in the UK. *Health Policy* 2019
75. Ozieranski P, Rickard E, Mulinari, Shai. Exposing drug industry funding of UK patient organisations. *BMJ* 2019;365:l1806. doi: 10.1136/bmj.l1806

For peer review only - http://bmjopen.bmj.com/site/about/guidelines.xhtml

Online Supplement 1

Online Supplement 1, Part 1: Questions to national pharmaceutical industry trade groups

Dear Sir or Madam

[Anonymised for the purposes of peer review]

I would be grateful if you could answer a few questions listed below regarding the way in which your organisation and its Members disclose transfers of value to healthcare professionals and organisations as well as patient organisations. Your answers would be very helpful in informing scholarly publications on which I am currently working that are seeking to present a full and accurate picture of transfers of value in Europe. Please let me know should you have any questions about this research.

[if relevant] In particular, your answers would help me expand on the information your Association provided in a recent EFPIA report and on its webpages, including here. Some of the matters covered in my questions have been touched upon in the sources of data referred to above, although incompletely. I would therefore be grateful if you could answer my questions so that I report correct and accurate information in any research outputs.

Thank you very much in anticipation for your valuable time in answering my questions.

I look forward to hearing from you.

Sincerely yours

[Anonymised]

For peer review only

I would be grateful if you could answer the following questions regarding your Association

1. What is the number of member companies in your Association?
2. How many companies which are not members of your Association follow its Code of Practice?
3. Please could you send me the most recent version of your Association's code of practice (if available, I would appreciate an English version of the document)?

Please could you answer the following questions regarding the platform and mechanisms of disclosure of transfers of value.

4. What is the platform for disclosing transfers of value to healthcare professionals and organisations (e.g. individual pharmaceutical company websites, a single database of transfers of value from all companies)?
5. If transfers of value are disclosed on individual pharmaceutical company websites, does your Association have a website providing links to these websites? If so, please could you provide me with a web link?
6. If transfers of value are disclosed on individual pharmaceutical company websites, has your Association considered creating a single database of transfers of value from all companies?
7. Do healthcare professionals in your country have to consent to the transfers of value being publicly disclosed; or is the disclosure of transfers of value to healthcare professionals mandatory (i.e. healthcare professionals are not asked to consent)? If the disclosure of transfers of value they received is mandatory, please could you state the regulation which makes them mandatory?
8. If healthcare professionals in your country have to consent to the transfers of value being publicly disclosed, does your Association have any expectations regarding the minimum HCP consent rate that should be achieved by companies signing up to its Code of Practice? If so, please could you specify this consent rate? Further, does your Association request companies achieving lower-than-expected healthcare professional consent rates to explain why this might be the case and identify possible ways of improving the consent rates?
9. If healthcare professionals in your country have to consent to the transfers of value being publicly disclosed, has your association considered implementing a simple summary disclosure rate statistic that is easily comparable between companies, such as the percent of the amount of transfers of value disclosed in the aggregate and/or the percent of healthcare professionals disclosed in the aggregate?
10. If healthcare professionals in your country have to consent to the transfers of value being publicly disclosed, does your association have any specific guidelines on how HCP disclosure rates could be improved by its Member Companies? If so, I would be grateful if you could share these guidelines with me?
11. If healthcare organisations have to consent to the transfers of value being publicly disclosed could you provide examples of reasons that would necessitate placing them in the aggregate disclosure?
12. Please could you state the threshold for the acceptable value of meals and drinks that was set by your association?
13. If some transfers of value need to be disclosed in a database run by a public institution, are there any transfers of value (e.g. research and development) that are still disclosed by pharmaceutical companies themselves. If so, I would be grateful if you could specify what these transfers of value are and why they are disclosed by pharmaceutical companies.
14. As far as your Association is aware, do companies disclosing their transfers of value use "unique country identifiers" recommended in the EFPIA Code of Practice, and not only recipient names or locations, to distinguish payment recipients? If so, are these identifiers

For peer review only

shared among different companies so that they can identify the same recipient using the same identifier?

15. Did members of your Association (or other companies following your Association's code of practice) disclose transfers of value to healthcare professionals and organisations in 2020 (i.e. covering payments made in the 2019 calendar year)?

If members of your Association (or other companies following your organisation's code of practice) disclosed transfers of value to healthcare professionals and organisations in 2020 (i.e. covering the 2019 calendar year) please could you answer the following questions.

16. How many pharmaceutical companies in total disclosed transfers of value to healthcare professionals and organisations made in 2019? How many of those companies were members of your Association?
17. Is the number of companies which did not disclose any transfers of value made in 2019 known? If so, please could you state this number? How many of those companies were members of your Association?
18. What was the overall share of healthcare professionals consenting to their transfers of value being disclosed in 2019?
19. What was the overall share of healthcare organisations consenting to their transfers of value being disclosed in 2019?
20. What was the total value of all non-research transfers of value to all healthcare professionals and organisations made in 2019?
21. What was the total value of all non-research transfers of value to all healthcare organisations made in 2019?
22. What was the value of all research-related (i.e. R&D) transfers of value to healthcare professionals or organisations made in 2019?
23. What share of the overall value of transfers of value made to healthcare professionals disclosed at the individual level in your country in 2019?
24. What share of the overall value of transfers of value made to healthcare organisations was disclosed at the individual level in your country in 2019?
25. What was the value of grants and donations made to healthcare organisations in 2019?
26. What was the value of fees for services and consultancy made to healthcare organisations in 2019?
27. What was the value of contributions to costs of events made to healthcare organisations in 2019?
28. What was the value of fees for services and consultancy made to healthcare professionals in 2019?
29. What was the value of contributions to costs of events made to healthcare professionals in 2019?

Finally, I would be grateful if you could answer the following questions regarding the public disclosure of transfers of value to patient organisations.

30. Please could you send me the most recent version of your Association's code of practice as it relates to working with patient organisations (if available, I would appreciate an English version of the document)?
31. Are there any companies that are not members of your Association which disclose transfers of value to patient organisations in line with the EFPIA Code of practice?
32. How many companies disclosed transfers of value to patient organisations made in 2019? How many of those companies were members of your Association?
33. What was the total value of all transfers of value to patient organisations made in 2019?

For peer review only

Online Supplement 1, Part 2. Questions to public or public-private authorities overseeing pharmaceutical industry payment disclosure

Dear Sir or Madam

[Anonymised for the purposes of peer review]

I would be grateful if you could answer a few questions listed below regarding the way in which your institution discloses payments made by drug companies to individuals and organisations within the healthcare system in your country. Your answers would be very helpful in informing scholarly publications on which I am currently working that are seeking to present a full and accurate picture of payments made by the pharmaceutical industry in Europe. Please let me know should you have any questions about this research.

Some of the matters covered in my questions have been touched upon on your website. However, I would be extremely grateful for your answers to avoid any misunderstandings which may be caused by language issues, particularly as they relate to complex regulatory matters.

Thank you very much in anticipation for your valuable time in answering my questions.

I look forward to hearing from you.

Sincerely yours

[Anonymised]

For peer review only

I would be grateful if you could answer the following questions regarding the nature of payments disclosed by [insert name of institution]

1. What industries are covered by the disclosure requirements (e.g. pharmaceutical, medical device, veterinary)?
2. Please could you briefly describe what is meant by each type of the industry covered by the disclosure?
3. What types of payment recipients are covered by the disclosure requirements (e.g. healthcare professionals, healthcare organisations, patient organisations)? Are these definitions consistent with the ones used in the EFPIA Code of Practice (https://www.efpia.eu/media/554677/efpia-code-2020.pdf) ?
4. Please could you briefly describe what is meant by each type of recipient (e.g. what is meant by the healthcare processional or healthcare organisation)? Are these definitions consistent with the ones used in the EFPIA Code of Practice?
5. What are the categories of payments covered by the disclosure managed by your institution (e.g. sponsorship of conference attendance, consultancy fees, research and development)? Are they the same one as those used in the EFPIA Code of Practice?
6. Please could you briefly describe what is meant by each type of payment (e.g. what is meant sponsorship of by conference attendance)? Are these definitions consistent with the ones used in the EFPIA Code of Practice?
7. If the definitions of payments or recipients used by your institution are not consistent with the ones used in the EFPIA Code of Practice, please could you explain why this is the case? In other words, why alternative definitions have been created for the reporting purposes of your institution as opposed to using the ones introduced by the EFPIA Code of Practice?
8. Are all payments to all payment recipients publicly disclosed by your organisations or are any payments or recipient types exempted from public disclosure?
9. Are payments reported by your institution reported on an individual basis (i.e. each payment has a separate database entry) or are they aggregated on a yearly basis (i.e. all payments of a certain type from a certain company are reported jointly)?
10. What are the responsibilities of companies making payments in relation to their disclosure?
11. What are the responsibilities of payment recipients in relation to their disclosure (e.g. do they need to disclose payments themselves or just verify disclosures made by companies making payments)?
12. Are payment disclosures managed by your institution reported based on calendar years or financial years? If payments are made based on financial years, please could you specify what are the start and end dates of a financial year in your country?
13. Are payment values made publicly available by your institution the same as those received by payment recipients (e.g. do they include the value of any taxes, such as VAT, paid by companies making the payments)?
14. If payments reported in by your institution are not reported consistently with or without relevant taxes (e.g. VAT), where might database users find information on approaches to tax reporting taken by each company making payments.
15. Do all industries covered by the payment disclosures managed by your institution report payments in exactly the same way (e.g. using the same definitions of payments, payment categories and recipients)?
16. Is the database of payments managed by your institution downloadable or not? Please could you explain why the decision has been made to make it downloadable (or not)?
17. Please could you send me the most recent copy of the regulation and/or policy which governs the disclosure of payments made your institution (if available, I would appreciate an English version of the document)?

For peer review only

Please could you answer the following questions regarding the disclosure requirements covered by your institution and the self-regulatory disclosure system overseen by the European Federation of Pharmaceutical Industries (EFPIA), as described in its Code of Practice?

18. Do pharmaceutical companies in your country have to disclose payments with your institution as well as in line with the disclosure system managed by EFPIA (i.e. do companies need to disclose payments to healthcare professionals and organisations with your institution as well as using disclosure reports comprising payments to healthcare professionals and organisations published on their individual websites)?
19. The EFPIA code of practice covers the category of payments related to research and development. Are these payments disclosed by your institution on a named basis or on drug company websites, in line with the EFPIA code of practice?

If payments made in 2019 calendar were reported year please could you answer the following questions regarding payments from <u>all industries</u> whose payments are managed by your institution?

20. How many companies reported payments in 2019?
21. What was the overall number of healthcare professionals receiving payments in 2019?
22. (If relevant) What was the overall number of healthcare organisations receiving payments in 2019?
23. (If relevant) What was the overall number of patient organisations receiving payments in 2019?
24. What was the overall value of payments made to healthcare professionals in 2019?
25. (If relevant) What was the overall value of payments made to healthcare organisations in 2019?
26. (If relevant) What was the overall value of payments made to patient organisations in 2019?

Please could you answer the following questions regarding only payments made by <u>pharmaceutical companies only</u> in the 2019 calendar year?

27. How many pharmaceutical companies reported payments in 2019?
28. Is the number of companies which did not disclose any payments made in 2019 known? If so, please could you state this number?
29. What was the overall number of healthcare professionals receiving payments from pharmaceutical companies in 2019?
30. (If relevant) What was the overall number of healthcare organisations receiving payments receiving payments from pharmaceutical companies in 2019?
31. (If relevant) What was the overall number of patient organisations receiving payments receiving payments from pharmaceutical companies in 2019?
32. What was the overall share of healthcare professionals consenting to their payments being disclosed in 2019?
33. (If relevant) What was the overall share of healthcare organisations consenting to their payments being disclosed in 2019?
34. (If relevant) What was the overall share of patient organisations consenting to their payments being disclosed in 2019?
35. What was the overall value of all payments made to healthcare professionals by pharmaceutical companies in 2019?
36. (If relevant) What was the overall value of payments made to healthcare organisations by pharmaceutical companies in 2019?

For peer review only

37. (If relevant) What was the overall value of payments made to patient organisations by pharmaceutical companies in 2019?
38. Please could you provide the value of each category of payments received by healthcare professionals from pharmaceutical companies in 2019?
39. (If relevant) Please could you provide overall the value of each category of payments received by healthcare organisations from pharmaceutical companies in 2019?
40. (If relevant) Please could you provide overall the value of each category of payments received by patient organisations from pharmaceutical companies in 2019?

Finally, I would be grateful if you could answer a few general questions regarding the direction nature of payments disclosed.

41. The obligatory disclosure of payments by a public body, such as your institution, seem be at odds the approach taken by many other European countries, which support a self-regulatory system managed by the pharmaceutical industry and allowing payment recipients not to have their payments disclosed based on the General Data Protection Regulation (GDPR). How does the system for payment disclosure managed by your institution addresses potential concerns regarding data privacy of payment recipients?
42. What are the advantages of the mandatory disclosure of payments, overseen by your institution, over a self-regulatory payment system based on the EFPIA Code of practice, existing in many other European countries?
43. What is the source of funding of the disclosure system managed by your institution (e.g. general taxation, health insurance, company fees)?
44. What is the yearly cost of maintaining the disclosure system managed by your institution?
45. How there been any examples of healthcare professionals not complying with the requirements of the disclosure system managed by your institution? If so, how were they addressed?
46. (if relevant) How there been any examples of healthcare organisations not complying with the requirements of the disclosure system managed by your institution? If so, how were they addressed?
47. (if relevant) How there been any examples of patient organisations not complying with the requirements of the disclosure system managed by your institution? If so, how were they addressed?
48. How there been any examples of pharmaceutical companies not complying with the requirements of the disclosure system managed by your institution? If so, how were they addressed?
49. Does your institution monitor who uses the disclosed payment data? If so, would you be able to say who the key types of users are?
50. What does your institution do to encourage the use of disclosed data (e.g. public campaigns)?

For peer review only

For peer review only

Online Supplement 2. List of approaches to regulating drug company payment disclosure in European countries

Country[1]	Regulation of drug company payment disclosure
AUSTRIA	Single disclosure system - only industry self-regulation and no state regulation
BOSNIA AND HERZEGOVINA	
BULGARIA	
CROATIA	
CYPRUS	
CZECH REPUBLIC	
GERMANY	
ICELAND	
IRELAND	
ITALY	
LUXEMBOURG	
NORTH MACEDONIA	
MALTA	
NORWAY	
POLAND	
RUSSIA	
SERBIA	
SLOVENIA	
SWEDEN	
SWITZERLAND	
UK	
UKRAINE	
FRANCE	Single disclosure system - only public regulation and no industry self-regulation
PORTUGAL	
TURKEY	
BELGIUM	Single disclosure system - a combination of industry self-regulation and public regulation or intervention
FINLAND	
THE NETHERLANDS	
SPAIN	
DENMARK	Two parallel systems - (1) Self regulation and (2) Public regulation covering certain payments, donors or recipients
ESTONIA	
GREECE	
HUNGARY	
LATVIA	
LITHUANIA	
ROMANIA	
SLOVAKIA	

For peer review only

Notes

[1] – European countries excluded from analysis: Albania, Andorra, Belarus, Lichtenstein, Monaco, Montenegro, San Marino, and Vatican City.

For peer review only

Online Supplement 3 Examples of deviations of data reporting formats from the EFPIA standard “disclosure template”

Part 1: EFPIA “disclosure template”

ANNEX A (binding)
Standardised disclosure template

ANNEX A - STANDARDISED DISCLOSURE TEMPLATE													Date of publication: ....
	Full Name (Art. 1.01)	**HCPs:** City of Principal Practice **HCOs:** city where registered (Art. 3)	Country of Principal Practice (Schedule 1)	Principal Practice Address (Art. 3)	Unique country identifier OPTIONAL (Art. 3)	Donations and Grants to HCOs (Art. 3.01.1.a)	Contribution to costs of Events (Art. 3.01.1.b & 3.01.2.a)			Fee for service and consultancy (Art. 3.01.1.c & 3.01.2.c)			**TOTAL** OPTIONAL
							Sponsorship agreements with HCOs / third parties appointed by HCOs to manage an Event	Registration Fees	Travel & Accommodation	Fees	Related expenses agreed in the fee for service or consultancy contract, including travel & accommodation relevant to the contract		
**HCPs**	**INDIVIDUAL NAMED DISCLOSURE - one line per HCP** (i.e. all transfers of value during a year for an individual HCP will be summed up: itemization should be available for the individual Recipient or public authorities' consultation only, as appropriate)												
	Dr A					N/A	N/A	Yearly amount	Yearly amount	Yearly amount	Yearly amount		
	Dr B					N/A	N/A	Yearly amount	Yearly amount	Yearly amount	Yearly amount		
	etc.					N/A	N/A	Yearly amount	Yearly amount	Yearly amount	Yearly amount		
	OTHER, NOT INCLUDED ABOVE - where information cannot be disclosed on an individual basis for legal reasons												
	**Aggregate amount attributable to transfers of value to such Recipients** - Art. 3.02					N/A	N/A	Aggregate HCPs	Aggregate HCPs	Aggregate HCPs	Aggregate HCPs		Optional
	**Number of Recipients in aggregate disclosure** - Art. 3.02					N/A	N/A	number	number	number	number		Optional
	% of the number of Recipients included in the aggreate disclosure in the total number, by category, of Recipients disclosed - Art. 3.02					N/A	N/A	%	%	%	%		N/A
**HCOs**	**INDIVIDUAL NAMED DISCLOSURE - one line per HCO** (i.e. all transfers of value during a year for an individual HCO will be summed up: itemization should be available for the individual Recipient or public authorities' consultation only, as appropriate)												
	HCO 1					Yearly amount	Yearly amount	Yearly amount	Yearly amount	Yearly amount	Yearly amount		Optional
	HCO 2					Yearly amount	Yearly amount	Yearly amount	Yearly amount	Yearly amount	Yearly amount		Optional
	etc.					Yearly amount	Yearly amount	Yearly amount	Yearly amount	Yearly amount	Yearly amount		Optional
	OTHER, NOT INCLUDED ABOVE - where information cannot be disclosed on an individual basis for legal reasons												
	**Aggregate amount attributable to transfers of value to such Recipients** - Art. 3.02					Aggregate HCOs	Aggregate HCOs	Aggregate HCOs	Aggregate HCOs	Aggregate HCOs	Aggregate HCOs		Optional
	**Number of Recipients in aggregate disclosure** - Art. 3.02					number	number	number	number	number	number		Optional
	% of the number of Recipients included in the aggreate disclosure in the total number, by category, of Recipients disclosed - Art. 3.02					%	%	%	%	%	%		N/A

**R&D**	**AGGREGATE DISCLOSURE**		
	Transfers of Value re Research & Development as defined - *Article 3.04 and Schedule 1*	TOTAL AMOUNT	OPTIONAL

latest update: 27 June 2019

Note
The screenshot was taken from version the EFPIA Code which was in force the time of writing [1].

Part 2: Examples of data reporting not following the EFPIA “disclosure template”

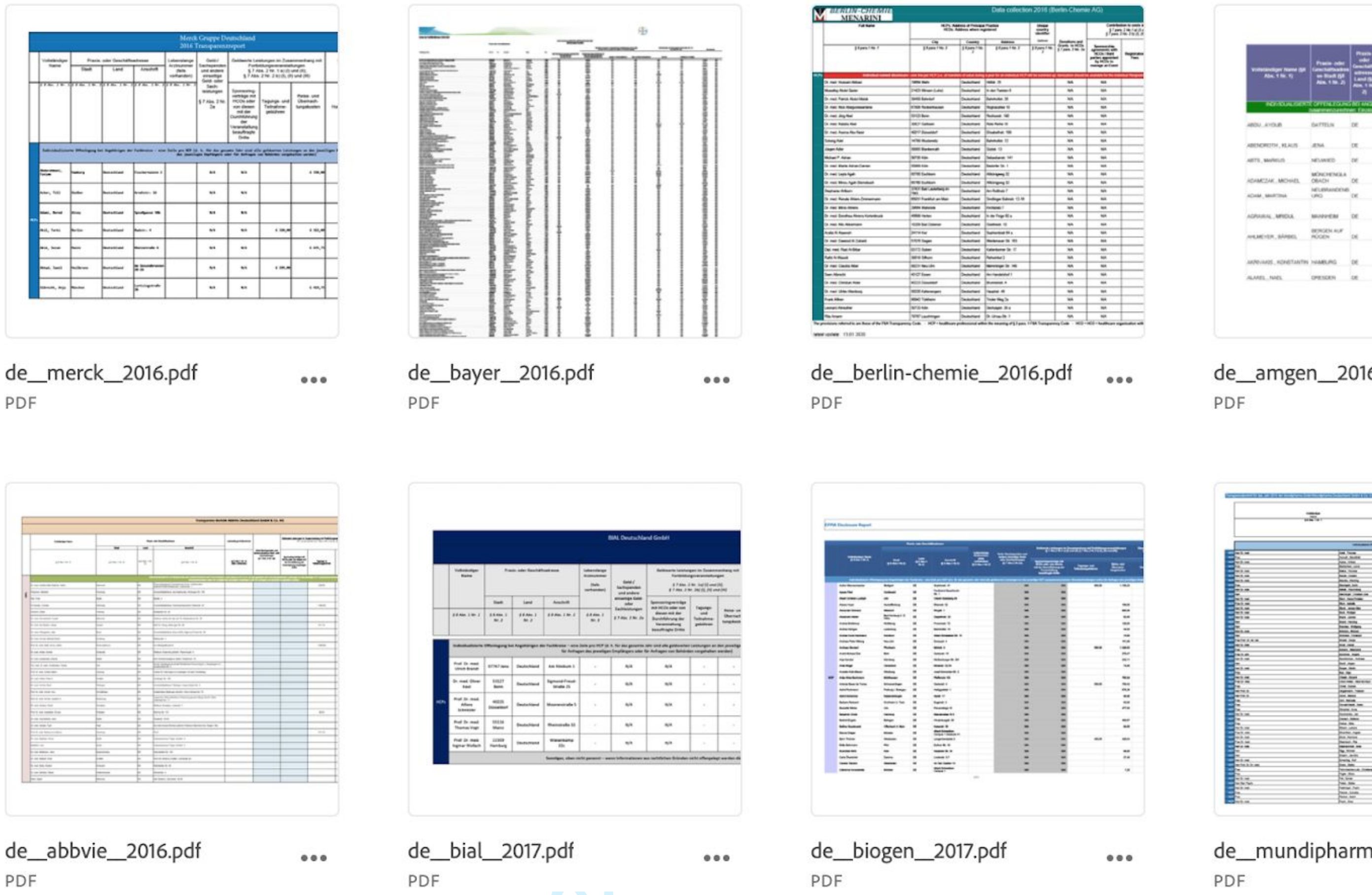

Note

The screenshots were taken as part of the data curation process involved in creating the eurosfordocs.eu database.[2]

References

1. EFPIA. EFPIA Code of practice 2019 [Available from: https://www.efpia.eu/media/554677/efpia_codes_a5_v3-2021_sm.pdf.
2. Eurosfordocs.eu. Tech documentation, 2021 [Available from: eurosfordocs.eu/on-the-tech-side/tech-documentation/ accessed 4th January 2021.

Online Supplement 4: Approaches to data presentation decreasing the accessibility of payment disclosures

Part A: Techniques used when publishing payment data on individual company websites

1. Publish data as PDF documents (and not csv or XLS files) to make analysis difficult (example: most companies throughout Europe, except where the pharmaceutical industry trade groups created centralised databases).
2. Make the PDF document available only in an online viewer rather than as a separate file to prevent it from being downloaded for analysis (example: Menarini Switzerland).
3. Publish PDF documents consisting of images (and not text) to prevent any searches within the data (e.g. for recipient names) (example: Pfizer Sweden).
4. Reduce the resolution of image-based PDF documents to make them *almost* unreadable without constantly zooming in and out (example: Novo-Nordisk Spain).
5. Create PDF documents with repeated table headers occupying most of each page in the disclosure report. In some cases, if the content is image-based, the resulting PDF can exceed 1800 pages, and over 350 MB, which discourages users from opening or downloading it (example: Novartis Italy).
6. Require users to follow a lengthy process of accepting the "Terms of use" of the disclosed information to discourage engagement with the data (example: Pfizer Spain).

Note: Different techniques can be combined. For example, the disclosure report can be made available only in an online viewer (2), with each page published as an image (3) and in a low resolution (4) (example: Roche Italy).

Part B: Techniques used when publishing payment data in centralised databases.

1. Require users to follow a lengthy process of accepting the "terms of use" of the disclosed information to discourage engaging with the data (example: the Czech Transparentní spolupráce database).
2. Enable searching only for specific recipients, without the possibility of searching for companies or recipient categories (e.g. medical specialties) (the Czech Transparentní spolupráce database).
3. Make searches conditional on obtaining recipient ID numbers from another website (example: the Czech Transparentní spolupráce database)
4. Do not include the possibility of downloading the database as a single file to prevent analysis (Examples: the Irish Transfer of Value database – and all centralised industry platforms except Disclosure UK)

For peer review only

Online supplement 5. Eurosfordocs.eu – database summary (2017-2019)[1]

Country	Disclosure reports	Successfully extracted (parsed) disclosure reports	Parse ratio	Companies associated with parsed disclosure reports	Number of payments to healthcare professionals and organisations	Value of payments (€)[2]
UK	1[3]	1	100%	141	164,112	1,771,785,871
Germany	112	89	79%	32	103,477	1,524,231,568
Spain	60	48	80%	16	370,444	959,704,223
Italy	60	57	95%	19	143,244	954,063,974
Switzerland	138	117	85%	41	36,503	471,638,889
Sweden	184	168	91%	68	15,434	249,913,018
Ireland	1[3]	1	100%	46	18,312	97,259,959
Total	556	481		160	851,526	6,028,597,501

Notes.

[1] – All data is accurate as of January 2021. Eurosfordocs.eu is updated regularly to reflect occasional changes in disclosure reports published by drug companies.

[2] – All payment values in non-euro currencies were converted to euros based the exchange rate obtained from the CurrencyConverter [1] a Python library for exchange rates.

[3] – The UK and Ireland are the only countries reported in the table in which all drug company payments are included in a single database. In all other countries, disclosure reports are published on individual websites for each company.

References

1. CurrencyConverter. CurrencyConverter 0.14.4 2020 [Available from: https://pypi.org/project/CurrencyConverter/ accessed 19th January 2021.

For peer review only - http://bmjopen.bmj.com/site/about/guidelines.xhtml

BMJ Open

Accessibility and quality of drug company disclosures of payments to healthcare professionals and organisations in 37 countries: A European policy review

Journal:	*BMJ Open*
Manuscript ID	bmjopen-2021-053138.R1
Article Type:	Original research
Date Submitted by the Author:	02-Sep-2021
Complete List of Authors:	Ozieranski, Piotr; University of Bath, Department of Social and Policy Sciences<br>Martinon, Luc; Euros for Docs<br>Jachiet, Pierre-Alain; Euros for Docs, Department of Social and Policy Sciences<br>Mulinari, Shai; Lunds Universitet, Sociology
**Primary Subject Heading**:	Health policy
Secondary Subject Heading:	Ethics
Keywords:	ETHICS (see Medical Ethics), Health policy < HEALTH SERVICES ADMINISTRATION & MANAGEMENT, Protocols & guidelines < HEALTH SERVICES ADMINISTRATION & MANAGEMENT

SCHOLARONE™
Manuscripts

I, the Submitting Author has the right to grant and does grant on behalf of all authors of the Work (as defined in the below author licence), an exclusive licence and/or a non-exclusive licence for contributions from authors who are: i) UK Crown employees; ii) where BMJ has agreed a CC-BY licence shall apply, and/or iii) in accordance with the terms applicable for US Federal Government officers or employees acting as part of their official duties; on a worldwide, perpetual, irrevocable, royalty-free basis to BMJ Publishing Group Ltd ("BMJ") its licensees and where the relevant Journal is co-owned by BMJ to the co-owners of the Journal, to publish the Work in this journal and any other BMJ products and to exploit all rights, as set out in our licence.

The Submitting Author accepts and understands that any supply made under these terms is made by BMJ to the Submitting Author unless you are acting as an employee on behalf of your employer or a postgraduate student of an affiliated institution which is paying any applicable article publishing charge ("APC") for Open Access articles. Where the Submitting Author wishes to make the Work available on an Open Access basis (and intends to pay the relevant APC), the terms of reuse of such Open Access shall be governed by a Creative Commons licence – details of these licences and which Creative Commons licence will apply to this Work are set out in our licence referred to above.

Other than as permitted in any relevant BMJ Author's Self Archiving Policies, I confirm this Work has not been accepted for publication elsewhere, is not being considered for publication elsewhere and does not duplicate material already published. I confirm all authors consent to publication of this Work and authorise the granting of this licence.

For peer review only

Accessibility and quality of drug company disclosures of payments to healthcare professionals and organisations in 37 countries: A European policy review

Piotr Ozieranski [1]
Luc Martinon[2]
Pierre-Alain Jachiet[2]
Shai Mulinari [3]

[1] Department of Social and Policy Sciences, University of Bath
[2] Euros for Docs
[3] Department of Sociology, Lund University

Correspondence to:
Piotr Ozieranski
Department of Social and Policy Sciences
University of Bath
BA2 7AY
Email: p.ozieranski@bath.ac.uk
Phone: +44 (0) 1225 384204

Key words: pharmaceutical industry, self-regulation, public regulation, transparency, financial disclosure, conflicts of interest

Wordcount: 5388

For peer review only

Abstract

Objectives: To examine the accessibility and quality of drug company payment data in Europe.

Design: Comparative policy review of payment data in countries with different regulatory approaches to disclosure.

Setting: 37 European countries.

Participants: European Federation of Pharmaceutical Industries and Associations, its trade group and their drug company members; eurosfordocs.eu, an independent database integrating payments disclosed by companies and trade groups; regulatory bodies overseeing payment disclosure.

Main outcome measures: Regulatory approaches to disclosure (self-regulation, public regulation, combination of the two); data accessibility (format, structure, searchability, customisable summary statistics, downloadability) and quality (spectrum of disclosed characteristics, payment aggregation, inclusion of taxes, recipient or donor identifiers).

Results: Of 30 countries with self-regulation five had centralised databases, with Disclosure UK displaying the highest accessibility and quality. In 23 of the remaining countries with self-regulation and available data, disclosures were published as PDFs on individual company websites, preventing the public from understanding payment patterns. Eurosfordocs.eu had greater accessibility than any industry-run database, but the match between of the value of payments integrated in eurosfordocs.eu and summarised separately by industry in seven countries ranged between 56%-100% depending on country. Eurosfordocs.eu shared quality shortcomings with the underlying industry data, including ambiguities in identifying payments and their recipients. Public regulation was found in 15 countries, used either alone (3), in combination (4) or in parallel with (8) self-regulation. Of these countries, 13 established centralised databases with widely ranging accessibility and quality and sharing some shortcomings with the industry-run databases. The French database, Transparence Santé, had the highest accessibility and quality, exceeding that of Disclosure UK.

Conclusions: The accessibility and quality of payment data disclosed in European countries are typically low, hindering investigation of financial conflicts of interest. Some improvements are straightforward but reaching the standards characterising the widely researched US Open Payments database requires major regulatory change.

Strengths and limitations

- We investigate the quality and accessibility of drug company payment disclosure data in 37 European countries.
- We use a set of measures relevant for countries with industry self-regulation, public regulation, and a combination of the two.
- We present our results as a “heat map” showing the least and most problematic aspects of payment data accessibility and quality.

For peer review only - http://bmjopen.bmj.com/site/about/guidelines.xhtml

- One key limitation is that that we did not quantify some aspects of the accessibility and quality of payment data.

For peer review only

For peer review only - http://bmjopen.bmj.com/site/about/guidelines.xhtml

1. Introduction

Financial conflicts of interest (FCOIs) can bias healthcare research, practice, education, and policy.[1-3] The last decade has seen a global trend towards addressing concerns about FCOIs by publishing drug company payments to the healthcare sector.[4-8] It is best exemplified by the US Sunshine Act, establishing Open Payments, a database triggering extensive research on payment distribution,[9 10] and its links with drug prescription[11] and cost.[12 13] Open Payments increases transparency of FCOIs by enabling cross-checking information collected by professional organisations,[14] conference organisers,[15] and scientific journals.[16] It also aids identifying corruption by highlighting unusual payment patterns.[17 18]

Unlike the US, in most European countries drug company payments are disclosed via industry self-regulation.[4 6] In Europe, the prevalent form of self-regulation draws on the Code of Practice of the European Federation of Pharmaceutical Industries and Associations (EFPIA), with its minimum requirements transposed into the codes of EFPIA's national trade group members.[19] Self-regulation allows the industry to develop, implement, and oversee the rules of payment disclosure.[4 20] Compared to the US Sunshine Act, one key shortcoming of self-regulation, resulting from the industry's interpretation of European privacy laws, is making company disclosures conditional on consent granted by payment recipients.[21 22] Other problems include broader, and therefore difficult to interpret, payment categories (grants and donations, contributions to costs of events, fees for service and consultancy),[22] which are also fewer than in the US, excluding royalties, ownership and investments. Additionally, research payments are only disclosed as lump sums per company without named recipients.[5 23] One advantage of self-regulation is a greater scope of covered healthcare professionals, including not only physicians but also nurses (to be included in the US starting from 2022[24]), pharmacists, and others. [5 21] Further, self-regulation includes, like in the US, hospital recipients of payments but also general practice surgeries, professional associations, and other healthcare organisations.[5 23]

Only few European countries, including France, Portugal and Latvia, use government regulation, principally legislation, to impose disclosure requirements for donors and recipients, including mandatory disclosure.[4 6] Finally, one country, the Netherlands, has been identified as using a combination of self- and public regulation, with the disclosure regulations developed with government's input, lacking a legal basis and enforced via self-regulation.[4]

The scrutiny of European payment data has been limited, except for case studies of payment distribution in the UK,[21 23] Germany,[25] and Ireland,[26] and a comparative analysis of payments shares not disclosed by recipients in the UK, Germany, Sweden, Switzerland, Italy, Ireland, and Spain.[27] However, France is the sole country where relationships between payments and prescribing have been investigated.[28] Similarly, the potential for detecting organisational-level FCOIs is unrealised, with only two studies examining discrepancies in payments reported separately by companies and some healthcare providers[29] and commissioners[30] in England. Further, corrupt relationships identified via official investigations pertaining to Greece,[31] Poland and Russia[32] might have been revealed earlier by examining payment patterns, following the US' example.[17 18] Therefore, the evidence

For peer review only - http://bmjopen.bmj.com/site/about/guidelines.xhtml

base for any policy reform is thin, leaving the industry as the only stakeholder likely to have in-depth understanding of payment data, particularly in countries with self-regulation.

The likely reasons behind the scant disclosure research are the low accessibility and quality of payment data. Regarding accessibility, a study of European disclosure approaches has found that of six countries with self-regulation five lacked centralised payment databases.[4] In one of these countries, Germany, the dispersal of disclosures on drug company websites was a major obstacle in data analysis.[19 25 27] A recent remedial initiative by activist data scientists has involved creating a database called eurosfordocs.eu. Inspired by a similar German project,[25] eurosfordocs.eu integrates data disclosed separately by many companies in countries with self-regulation.[27 33] Contrastingly, of the four countries identified as having government regulation or combining it with self-regulation three had databases integrating payments reported by all companies.[4]

A related aspect of low accessibility both in countries with self-regulation and government regulation is poor user interface.[4] Of the six studied countries with self-regulation only Disclosure UK, the database run by the Association of the British Pharmaceutical Industry (ABPI), was judged as user friendly.[4] However, of the three databases in countries using government regulation or combining it with self-regulation the Dutch and Portuguese databases were described as “partially” user friendly, while the French was deemed “not” user friendly.[4] Challenges in the interface of the French database were only addressed by the independent data platform eurosfordocs.fr, stimulating journalistic investigations into FCOIs.[34-36]

The second problem, payment data quality, has only been examined in countries with self-regulation. For example, analyses of Disclosure UK revealed inconsistencies in reporting of payment values and recipients,[21 23] compounded by the absence of unique recipient identifiers.[37] Similar shortcomings, including duplicate entries, were found in Germany,[25] indicating that they might characterise self-regulation more broadly.

Therefore, important gaps exist in our understanding of the accessibility and quality of European payment data. First, ongoing debates on the introduction of public regulation in some countries[5] suggest that the only comprehensive European regulatory overview[6] might have missed key regulatory developments, potentially with implications for data accessibility and quality.

Second, the implementation of the requirements of the EFPIA Code[19] has not been fully scrutinised. For example, although some trade groups will only meet the minimum standards (e.g. by expecting companies to publish data on their websites), others might exceed them (e.g. by creating centralised databases).[4 27] The need for establishing a comprehensive pattern of compliance is underscored by findings from Sweden and the UK suggesting failure of self-regulation of drug marketing to meet some of its own key promises.[20 38]

Third, regulatory approaches in many European countries have escaped scrutiny,[4] making it unclear whether payment data reported in these countries shares the strengths and weaknesses identified elsewhere. Consequently, although some aspects of government

For peer review only

regulation, such as a greater scope of covered industries, have been demonstrated as superior to self-regulation,[4 6] it remains uncertain whether this is reflected by payment data accessibility or quality.[4]

Finally, the to-date evaluative criteria need refinement, as some, such as "user friendliness" have attracted a contrasting appraisal of the same disclosure database by different expert commentators.[4 22]

We have two objectives. First, to identify regulatory approaches to payment disclosure in Europe. Second, to examine the accessibility and quality of payment data disclosed in countries with different approaches to disclosure.

2. Methods

2.1. Data collection

2.1.1. Identification of regulatory approaches

To identify regulatory approaches to payment disclosure in Europe, PO and LM identified available peer-reviewed English-language research on the regulation of drug company payment disclosure. We searched Scopus using the terms "Sunshine Act", "Open Payments", as well as "European Federation of Pharmaceutical Industries and Associations" and "EFPIA" combined with "disclosure". We applied the same terms in the Google search engine to identify "grey literature", including non-peer reviewed reports.

Subsequently, PO and LM conducted iterative searches on websites dedicated to industry payment disclosure, including EFPIA's website and its national trade group members' websites. We also examined the country profiles published by MediSpend[39] and the websites of four major companies with presence across Europe (Amgen, GSK, Merck Serono, and Bayer) providing access to company disclosure methodologies which reflect local regulatory requirements. Finally, we considered the websites of public or multistakeholder bodies which the previous steps identified as involved in overseeing payment disclosure.

Finally, PO surveyed industry trade groups and public or multistakeholder bodies overseeing payment disclosure (Online Supplement 1). The first round of standardised questions was emailed in mid-November 2020, followed up by reminder messages in late December 2020, asking recipients to provide answers by the end of the 1st week of January 2021. Of 34 approached pharmaceutical trade groups 17 replied. Of those, 14 answered at least some of the questions, while the remaining ones sent holding messages. Of 13 approached public or public or multistakeholder bodies ten replied. Of those, six answered at least some of the questions, three sent holding messages, and one redirected us to another institution (Online Supplement 2).

For peer review only

2.1.2. Data on accessibility and quality of payment disclosures

First, in countries with self-regulation, we considered industry codes, reports, press releases, trade group websites, and industry-run databases. Second, LM and PAJ recorded their observations regarding the format and structure of payment data when designing scripts for scraping company and trade group websites to be integrated in eurosfordocs.eu.[27] Third, in countries with disclosure overseen by public or multistakeholder bodies the data included relevant legislation, the websites of bodies managing payment disclosure, and disclosure databases. Fourth, in both countries with self-regulation and public regulation we considered responses from our stakeholder survey. Finally, in countries with self-regulation and covered by eurosfordocs.eu we collected – for verification purposes – national-level summary statistics published by EFPIA, industry trade groups, and survey responses from the trade groups.

2.2. Data analysis

2.2.1. Content analysis

Most of the source material was available in English. If this was not the case, we used Google Translate and Deepl.com, clarifying any linguistic issues by cross-checking with other online sources and consulting with relevant national bodies and colleagues with language expertise.

We coded the regulatory approaches deductively building on an earlier categorisation which distinguished countries with self-regulation, government regulation, and a combination of the two.[4] We modified it by considering new regulatory developments, such as the 2016 decision by the Spanish Data Protection Agency[40] making disclosure by healthcare professionals compulsory without new government regulation.[27] Therefore, we replaced "government regulation" with "public regulation", comprising "government regulation", i.e. legislation relating directly to payment disclosure, and "regulatory intervention", i.e. decisions by data protection agencies clarifying the rules of payment disclosure based on other existing legislation.

Deductive codes relating to data accessibility and quality were developed using earlier research.[4 5 23] Inductive coding was applied to the types of disclosed information and company techniques of decreasing data accessibility, which were identified when integrating industry data within eurosfordocs.eu

The data was coded by PO and results were validated by team discussions, resolving any differences by agreement. In analysing industry-self regulation, we set the characteristics of disclosed data against recommendations from the EFPIA Code. Similar comparison was not necessary in relation public regulation as it does not introduce any optionality.

2.2.2. Descriptive statistical analysis

For peer review only - http://bmjopen.bmj.com/site/about/guidelines.xhtml

For peer review only

As eurosfordocs.eu involved data extraction using disclosures published by individual companies and industry trade groups, we estimated the match between the database and the underlying data by comparing the value of payments calculated in specific countries using eurosfordocs.eu with national-level summaries obtained from industry sources.

2.2.3. Outcome measures

We had one primary outcome measure identifying the regulatory approaches to payment disclosure in each country – self-regulation, public regulation and a combination of the two. As we identified both self-regulation and public regulation in some countries, we noted the number of regulatory approaches in each country – single (only self-regulation, public regulation, or a combination of the two) or two (self-regulation and public regulation used in parallel).

In countries with self-regulation, we recorded whether it was based on the EFPIA Code, including shared payment, donor, and recipient categories, or involved a distinct national industry code. For countries following the EFPIA Code, we specified whether trade groups were obliged to do so as EFPIA members or did this voluntarily as non-members.

Considering countries with public regulation, we distinguished those using government regulation, regulatory intervention, or both. In countries with government regulation, we distinguished those introducing bespoke legislation focusing on payment disclosure or incorporating new provisions into existing pharmaceutical or medical device legislation. In countries where public-and self-regulation were used in parallel, we recorded whether any overlap existed between the donors, recipients and payments covered by each approach.

In countries combining self-and public regulation, we denoted the form of both self-regulation and public regulation and how they were integrated.

The measures of accessibility and quality reflected the heterogeneity of payment data presentation. The basic measure of accessibility applied in all countries was whether it was disclosed on a centralised database or multiple websites. In addition, for countries with centralised databases, we created a "heat map" aiding data synthesis and interpretation (Table 1).

For peer review only

Table 1 Heat map of measures of accessibility and quality of payment databases.

		**Measures of payment data accessibility**		
		**Higher accessibility**	**?--------------?**	**Lower accessibility**
Database format	How is the database published (i.e. PDF, XLS, CSV, webpage)?	Webpage, XLS or CSV	Readable PDFs	Image-based PDFs
Database structure	Does the data from all companies follow a single template consistently?	Yes	N/A	No
Database searchability	Can the database be searched? If so, can database searches be carried out without data users providing any additional information?	Yes	Database searchable but additional information needed for searches	No
Customisable summary statistics	Does the database offer users the possibility of generating real-time, dynamic data summaries based on selected database characteristics?	Yes	N/A	No
Downloadability	Can the database be downloaded (e.g. as a single CSV or XLS file) for further analysis?	Yes	N/A	No
		**Measures of payment data quality**		
		**Higher quality**	**?--------------?**	**Lower quality**
Spectrum of disclosed characteristics	What characteristics are included in relation to donors, recipients, and payments?	All characteristics from the EFPIA disclosure template covered as well as some additional ones	All characteristics from the EFPIA disclosure template covered	At least some characteristics from the EFPIA disclosure template not covered, including instances where some additional characteristics are provided
Aggregation of payments	Are payments itemised (i.e. all payments have separate entries) or are they aggregated on an annual basis (e.g. per recipient and/or payment category)?	All payments itemised	Some payments itemised, other s aggregated	All payments aggregated
Inclusion of taxes	Is it clear whether payments are reported inclusive or exclusive of any taxes, such as VAT?	Single rule for all companies and payments	No single rule, each company sets its own rules for VAT reporting which are published separately from payment disclosures[1]	Rules around tax reporting are unclear
Unique identifiers	Do reported donors (drug companies) or recipients (healthcare professionals or organisations) have unique identifiers?	All donors and recipients	Some donors or recipients	No unique identifiers

Notes

[1] – The EFPIA Code stipulates that companies must publish documents, called "methodological notes", which should explain their approach to reporting VAT and other taxes. Companies publish these documents separately from payment disclosures but consulting them is necessary to understand, compare, and aggregate payment values.

For peer review only - http://bmjopen.bmj.com/site/about/guidelines.xhtml

On top of the measures included in Table 1 we had one additional measure of quality for eurosfordocs.eu as a database derived from payment disclosures published by drug companies and industry trade groups. We estimated the comprehensiveness of data extraction by comparing the value of payments available in eurosfordocs.eu with those reported separately in national-level industry data summaries. We set three arbitrary levels of match – exact (no difference between eurosfordocs.eu and summary industry data), close (difference between eursofrdocs.eu and industry data worth less than 10% of summary industry data) and low (difference exceeding 10% of summary industry data).

Finally, in countries with self-regulation but without centralised databases we examined whether industry trade groups created gateways leading to disclosure documents, as recommended by EFPIA.[19] To illustrate challenges in data accessibility we also generated lists of examples of, first, deviations from the EFPIA-recommended data presentation format ("EFPIA disclosure template"[19]); and, second, the ways of presenting data which decreased its accessibility.

2.3. Ethics

This study did not require a full ethics approval as no individual payment data was processed. Its ethical implications were approved via a peer ethics review process at the Department of Social and Policy Sciences, University of Bath in April 2016.

2.4. Patient and public involvement

We did not involve patient groups or the public. Our policy recommendations seek to increase public engagement with payment data by enhancing its accessibility and quality.

3. Results

We first map the regulatory approaches to payment disclosure in Europe. We then examine the accessibility and quality of payment data published by pharmaceutical companies and trade groups in countries with self-regulation. Subsequently, we focus on industry data in the subset of countries with self-regulation and covered by eurosfordocs.eu. Finally, we analyse payment data in countries with public regulation or combining public regulation with industry self-regulation.

3.1. Mapping European regulatory approaches to payment disclosure

Before analysing the accessibility and quality of industry payment data we must describe how it is disclosed in each European country (Box 1).

For peer review only - http://bmjopen.bmj.com/site/about/guidelines.xhtml

Box 1. Approaches to regulating payment disclosure in European countries

Country[1]	Regulatory approaches to payment disclosure: Self-regulation	Public regulation	Combination of self- and public regulation
AUSTRIA	✓		
BOSNIA AND HERZEGOVINA	✓		
BULGARIA	✓		
CROATIA	✓		
CYPRUS	✓		
CZECH REPUBLIC	✓		
GERMANY	✓		
ICELAND	✓		
IRELAND	✓		
ITALY	✓		
LUXEMBOURG	✓		
NORTH MACEDONIA	✓		
MALTA	✓		
NORWAY	✓		
POLAND	✓		
RUSSIA	✓		
SERBIA	✓		
SLOVENIA	✓		
SWEDEN	✓		
SWITZERLAND	✓		
UK	✓		
UKRAINE	✓		
DENMARK	✓	✓	
ESTONIA	✓	✓	
GREECE	✓	✓	
HUNGARY	✓	✓	
LATVIA	✓	✓	
LITHUANIA	✓	✓	
ROMANIA	✓	✓	
SLOVAKIA	✓	✓	
FRANCE		✓	
PORTUGAL		✓	
TURKEY		✓	
BELGIUM			✓
FINLAND			✓
THE NETHERLANDS			✓

SPAIN			✓
n = 37	n = 30	n = 11	n = 4

Notes

[1] – Excluded countries: Albania, Andorra, Belarus, Lichtenstein, Monaco, Montenegro, San Marino, and Vatican City.

For peer review only

We identified self-regulation in 30 countries in the form of codes issued and overseen by industry trade groups.[19] In 28 of those, the industry codes incorporate the provisions of the EFPIA Code[19 41] as a necessary requirement of trade groups membership in EFPIA. This makes self-regulation the "default approach" to payment disclosure in Europe, with EFPIA holding power to exempt certain countries from following its Code.[42] The first exception is Luxembourg. While the Luxembourgish trade group is not an EFPIA member, it decides voluntarily to implement the regulation of payment disclosure modelled on the EFPIA Code.[43] The second exception is Denmark. Although the Danish trade group is an EFPIA member, EFPIA exempts Denmark from following its Code, given the country's separate public regulation provisions.[42] As the public regulation of payment disclosure in Denmark covers only healthcare professionals,[44] the Danish pharmaceutical trade group developed a code covering only "grants and donations" to hospitals.[45]

We found public regulation in 11 countries. In all cases, it takes the form of government regulation, in which provisions relating to payment disclosure are included either in bespoke new legislation (France, Lithuania, and Romania) or are incorporated into existing pharmaceutical legislation (the remaining countries). In addition, in Greece, the Data Protection Agency made a regulatory intervention by issuing an interpretation of the government regulation.[46]

Only in France, Portugal and Turkey public regulation is the sole regulatory approach, replacing self-regulation entirely. EFPIA excepted France and Portugal from applying the EFPIA Code considering the nature of their public regulation; [42] however, the implementation of the EFPIA Code in Turkey is only suspended while its compatibility with the EFPIA Code is being reviewed.[47]

In the remaining 8 countries with public regulation, there is also parallel self-regulation. In 4 of these (Denmark, Lithuania, Romania, and Slovakia), self- and public regulation cover different donors, payments, or recipients, whereas in the remaining ones (Estonia, Greece, Hungary, and Latvia) donors, recipients and payments disclosed via public and self-regulation may overlap. Consequently, the existence of parallel self-and public regulation in the 8 countries means that self-regulation is used exclusively in 22 of the 30 countries with this approach.

Self- and public regulation are combined as a single approach in four countries. Contrasting with countries with public regulation, here the industry contributes to managing payment disclosure. However, unlike in countries with self-regulation, the industry derives at least some of its regulatory power from public authorities, often sharing it with other stakeholders. In two of the four countries, public regulation takes the form of government regulation (Belgium and Finland) and, and in the two others – regulatory intervention (Spain and the Netherlands).

Belgium regulates payment disclosure via a bespoke "Sunshine Act" but the interpretation of its key provisions is left to betransparent.be, a multi-stakeholder body involving industry and professional organisations,[48 49] which also runs the Transparency Register integrating company disclosures.[50 51] In Finland, new provisions have been introduced into the Medicines Act, stipulating that drug companies "must keep available for public review" a list

For peer review only - http://bmjopen.bmj.com/site/about/guidelines.xhtml

of all payments to “associations in the fields of medicine and health care”[52] but in practice the disclosure takes place following the EFPIA Code.

In Spain, public regulation involves an intervention by the Data Protection Authority[40] confirming that the publication of named payment recipients does not require recipient consent.[27] However, like in Belgium and Finland, disclosure is managed by companies based on the EFPIA Code. In the Netherlands, payments are disclosed using self-regulatory rules developed by the Foundation for the Code for Pharmaceutical Advertising, which are separate from the EFPIA Code. Like in Belgium, the central platform is a multistakeholder body involving the industry and healthcare providers.[53] However, public authorities triggered the policy debate on payment disclosure and, having considered self-regulation preferable to public regulation, they lent it financial support and monitor its performance.[54] [55] Further, consistent with the regulatory intervention in Spain, the Dutch Data Protection Authority confirmed that recipient consent is not required.[55]

3.2. Data disclosed via self-regulation by pharmaceutical companies and trade groups

We were able to collect information on accessibility and quality of payment data in 28 of the 30 countries with self-regulation.

Regarding data accessibility, the EFPIA Code allows companies within each country to disclose payments either on a centralised platform or individual websites.[19] However, only five trade groups have established databases for all companies, including four countries following the EFPIA Code and one using its own Code (Danish Association of the Pharmaceutical Industry, LIF). Of the five industry-run databases none had customisable summary statistics (Table 2). Moreover, only one was fully searchable (i.e. without additional information required for searches) and just two were downloadable. Overall, Disclosure UK had by far the highest data accessibility.

Turning to data quality, only the Czech database used unique donor and recipient identifiers consistently, but, because they were required for searches, they paradoxically decreased data accessibility. The second most frequent problem across the databases was tax reporting. While in the four databases established under the EFPIA Code the rules on tax reporting might be reconstructed using "methodological notes" published separately by each company,[19] the Danish database had no information regarding tax. Taken altogether, Disclosure UK had the highest data quality, although here it was more closely matched by the Czech database.

For peer review only - http://bmjopen.bmj.com/site/about/guidelines.xhtml

Table 2 Accessibility and quality of drug company payment data disclosed via centralised industry databases and eurosfordocs.eu

			Payment data accessibility[1]					Payment data quality[1]			
**Country**	**Name of regulation**	**Overseeing authority and database web link [2, 3, 4]**	**Document format**	**Single data template**	**Database searchable**	**Customisable summary statistics**	**Database downloadable**	**Characteristics included**	**Aggregation of payments**	**Payments with or without taxes**	**Unique identifiers**
**Self-regulation at the European level – minimum requirements**											
EFPIA	EFPIA Code	EFPIA	Not regulated	Yes ("EFPIA disclosure template"), but deviations allowed	Not regulated	Not regulated	Not regulated	Donors; recipients; recipient location; payment categories and amounts; year	Annually per payment type	No single rule, each company sets its own rules for VAT reporting which are published separately from payment disclosures	Optional
**Centralised online industry databases**											
UNITED KINGDOM	ABPI Code of Practice	Association of the British Pharmaceutical Industry	Website, XLS	Yes	Yes	No	Yes	Donors; recipients; recipient categories (healthcare professionals) and location; payment categories and amounts; year; web links with further descriptions for some payments	Annually per payment type for healthcare professionals; payments to healthcare organisations itemised	No single rule, each company sets its own rules for VAT reporting which are published separately from payment disclosures	No
CZECH REPUBLIC	Eticky Kodex AIPF	Asociace inovativního farmaceutického průmyslu	Website	Yes	Yes (but requires donor or recipient identifiers)	No	No	Donors; donor location; recipients; recipient location; payment categories and amounts; year	Annually per payment type	No single rule, each company sets its own rules for VAT reporting which are published separately from payment disclosures	Recipient and donor identifiers
DENMARK	Ethical rules for the pharmaceutical industry's donations and grants	Lægemiddelindustriforeningen	Readable PDFs	Yes	No	No	Yes	Donors; project name; recipients; product name; funded activity; payment goal; timescale of funded activity; payment amount and form (cash or benefit in kind)	No	Unclear	No

For peer review only - http://bmjopen.bmj.com/site/about/guidelines.xhtml

GREECE	SFEE Code of Conduct	Hellenic Association of Pharmaceutical Companies	Website	Yes	No	No	No	Donors; recipients; recipient categories; payment descriptions, categories goals, and amounts; date	No	No single rule, each company sets its own rules for VAT reporting which are published separately from payment disclosures	No
IRELAND	Code of Practice of the Pharmaceutical Industry	Irish Pharmaceutical Healthcare Association	Website	Yes	No	No	No	Donors; recipients; recipient location; payment categories and amounts; year	Annually per payment type	No single rule, each company sets its own rules for VAT reporting which are published separately from payment disclosures	Partial (recipient identifiers used by some companies)
**Industry data integrated within an independent database[2]**											
EUROS FORDOCS. EU	Codes of conduct in countries where data was collected[5]	N/A	Website, XLS	Yes	Yes	Yes	Yes	Donors; recipients; recipient location; payment categories and amounts; year	Annually per payment type for healthcare professionals (all countries); Annually per payment type for healthcare organisations in all countries but the UK, where payments to healthcare organisations are itemised	No single rule, each company sets its own rules for VAT reporting which are published separately from payment disclosures	Spain: recipient identifiers; Other countries: No

Notes

[1] – Lighter colours indicate, respectively, higher, and darker colours – lower, data accessibility and quality. In the upper part of the table, the centralised industry databases are presented in the descending order of their overall data accessibility and quality, that is, the greater overall number of lighter cells a database has the higher its position within the table. Databases with equal numbers of lighter and darker cells are sorted alphabetically.

[2] – The disclosure requirements ordinarily cover both healthcare professionals and organisations. The exceptions are the database run by the Danish pharmaceutical industry trade group (donations to hospitals) and the database run by the Greek pharmaceutical industry trade group (only payments to healthcare organisations).

[3] – Web links are accurate as of May 2021.

[4] – Some pharmaceutical industry trade groups create and delegate some responsibility for the everyday operation of their codes to sub-divisions such as the Ethical Committee for the Pharmaceutical Industry (established by Denmark's LIF) or the Prescription Medicines Code of Practice Authority (established by the UK's ABPI). However, the ultimate responsibility for managing and overseeing the codes is with the trade group.

[5] – Ireland, Italy, Germany, Spain, Sweden, Switzerland, and the UK.

For peer review only

For peer review only - http://bmjopen.bmj.com/site/about/guidelines.xhtml

In 23 of the remaining countries with self-regulation and available data, disclosures were published on individual websites for each company. Of these, in 18 countries, trade groups had the EFPIA-recommended gateways to these websites.[19] Nevertheless, without EFPIA's explicit guidance on the electronic format of disclosure documents disclosures published on company websites in countries with and without gateways were typically PDFs. While some of these documents were "readable", allowing for copying and pasting of information, they offered limited possibilities for efficient searches and integrating data from different companies. Additionally, some companies presented data without strictly following the "EFPIA disclosure template"[19], which further impeded possibilities for cross-company comparisons (Online Supplement 3 has examples of these deviations). Some firms apparently manipulated data presentation using low-resolution, image-based PDFs, which prevented any searches (Online Supplement 4 summarises these techniques).

Given the low accessibility of payment data, analysing its quality was practically impossible in countries without centralised databases. Therefore, we do this using eurosfordocs.eu, a database covering drug company disclosures in countries with self-regulation (Ireland, Italy, Germany, Sweden, Switzerland, and the UK); in this part of the analysis, we also include Spain, a country with a combination of self-and public regulation as it helps illustrate problems characteristic of self-regulation.

3.3. Industry data disclosed via self-regulation and integrated within eurosfordocs.eu

Eurosfordocs.eu had data accessibility superior to all industry-run databases (Table 2). While the Irish and UK databases were also searchable, eurosfordocs.eu offered customisable queries using combinations of donor and recipient names and payment categories. [56] It was the only database offering customisable summary statistics enhancing data exploration. In addition, only eursofordos.eu and Disclosure UK were downloadable for further analysis.

A specific consideration regarding data is estimating how closely eurosfordocs.eu matches the underlying industry disclosures (Table 3). Complete data extraction was only possible in the UK and Ireland, the two countries with centralised trade group databases (Online Supplement 5 summarises the data extraction statistics). Elsewhere data scraping prioritised the 20 largest donors known from the countries with complete data; more data was scraped whenever allowed by formats used by companies.[27] For four of the six countries, the resulting dataset closely or exactly matched the industry's summary country-level data. The two countries with a low match were Germany and Spain, given a high proportion of image-based PDFs hindering data extraction.[27]

For peer review only - http://bmjopen.bmj.com/site/about/guidelines.xhtml

Table 3 Estimation of the comprehensiveness of industry payment data extracted for eurosfordocs.eu (2019)

Country[1]	Total value of payments reported in summary industry data (€m)[2, 3]	Total value of payments extracted to eurosfordocs.eu (€m)[4, 5]	Difference (€m)	Difference as a share of summary industry data (%)[6]	Level of match between summary industry data and eurosfordocs.eu
GERMANY	629	499	130	21%	Low
IRELAND	35	35	0	0%	Exact
SWEDEN	90	82	8	9%	Close
SWITZERLAND	167	155	12	7%	Close
SPAIN	601	337	264	44%	Low
UK	619	611	8	1%[5]	Exact/Close

[1] – Only countries covered by both eurosfordocs.eu and available national-level summary data generated by industry trade groups are included.

[2] – Sources of national-level summary payment data.

- Germany,[57] Spain,[58] Switzerland[59] – publicly available pharmaceutical industry summary data published by the pharmaceutical industry trade groups.
- Ireland – a combination of an Europe-wide report published by EFPIA[60] and email communication with the Irish pharmaceutical industry trade group.[61]
- Sweden – email communication with the pharmaceutical industry trade group.
- The UK – calculations based on data obtained from Disclosure UK, the centralised database of industry payments run by the Association of the British Pharmaceutical Industry.[62]

[3] – All payment values in non-euro currencies were converted to euros based on the average yearly exchanged rates published by the European Central Bank.

[4] – The source of payment values reported in this column are centralised pharmaceutical industry payment databases (Ireland and the UK) and payment reports covering payments made by individual companies (Germany, Spain, Sweden, and Switzerland).

[5] – All payment values in non-euro currencies were converted to euros based the exchange rate obtained from the CurrencyConverter,[63] a Python library for exchange rates.

[6] – Some of the difference between the value of payments based on summary industry data and extracted to eurosfordocs.eu results from the differences in the exchange rates. This is exemplified by the examples of Ireland (both values in euro, no difference) and the UK (original values in the sterling, the difference is caused by different exchange rates used to convert the sterling to euro). By contrast, the 1% difference between eurosfordoscs.eu and Disclosure UK results from two marginally different exchange rates used to convert the sterling to euros

For peer review only

Nevertheless, other aspects of the data quality in eurosfordocs.eu share key limitations with the underlying company disclosures.

First is a narrow spectrum of reported recipient, donor, and payment characteristics. Eurosfordocs.eu does not present payment distribution within the healthcare system due to the incoherent use or omission of recipient categories by drug companies. Of all countries covered by eurosfordocs.eu the UK is the only one where the industry trade group categorised healthcare professionals receiving payments,[64] albeit incoherently;[21] healthcare organisations were nowhere categorised.

Second, consistent with the EFPIA Code[19] payments to healthcare professionals are not itemised but aggregated annually per recipient within each payment category. The same applies to payments to healthcare organisations, except for the UK, where the ABPI mandates that payments to healthcare organisations be itemised.[64] This UK-specific rule might explain the large difference in the number of payments reported with Germany, a country with a similar overall value of payments (Online Supplement 5). However, it is equally possible that not all companies in the remaining six countries covered by the database aggregate payments consistently as some list more than one payment per recipient, which might also indicate that although these recipients have the same names, they are different entities.

Third, the reported payment values must be interpreted cautiously as it is unclear whether they include taxes without consulting the separately published "methodological notes".[19] Some companies have different approaches to tax reporting depending on payment or recipient categories. Consequently, establishing the value of payments made by each company requires additional forensic work.[23]

Finally, while EFPIA introduces the option of unique recipient identifiers in disclosed payment data,[19] of the seven countries covered by eurosfordocs.eu only the Spanish trade group followed this recommendation. Elsewhere the number of recipients per company and, consequently, the value of payments per recipient remains unknown. Given inconsistent naming approaches in disclosures made by the same or different companies, the same recipient can have different names, and, conversely, different recipients may have the same name.[23] Further, the same recipient can be identified at different levels of aggregation (e.g. hospital wards, departments or hospitals), with self-regulation at least in some countries placing the onus of identifying possible multiple records on payment recipients and not companies.[23 65] Lastly, without identifiers payment data cannot be connected to other databases.

3.4. Data disclosed via public regulation or a combination of public and self-regulation

Having examined countries with self-regulation, we proceed to those with public regulation or a combination of public and self-regulation.

For peer review only

For peer review only - http://bmjopen.bmj.com/site/about/guidelines.xhtml

Of the 15 countries with public regulation or a combination of self-and public regulation, all but two had centralised databases. The exceptions were Finland and Spain, where disclosures were made on individual drug company websites, consistent with the EFPIA Code. Of the thirteen countries with centralised databases, one had a database which was not publicly available (Turkey) and two others had separate databases for different payment categories (Denmark) and healthcare professionals and organisations (Greece). As the information included in the separate Danish and Greek databases did not differ according to our outcome measures, we consider them jointly (Table 4).

For peer review only

Table 4 Accessibility and quality of drug company payment data disclosed via public regulation or a combination of self-regulation and public regulation

			Payment data accessibility[1]					Payment data quality[1]			
Country	Name of regulation	Overseeing authority and database web link [2, 3, 4]	Document format	Single data template	Database searchable	Customisable summary statistics	Database downloadable	Characteristics included	Aggregation of payments	Payments with or without taxes	Unique identifiers
FRANCE	Law No. 2011-2012 (Law Bertrand)	Ministry of Social Affairs and Health	Webpage	Yes	Yes	No	Yes	Donors; donor categories; recipients; recipient categories; payment categories and amounts; date; recipient address	No	Inclusive of VAT	Donors (multiple entries for subsidiaries), recipients (partial)
LATVIA	Regulation No. 378 (2014)	Health Inspectorate	XLS	Yes	No	No	Yes	Donors; recipients; recipient categories; payment name, description, category and amount; date; recipient address	No	Unclear	Donors, recipients
BELGIUM	Sunshine Act of 2016	Federal Agency for Medicines and Health Products	Webpage	Yes	Yes	No	No	Donors; recipients; recipient categories; payment categories and amounts; recipient address; years	Annually per payment type	Unclear	Donors, recipients
LITHUANIA	Law on Pharmacy (provisions from 2019), Ministerial Order No. V-1537 (2020)	State Medicines Control Agency	XLS	Yes	No	No	Yes	Donors; recipients; recipient categories; payment name; date; recipient address	No	Unclear	Donors (not publicly available), recipients (publicly available)
PORTUGAL	Decree Law 20/2013 and 128/2013	National Authority of Medicines and Health Products	Webpage	Yes	Yes	No	No	Donors; donor categories; recipients; payment descriptions and amounts; years	No	Inclusive of VAT	No
ROMANIA	Orders of the Minister of Health 194/2015 and 874/2015	National Agency for Medicines and Medical Devices	Webpage	Yes	Yes	No	No	Donors; recipients; recipient categories; payment descriptions, categories, and amounts; recipient address; date	No	Unclear	No
SLOVAKIA	Act No. 362/2011 on Medicines and Medical Devices	National Health Information Center	XLS	Yes	No	No	Yes	Donors; recipients; recipient categories (only healthcare professionals); payment descriptions, categories and amounts; clinical trial numbers; product names; recipient address; date	No	Unclear	No
DENMARK	Health Act of 2014, Executive Order No. 1153	Danish Medicines Agency 1) conferences abroad ; 2) professional affiliations	Webpage	Yes	Yes	No	No	Conferences abroad – donors; recipients; recipient categories; recipient address; Professional affiliations – donors; recipients; recipient categories; recipient address; payment amounts	Annually per payment type	Unclear	Recipients

HUNGARY	Act XCVIII of 2006 (provisions introduced in 2011)	National Institute of Pharmacy and Nutrition	Webpage	Yes	Yes	No	No	Donors; payment names, descriptions and amounts; date; recipient address	No	Unclear	No
THE NETHER-LANDS	Code of Conduct for Pharmaceutical Advertising (2012)	Foundation for the Code for Pharmaceutical Advertising	Webpage	Yes	Yes (recipient identifiers needed)	No	No	Donors; recipients; recipient categories; payment categories and amounts; year	Annually per payment type	Unclear	Recipients
GREECE	Law 4316/2014; Opinion No. 5/2016 and 2/2017 of the Data Protection Authority; circular No. 17770/2016 of the National Authority for Medicines	National Organisation for Medicines 1) payments to conference participants; 2) payments to conference organisers; drug company websites	PDFs – image-based	Yes	No	No	Yes	Payments to conference participants – donors; recipients; payment categories (types of conference expenditure) and amounts; year; Payments to conference organisers – donors; payment amounts; year	Payments to conference participants – annually per recipient; Payments to conference organisers – donors	Unclear	Donors
ESTONIA	Medicinal Products Act of 2005 (provisions introduced in 2013)	State Agency of Medicines	XLS	Yes	No	No	Yes	Donors; payment categories and amounts; payment location (country); year	Annually per donor	Unclear	No
TURKEY	Regulation on Promotional Activities of Medicinal Products for Human Use 2015	Ministry of Health (database not publicly available)	Unclear	Unclear	Unclear	Unclear	Uncle ar	Unclear	Unclear	Unclear	Unclear

Notes.
[1] – Lighter colours indicate, respectively, higher, and darker colours – lower, data accessibility and quality. The databases are presented in the descending order of their overall data accessibility and quality, that is, the greater overall number of lighter cells a database has the higher its position within the table. Databases with equal numbers of lighter and darker cells are sorted alphabetically.
[2] – This column provides the dates when public regulation of payment disclosure was first introduced. If public regulation of payment disclosure forms part of a larger piece of government regulation, it is specified – where appropriate – whether the regulation of payment disclosure was introduced as a change already existing government regulation. The dates reported here do not cover changes to or refinements of provisions focusing on payment disclosure.
[3] – The disclosure requirements ordinarily cover both healthcare professionals and organisations. The exceptions are the Danish databases (only healthcare professionals) and the Turkish database (it is unclear whether disclosure requirements also cover healthcare organisations).
[4] – Web links are accurate as of May 2021.
[5] – The recipient addresses ordinarily refer to the location of the payment recipient. In the case of Hungarian, Latvian and Lithuanian databases we considered that the event addresses were equivalent to recipient addresses.

The databases established via public regulation or a combination of public and self-regulation had the pattern of accessibility similar to the industry-run databases. Of the thirteen databases none had customisable summary statistics, and only six were downloadable and fully searchable. Overall, Transparence Santé was the frontrunner.

The most frequent data quality shortcoming was unclear tax reporting, with only two databases providing relevant rules. However, over half of the databases had at least partial donor or recipient identifiers, which was the most frequent problem in the industry-run databases. Further, just five databases covered a spectrum of donor or recipient characteristics exceeding the minimum recommendations from the EFPIA Code . Transparence Santé again had the highest overall data quality.

In sum, Transparence Santé had combined data accessibility and quality exceeding that of Disclosure UK, the frontrunner industry database.

4. Discussion

Our policy review suggests that payment data disclosure does not automatically increase transparency of financial relationships between drug companies and the healthcare sector.[4 5] Consistently with research on disclosure of aspects of health policymaking by both public and private-sector actors, we find that achieving "practical" or "actionable" transparency is no less important than introducing transparency rules themselves.[66-68]

Although EFPIA calls payment data generated via self-regulation "open to public scrutiny",[69] establishing the entanglement of any recipient, let alone a system-level picture, is impossible given the dispersal of disclosures on company websites in most European countries. Additionally, documents published as PDFs, sometimes in ways suggesting deliberate attempts to impede user engagement, fall below the Australian industry-endorsed regulations requiring firms to use an analysable format.[5] Therefore, self-regulation cannot address "the issues of perceived conflict of interest",[70] as promised by EFPIA. More broadly, the evidence of some companies and trade groups meeting only the minimum requirements from the EFPIA Code, or fulfilling them in ways inconsistent with the Code's spirit, reflects the limited success of self-regulation in modifying corporate behaviour in areas of public health policy such as reduction of sugar content in food[71] or managing viewers' exposure to alcohol advertising.[72]

Eurosfordocs.eu radically enhances data accessibility in countries without centralised industry databases, also enabling comparative investigations of payment patterns,[27] which is important given the accelerating EU-wide health initiatives.[73] Although the customisable opportunities for data exploration are new to the public, data analytics firms have offered them as a consultancy service to drug companies.[74 39] Consequently, eurosfordocs.eu may contribute to changing what may be the de-facto status of payment data as a commodity used to monitor internal compliance with disclosure requirements and potentially inform marketing strategies targeting healthcare professionals.[75]

For peer review only - http://bmjopen.bmj.com/site/about/guidelines.xhtml

In countries with self-regulation, the challenges in data accessibility and quality are exacerbated by non-disclosed payments. Some companies may not disclose all their payments, as suggested by instances of underreporting of payments to patient organisations, with their disclosure also regulated by the EFPIA Code but with distinct policies.[76 77] Further, self-regulation only covers companies and trade groups that have ratified the EFPIA Code or its transposition into country-level codes. Therefore, disclosure requirement may not extend to companies focusing on generic or over-the-counter medicines and even major manufacturers of branded prescription medicines (e.g. Vertex does not follow the ABPI Code). However, some non-member companies may choose to follow the trade group codes. For example, the list of Disclosure UK participants exceeds ABPI membership.[77] Further, some companies may belong to other trade groups (e.g. generic or small biotech trade groups), which, in some countries, require their members to abide by the national Codes (e.g. Sweden, Denmark).

Although data reported in Open Payments has attracted some criticism,[24 78] its accessibility and quality are vastly superior to European data disclosed using self-regulation but also public regulation. Although Transparence Santé indicates that public regulation can generate payment data outpacing industry-run databases, it often shares major shortcomings with self-regulation, including the lack of recipient identifiers or payment itemisation.[23 25] Moreover, in some databases the spectrum of disclosed characteristics is even narrower than the minimum which EFPIA recommends for the industry. Nevertheless, public regulation eliminates optionality characterising the EFPIA Code, regarding, for example, centralised databases. The legally binding nature of public regulation should also involve high levels of compliance. However, instances of inaccurate or incomplete reporting by some companies are possible.[24]

4.1. Limitations

This study has several limitations. Our measures of data accessibility could be expanded. For example, some databases are difficult to find, including web links to the Greek and Latvian databases published within news releases, without permanent online location. Similarly, although Transparence Santé can be downloaded, the size of the dataset prevents it from being opened using the standard Excel package. Data quality could be scrutinised further by considering the types of disclosed donors, payments and recipients.[4] Further, qualitative insights from data users would be essential for ranking the outcome measures and attributing weights to their values, such as degrees of user-friendliness.

Our focus on the database level might obscure cross-company differences. For example, the widely ranging consent rates achieved by companies from healthcare professionals suggests that similar differences can occur in data quality and accessibility.[21 27] Further, we did not calculate company-level aspects of data accessibility (e.g. the share of image-based PDFs) and quality (e.g. the share of duplicate entries, consistency in using donor or recipient categories and identifiers, missing data, and mistakes, such as negative values). Undertaking these calculations would have necessitated extensive forensic work.[23] However, these problems are likely to be widespread and serious, affecting even Transparence Santé, the database we ranked the highest based on its quality .[21 23 25]

For peer review only - http://bmjopen.bmj.com/site/about/guidelines.xhtml

4.2. Conclusions and policy recommendations

We formulate suggestions for enhancing public engagement with disclosed payment data. (Table 5), which are also relevant for non-European countries, such as Japan, experiencing problems similar to those identified in this study.[79]

Table 5 How can public authorities and the pharmaceutical industry improve the transparency of payment data?

	**Recommendations for improving accessibility of payment data**
1	Create national-level databases searchable for companies, recipients, and payment categories.
2	Make the databases in the CSV or XLS format for further analysis, while ensuring that the released data can be split using different variables, for example, by year or recipient type to make it manageable for users.
3	Enable users to explore the data by allowing them to generate data summaries placing payments made or received in a broader context (e.g. payments made by other companies or received by the same or other recipient categories, such as medical specialty).
	**Recommendations for improving quality of payment data**
4	Publish unique identifiers for payment recipients shared by all companies and used consistently over time.
5	Introduce clear rules on the levels of aggregation for identifying recipients (e.g. clinic, ward, or hospital) to enhance the consistency of reporting.
6	Introduce categories of recipients to enable mapping the distribution of payments in the healthcare system. The categories relating to healthcare professionals could include a standardised list of medical specialties. The categories covering healthcare organisations could reflect their functions in the healthcare system as providers, commissioners, or professional organisations.
7	State clearly whether reported payments should include VAT or other taxes so that payment values from different companies can be compared reliably.
8	Publish each payment individually instead of aggregating them annually per recipient.
9	Publish payment descriptions so that the public can understand the activities they fund as well as their context. This requirement would follow the self-regulatory rules existing in relation to the disclosure of payments to patient organisations.
10	Enforce and publish detail of data quality checks: eliminate missing values, payments with the value of zero, and ensure that each recipient has a unique name and is reported at the same level of aggregation by all companies. Other data quality checks should involve cross-checking recipient name and address information to ensure consistency and avoid duplicate reporting.

For peer review only

Payment data accessibility can be easily enhanced with only minor revisions of the existing regulatory approaches, with the top priority being centralised databases offering possibilities for payment exploration and contextualisation.

Improving payment data quality would require new comprehensive public regulation, preferably at the European level.[4,27] Following Open Payments, payments should be reported together with information on related products to allow generating insights into company marketing strategies.[23,80] Another vital piece of information might be the numbers of clinical trials associated with payments, which are listed in the database run by the Slovak National Health Information Center. No less important is granular disclosure allowing for capturing payments of different sizes, with some US studies suggesting that even small payments impact prescribing behaviour,[81,82] while others indicating a more complex dose-effect relationship.[11,28,83,84] Interpretation can be enhanced by descriptions of funded activities (e.g. specific conferences or projects), consistent with the EFPIA Code's requirements regarding payments to patient organisations.[77,85] Recipient characteristics should be also expanded, reflecting how the public engages with the healthcare system.[23] Lastly, the example of Open Payments highlights that recipient identifiers are necessary for reliable analysis and connecting payment data to datasets with details of prescription and procurement.[11,28,81,82,84] Data integration and management should be supported by strong compliance mechanisms, including penalties for providing data of inadequate quality.[24]

Additionally, in European countries with self-regulation, eliminating possibilities for refusing disclosure by recipients is necessary to reduce high levels of missing data.[27] The decision by the Spanish Data Protection Authority is illustrative here, exempting payment data from the provisions of the European data protection legislation (GDPR).[27]

Finally, transparency alone cannot address conflicts of interest. Even in the US, the increased of transparency brought in by Open Payments does not seem have decreased physicians' acceptance of FCOIs or increased patient concerns about their possible effects on the care they receive.[86] Paradoxically, transparency may normalise FCOIs or increase their impact via moral licensing.[86] Therefore, transparency should be accompanied by policy measures seeking to reduce or eliminate certain FCOIs. Key European examples include banning some financial relationships,[87] including payments to healthcare professionals for conference participation in Sweden[27] or prohibiting sponsored meals over €60 in France.[88]

For peer review only - http://bmjopen.bmj.com/site/about/guidelines.xhtml

Contributors

PO is Senior Lecturer at the Department of Social and Policy Sciences, University of Bath. PO conceived and wrote the paper, collected and analysed the data. LM is a data scientist and the President of the Euros for Docs Association. LM created the eurosfordocs.eu database, analysed the data and contributed to writing. PAJ formerly presided the Euros for Docs Association. PAJ collaborated with LM on creating eurosfordocs.eu. PAJ conceived the paper and contributed to writing. SM is Associate Professor at the Department of Sociology, Lund University. SM conceptualized the paper and contributed to writing.

Acknowledgements

We would like to express gratitude to all pharmaceutical industry trade groups and public authorities which have responded to our request for information and were willing to explain the nature of local regulatory solutions. We thank, in particular, the ABPI's Disclosure UK Team for their continued interest in our research as well as professionalism and openness in sharing the fine detail of their important work. We extend our thanks to colleagues who took the time to read drafts of this paper or offer linguistic assistance: Marcell Csanadi, Eniko Csanadi, Alice Fabbri, Kevin Jean, Fatma Korkmaz, Olga Loblova, Savvas Morris, Emily Rickard, Eszter Saghy, Christos Vasilakis, and Weronika Ozieranska.

We are also thankful to the BMJ Open Editors and three Reviewers for their helpful and constructive comments.

Funding

This work was supported by The Swedish Research Council (VR), grant number 2020-01822 ("Following the money: cross-national study of pharmaceutical industry payments to medical associations and patient organisations").

Details of the role of study sponsors

The funding body played no part in the design or conduct of this study

Statement of independence of researchers from funders

All researchers working on this study are independent from the funding body.

Competing interests

We have read and understood the BMJ Group policy on declaration of interests and declare the following interests:

For peer review only

PO's PhD student was supported by a grant from Sigma Pharmaceuticals, a UK pharmacy wholesaler and distributor (not a pharmaceutical company). The PhD work funded by Sigma Pharmaceuticals is unrelated to the subject of this paper.
LM and PAJ are members of Euros for Docs, a non-profit organization registered in France that seeks to promote transparency of drug company funding in the healthcare sector by making payment data accessible and complete across Europe.
PAJ is employed by Haute Autorité de Santé, the French independent health technology assessment organisation.
SM's partner is employed by PRA Health Sciences, a global Contract Research Organization whose costumers include many pharmaceutical companies.

Patient consent

None required.

Ethical approval

No ethical approval was needed. The ethical implications of this study article were approved via a peer ethics review process at the Department of Social and Policy Sciences, University of Bath in April 2016. This study did not require a full ethical approval as it relied on publicly available data aggregated at the organisational or country level.

Data sharing

We have included all relevant data as supplementary information forming part of this submission.

Exclusive license

The Corresponding Author has the right to grant on behalf of all authors and does grant on behalf of all authors, a worldwide licence to the Publishers and its licensees in perpetuity, in all forms, formats and media (whether known now or created in the future), to i) publish, reproduce, distribute, display and store the Contribution, ii) translate the Contribution into other languages, create adaptations, reprints, include within collections and create summaries, extracts and/or, abstracts of the Contribution, iii) create any other derivative work(s) based on the Contribution, iv) to exploit all subsidiary rights in the Contribution, v) the inclusion of electronic links from the Contribution to third party material where-ever it may be located; and, vi) licence any third party to do any or all of the above."

Transparency declaration

The guarantor (Piotr Ozieranski) affirms that this manuscript is an honest, accurate, and transparent account of the study being reported; that no important aspects of the study have been omitted; and that any discrepancies from the study as planned (and, if relevant, registered) have been explained.

For peer review only

References

1. Lo B, Field MJ. *Conflict of Interest in Medical Research, Education, and Practice* 2009
2. Lundh A, Lexchin J, Mintzes B, et al. Industry sponsorship and research outcome. *Cochrane Database of Systematic Reviews* 2017(2) doi: 10.1002/14651858.MR000033.pub3
3. Torgerson T, Wayant C, Cosgrove L, et al. Ten years later: a review of the US 2009 institute of medicine report on conflicts of interest and solutions for further reform. *BMJ Evidence-Based Medicine* 2020:bmjebm-2020-111503. doi: 10.1136/bmjebm-2020-111503
4. Fabbri A, Santos Al, Mezinska S, et al. Sunshine Policies and Murky Shadows in Europe: Disclosure of Pharmaceutical Industry Payments to Health Professionals in Nine European Countries. *International Journal of Health Policy and Management* 2018;7(6):504-09. doi: 10.15171/ijhpm.2018.20
5. Grundy Q, Habibi R, Shnier A, et al. Decoding disclosure: Comparing conflict of interest policy among the United States, France, and Australia. *Health Policy* 2018;122(5):509-18. doi: https://doi.org/10.1016/j.healthpol.2018.03.015
6. Mental Health Europe. Sunshine and transparency laws and regulations and codes across Europe 2017 [updated April 2017. Available from: https://mhe-sme.org/wp-content/uploads/2017/09/Mapping-of-Sunshine-Laws-in-Europe.pdf.
7. Santos A. The sun shines on Europe: transparency of financial relationships in the healthcare sector 2017 [10.
8. McDermott Will & Emery. Snapshot of Sunshine rules in EU countries for the pharmaceutical industry 2014 [updated June 2014. Available from: https://mcdermott-will-emery-2793.docs.contently.com/v/special-report-snapshot-of-sunshine-rules-in-eu-countries-for-the-pharmaceutical-industry1.
9. Moynihan R, Albarqouni L, Nangla C, et al. Financial ties between leaders of influential US professional medical associations and industry: Cross sectional study. *The BMJ* 2020;369 doi: 10.1136/bmj.m1505
10. Garstka ME, Monlezun D, DuCoin C, et al. The Sunshine Act and Surgeons: A Nation-Wide Analysis of Industry Payments to Physicians. *Journal of Surgical Research* 2019;233:41-49. doi: https://doi.org/10.1016/j.jss.2018.07.004
11. Yeh JS, Franklin JM, Avorn J, et al. Association of Industry Payments to Physicians With the Prescribing of Brand-name Statins in Massachusetts. *JAMA Internal Medicine* 2016;176(6):763-68. doi: 10.1001/jamainternmed.2016.1709
12. Mejia J, Mejia A, Pestilli F. Open data on industry payments to healthcare providers reveal potential hidden costs to the public. *Nature Communications* 2019;10(1):4314. doi: 10.1038/s41467-019-12317-z
13. Mitchell AP, Trivedi NU, Gennarelli RL, et al. Are financial payments from the pharmaceutical industry associated with physician prescribing? A systematic review. *Annals of Internal Medicine* 2020;Epub ahead of print doi: doi:10.7326/M20-5665 [published Online First: 24 Nov 2020]
14. Wayant C, Walters C, Zaaza Z, et al. Evaluation of Financial Conflicts of Interest Among Physician-Authors of American College of Rheumatology Clinical Practice Guidelines. *Arthritis & rheumatology* 2020;72(9):1427-34. doi: 10.1002/art.41224
15. Journal of Oral and Maxillofacial Surgery. *Journal of Oral and Maxillofacial Surgery*

For peer review only - http://bmjopen.bmj.com/site/about/guidelines.xhtml

16. El-Rayess H, Khamis AM, Haddad S, et al. Assessing concordance of financial conflicts of interest disclosures with payments' databases: a systematic survey of the health literature. *Journal of clinical epidemiology* 2020;127:19-28. doi: 10.1016/j.jclinepi.2020.06.040
17. Ornstein C, Weber T, Jones RG. We Found Over 700 Doctors Who Were Paid More Than a Million Dollars by Drug and Medical Device Companies, 2019.
18. Department of Justice - Office of Public Affairs. Medtronic to Pay Over $9.2 Million To Settle Allegations of Improper Payments to South Dakota Neurosurgeon, 2020.
19. EFPIA. EFPIA Code of practice 2019 [Available from: https://www.efpia.eu/media/554677/efpia_codes_a5_v3-2021_sm.pdf.
20. Mulinari S, Davis C, Ozieranski P. Failure of Responsive Regulation? Pharmaceutical Marketing, Corporate Impression Management and Off-Label Promotion of Enzalutamide in Europe. *Journal of White Collar and Corporate Crime* 2020:2631309X097047. doi: 10.1177/2631309x20970477
21. Mulinari S, Ozieranski P. Disclosure of payments by pharmaceutical companies to healthcare professionals in the UK: analysis of the Association of the British Pharmaceutical Industry's Disclosure UK database, 2015 and 2016 cohorts. *BMJ Open* 2018;8(10):e023094. doi: 10.1136/bmjopen-2018-023094
22. Adlington K, Godlee F. Disclosure UK: transparency should no longer be an optional extra. *BMJ* 2016;354:i3730. doi: 10.1136/bmj.i3730
23. Ozieranski P, Csanadi M, Rickard E, et al. Analysis of Pharmaceutical Industry Payments to UK Health Care Organizations in 2015. *JAMA Network Open* 2019;2(6):e196253-e53. doi: 10.1001/jamanetworkopen.2019.6253
24. Adashi EY, Cohen IG. Enforcement of the Physician Payments Sunshine Act: Trust and Verify. *JAMA* 2021 doi: 10.1001/jama.2021.13156
25. Stoll M, Hubenschmid L, Koch C, et al. Voluntary disclosures of payments from pharmaceutical companies to healthcare professionals in Germany: a descriptive study of disclosures in 2015 and 2016. *BMJ Open* 2020;10(9):e037395. doi: 10.1136/bmjopen-2020-037395
26. Moriarty F, Larkin J, Fahey T. Payments reported by the pharmaceutical industry in Ireland from 2015 to 2019: an observational study. *Health Policy* 2021 doi: https://doi.org/10.1016/j.healthpol.2021.07.016
27. Mulinari S, Martinon L, Jachiet P-A, et al. Pharmaceutical industry self-regulation and non-transparency: country and company level analysis of payments to healthcare professionals in seven European countries. *Health Policy* 2021 doi: https://doi.org/10.1016/j.healthpol.2021.04.015
28. Goupil B, Balusson F, Naudet F, et al. Association between gifts from pharmaceutical companies to French general practitioners and their drug prescribing patterns in 2016: retrospective study using the French Transparency in Healthcare and National Health Data System databases. *BMJ* 2019;367:l6015. doi: 10.1136/bmj.l6015
29. Moberly T. NHS joint working with industry is out of public sight. *BMJ* 2019;364:l1353. doi: 10.1136/bmj.l1353
30. Moberly T. CCGs fail to declare pharma funding. *BMJ* 2018;360:j5911. doi: 10.1136/bmj.j5911
31. U.S. Attorney's Office - District of New Jersey. Novartis AG and Subsidiaries to Pay $345 Million to Resolve Foreign Corrupt Practices Act Cases 2020 [updated 25th June

For peer review only - http://bmjopen.bmj.com/site/about/guidelines.xhtml

2020. Available from: https://www.justice.gov/usao-nj/pr/novartis-ag-and-subsidiaries-pay-345-million-resolve-foreign-corrupt-practices-act-cases.
32. Securities and Exchange Commission. SEC Charges Eli Lilly and Company with FCPA Violations 2012 [updated 20th December 2012. Available from: https://www.sec.gov/news/press-release/2012-2012-273htm.
33. Eurosfordocs.eu. Metabase 2021 [Available from: https://eurosfordocs.eu/metabase/.
34. Anon. Hôpitaux : les CHU ont reçu 170 millions d'euros de laboratoires pharmaceutiques en 2018 2020 [updated 10th January 2020. Available from: https://www.lemonde.fr/sante/article/2020/01/10/hopitaux-les-chu-ont-recu-170-millions-d-euros-de-laboratoires-pharmaceutiques-en-2018_6025479_1651302.html
35. Anon. Procès du Mediator : « Le terreau des conflits d'intérêts à l'hôpital reste fertile » 2020 [updated 4th February 2020. Available from: https://www.lemonde.fr/idees/article/2020/02/04/le-terreau-des-conflits-d-interets-a-l-hopital-reste-fertile_6028302_3232.html.
36. Condomines A. Qu'est-ce que la plateforme EurosForDocs, qui recense les liens d'intérêts avec les industriels pharmaceutiques? 2021 [updated 4th January 2021. Available from: https://www.liberation.fr/checknews/2021/01/04/qu-est-ce-que-la-plateforme-eurosfordocs-qui-recense-les-liens-d-interets-avec-les-industriels-pharm_1809498.
37. Eaton L. Declarations of pharma funding to doctors are hit by covid-19 crisis. *BMJ* 2020;370:m2689. doi: 10.1136/bmj.m2689
38. Zetterqvist AV, Merlo J, Mulinari S. Complaints, Complainants, and Rulings Regarding Drug Promotion in the United Kingdom and Sweden 2004–2012: A Quantitative and Qualitative Study of Pharmaceutical Industry Self-Regulation. *PLOS Medicine* 2015;12(2):e1001785. doi: 10.1371/journal.pmed.1001785
39. MediSpend. Global Compliance Platform 2021 [Available from: https://www.medispend.com/solutions/global-compliance/ accessed 9th January 2021 2021.
40. Agencia Española de Protección de Datos. INFORME 1438818/2016 2016 [
41. EFPIA. EFPIA HCP/HCO DISCLOSURE CODE. EFPIA CODE ON DISCLOSURE OF TRANSFERS OF VALUE FROM PHARMACEUTICAL COMPANIES TO HEALTHCARE PROFESSIONALS AND HEALTHCARE ORGANISATIONS 2014 [Available from: https://www.efpia.eu/media/25837/efpia-disclosure-code.pdf.
42. Shire. EFPIA Disclosure Code 2018 Disclosures Shire Pharmaceuticals 2018 [Available from: https://www.takeda.com/4ab3b3/siteassets/system/who-we-are/values-and-corporate-governance/transparency-disclosures/efpia-disclosure-code-reports/2018/methodology_serbia_english.pdf.
43. APL. Code de déontologie 2020 [Available from: https://www.apl-pharma.lu/docs/33850f/text/2021-code-de-deontologie-apl.pdf.
44. Danish Medicines Agency. Healthcare professionals having a relationship with a company 2020 [updated 23 November 2020. Available from: https://laegemiddelstyrelsen.dk/en/licensing/relationships/ accessed 17th August 2021.
45. Ethical Committe for the Pharmaceutical Industry. Ethical rules for the pharmaceutical industry's donations and grants (Donation Code) N/A [Available from: https://www.enli.dk/media/49893/ethical-rules-on-donations-and-grants-version-20.pdf.

For peer review only - http://bmjopen.bmj.com/site/about/guidelines.xhtml

46. Amgen. Methodological Note. EFPIA & Local Industry Association Disclosure Codes 2020 [Available from: https://search.disclosureuk.org.uk/Public/GetMethodNoteFileForDownload?year=2019&pharmaCompanyId=29 accessed 25th February 2020.
47. GSK. Engaging with healthcare professionals - Turkey 2020 [Available from: https://www.gsk.com/en-gb/responsibility/operating-responsibly/engaging-with-healthcare-professionals/europe/turkey/ accessed 18th January 2021.
48. betransparent.be. About us 2020 [Available from: https://www.betransparent.be/en/about-us/ accessed 18th January 2020.
49. Sunshine act 2016 [Available from: https://www.betransparent.be/wp-content/uploads/2017/01/Moniteur-Belge-2016-12-27-Belgisch-Staatsblad.pdf.
50. betransparent.be. Transparency in the interest of the patient 2020 [Available from: https://www.betransparent.be/en/about-the-transparency-register/ accessed 18th January 2020.
51. GSK. Engaging with healthcare professionals - Belgium 2020 [Available from: https://www.gsk.com/en-gb/responsibility/operating-responsibly/engaging-with-healthcare-professionals/europe/belgium/ accessed 18th January 2021.
52. Medicines Act 395/1987 1987 [Available from: https://www.fimea.fi/documents/160140/765540/18580_Laakelaki_englanniksi_paivitetty_5_2011.pdf.
53. Stichting Transparantieregister Zorg. Over het Transparantieregister 2020 [Available from: https://www.transparantieregister.nl/over-het-transparantieregister accessed 18th January 2020.
54. Stichting Transparantieregister Zorg. Hoe werken we? 2020 [Available from: https://www.transparantieregister.nl/over-het-transparantieregister/hoe-werken-we accessed 28th February 2021.
55. Stichting Transparantieregister Zorg. Veelgestelde vragen 2020 [Available from: https://www.transparantieregister.nl/veelgestelde-vragen.
56. Eurosfordocs.eu. Tech documentation 2021 [Available from: https://eurosfordocs.eu/on-the-tech-side/tech-documentation/ accessed 9th January 2021.
57. FSA. Transparenzkodex 2020 [updated 30th June 2020. Available from: https://www.fsa-pharma.de/de/mitteilungen/presse/archiv/transparenzveroffentlichungen-2019/.
58. FARMAINDUSTRIA. Las compañías farmacéuticas dedicaron el año pasado 483 millones a actividades de investigación y formación científica con organizaciones y profesionales sanitarios 2020 [updated 26th June 2020. Available from: https://www.farmaindustria.es/web/prensa/notas-de-prensa/2020/06/26/las-companias-farmaceuticas-dedicaron-el-ano-pasado-483-millones-a-actividades-de-investigacion-y-formacion-cientifica-con-organizaciones-y-profesionales-sanitarios/.
59. ScienceIndustries. Disclosure obligation since 2016 2020 [updated 22nd September 2020. Available from: https://www.scienceindustries.ch/en/article/13958/disclosure-obligation-since-2016.
60. EFPIA. Report on Ethics & Compliance Activities 2020 [Available from: https://www.efpia.eu/media/554639/efpia-code-report-2019.pdf.
61. IPHA. Email communication, 2020.

For peer review only

62. ABPI. Disclosure UK 2020 [Available from: https://www.abpi.org.uk/our-ethics/disclosure-uk/.
63. CurrencyConverter. CurrencyConverter 0.14.4 2020 [Available from: https://pypi.org/project/CurrencyConverter/ accessed 19th January 2021.
64. ABPI. Code of practice for the pharmaceutical industry 2019 [Available from: https://www.abpi.org.uk/media/6655/abpi-code-of-practice-2019.pdf.
65. ABPI. Email communication, 2021.
66. Ozierański P, Löblová O, Nicholls N, et al. Transparency in practice: Evidence from 'verification analyses' issued by the Polish Agency for Health Technology Assessment in 2012–2015. *Health Economics, Policy and Law* 2019;14(2):182-204. doi: 10.1017/S1744133117000342 [published Online First: 2018/01/08]
67. Bullement A, Taylor M, McMordie ST, et al. NICE, in Confidence: An Assessment of Redaction to Obscure Confidential Information in Single Technology Appraisals by the National Institute for Health and Care Excellence. *PharmacoEconomics* 2019;37(11):1383-90. doi: 10.1007/s40273-019-00818-0
68. Csanádi M, Ozierański P, Löblová O, et al. Shedding light on the HTA consultancy market: Insights from Poland. *Health Policy* 2019;123(12):1237-43. doi: https://doi.org/10.1016/j.healthpol.2019.08.008
69. EFPIA. Annual Review of 2011 and Outlook for 2012 2011 [Available from: https://www.efpia.eu/media/25261/annual-review-of-2011-and-outlook-for-2012.pdf.
70. EFPIA. Annual Review of 2011 and Outlook for 2012. 2011
71. Seferidi P, Millett C, Laverty AA. Industry self-regulation fails to deliver healthier diets, again. *BMJ* 2021;372:m4762. doi: 10.1136/bmj.m4762
72. Noel JK, Babor TF, Robaina K. Industry self-regulation of alcohol marketing: a systematic review of content and exposure research. *Addiction* 2017;112(S1):28-50. doi: https://doi.org/10.1111/add.13410
73. European Commission. COMMUNICATION FROM THE COMMISSION TO THE EUROPEAN PARLIAMENT, THE COUNCIL, THE EUROPEAN ECONOMIC AND SOCIAL COMMITTEE AND THE COMMITTEE OF THE REGIONS Pharmaceutical Strategy for Europe 2020 [Available from: https://eur-lex.europa.eu/legal-content/EN/TXT/?uri=CELEX:52020DC0761.
74. IQVIA. Get to better business processes with embedded compliance 2021 [Available from: https://www.iqvia.com/solutions/integrated-global-compliance/commercial-compliance/ accessed 9th January 2021 2021.
75. Anand R, Collier D, Jiang Y, et al. Leveraging CMS Open Payments Data to Identify Channel Preferences and Gather Competitive Intelligence, Thereby Improving HCP Targeting ND [Available from: https://www.pmsa.org/jpmsa-vol05-article06.
76. Ozieranski P, Csanadi M, Rickard E, et al. Under-reported relationship: a comparative study of pharmaceutical industry and patient organisation payment disclosures in the UK (2012–2016). *BMJ Open* 2020;in press
77. Rickard E, Ozieranski P, Mulinari S. Evaluating the transparency of pharmaceutical company disclosure of payments to patient organisations in the UK. *Health Policy* 2019
78. Department of Health and Human Services - Office of Inspector General. Open Payments Data: Review of Accuracy, Precision, and Consistency in Reporting 2018 [Available from: https://oig.hhs.gov/oei/reports/oei-03-15-00220.pdf.

For peer review only - http://bmjopen.bmj.com/site/about/guidelines.xhtml

79. Ozaki A, Saito H, Senoo Y, et al. Overview and transparency of non-research payments to healthcare organizations and healthcare professionals from pharmaceutical companies in Japan: Analysis of payment data in 2016. *Health Policy* 2020;124(7):727-35. doi: https://doi.org/10.1016/j.healthpol.2020.03.011
80. Greenway T, Ross JS. US drug marketing: how does promotion correspond with health value? *BMJ* 2017;357:j1855. doi: 10.1136/bmj.j1855
81. DeJong C, Aguilar T, Tseng C-W, et al. Pharmaceutical Industry–Sponsored Meals and Physician Prescribing Patterns for Medicare Beneficiaries. *JAMA Internal Medicine* 2016;176(8):1114-22. doi: 10.1001/jamainternmed.2016.2765
82. Fleischman W, Agrawal S, King M, et al. Association between payments from manufacturers of pharmaceuticals to physicians and regional prescribing: cross sectional ecological study. *BMJ* 2016;354:i4189. doi: 10.1136/bmj.i4189
83. Mitchell AP, Trivedi NU, Gennarelli RL, et al. Are Financial Payments From the Pharmaceutical Industry Associated With Physician Prescribing? *Annals of Internal Medicine* 2020;174(3):353-61. doi: 10.7326/M20-5665
84. Nguyen TD, Bradford WD, Simon KI. Pharmaceutical payments to physicians may increase prescribing for opioids. *Addiction* 2019;114(6):1051-59. doi: https://doi.org/10.1111/add.14509
85. Ozieranski P, Rickard E, Mulinari, Shai. Exposing drug industry funding of UK patient organisations. *BMJ* 2019;365:l1806. doi: 10.1136/bmj.l1806
86. Lexchin J, Fugh-Berman A. A Ray of Sunshine: Transparency in Physician-Industry Relationships Is Not Enough. *Journal of General Internal Medicine* 2021 doi: 10.1007/s11606-021-06657-0
87. Parker L, Karanges EA, Bero L. Changes in the type and amount of spending disclosed by Australian pharmaceutical companies: an observational study. *BMJ Open* 2019;9(2):e024928. doi: 10.1136/bmjopen-2018-024928
88. Ganem-Chabenet D. LOI ANTI-CADEAU : LES NOUVEAUTÉS DU DÉCRET DU 15 JUIN 2020 SUR LES AVANTAGES OFFERTS AUX PROFESSIONNELS DE SANTÉ 2020 [Available from: https://emservice.fr/ems/index.php/2020/09/10/breve_dgc_septembre_2020/ accessed 19th March 2021.

For peer review only

Online Supplement 1. Stakeholder survey

Stakeholder survey, Part 1: Questions to national pharmaceutical industry trade groups

Dear Sir or Madam

I would be grateful if you could answer a few questions listed below regarding the way in which your organisation and its Members disclose transfers of value to healthcare professionals and organisations as well as patient organisations. Your answers would be very helpful in informing scholarly publications on which I am currently working that are seeking to present a full and accurate picture of transfers of value in Europe. Please let me know should you have any questions about this research.

[if relevant] In particular, your answers would help me expand on the information your Association provided in a recent EFPIA report and on its webpages, including here. Some of the matters covered in my questions have been touched upon in the sources of data referred to above, although incompletely. I would therefore be grateful if you could answer my questions so that I report correct and accurate information in any research outputs.

Thank you very much in anticipation for your valuable time in answering my questions.

I look forward to hearing from you.

Sincerely yours

Piotr Ozieranski

For peer review only

I would be grateful if you could answer the following questions regarding your Association

1. What is the number of member companies in your Association?
2. How many companies which are not members of your Association follow its Code of Practice?
3. Please could you send me the most recent version of your Association's code of practice (if available, I would appreciate an English version of the document)?

Please could you answer the following questions regarding the platform and mechanisms of disclosure of transfers of value.

4. What is the platform for disclosing transfers of value to healthcare professionals and organisations (e.g. individual pharmaceutical company websites, a single database of transfers of value from all companies)?
5. If transfers of value are disclosed on individual pharmaceutical company websites, does your Association have a website providing links to these websites? If so, please could you provide me with a web link?
6. If transfers of value are disclosed on individual pharmaceutical company websites, has your Association considered creating a single database of transfers of value from all companies?
7. Do healthcare professionals in your country have to consent to the transfers of value being publicly disclosed; or is the disclosure of transfers of value to healthcare professionals mandatory (i.e. healthcare professionals are not asked to consent)? If the disclosure of transfers of value they received is mandatory, please could you state the regulation which makes them mandatory?
8. If healthcare professionals in your country have to consent to the transfers of value being publicly disclosed, does your Association have any expectations regarding the minimum HCP consent rate that should be achieved by companies signing up to its Code of Practice? If so, please could you specify this consent rate? Further, does your Association request companies achieving lower-than-expected healthcare professional consent rates to explain why this might be the case and identify possible ways of improving the consent rates?
9. If healthcare professionals in your country have to consent to the transfers of value being publicly disclosed, has your association considered implementing a simple summary disclosure rate statistic that is easily comparable between companies, such as the percent of the amount of transfers of value disclosed in the aggregate and/or the percent of healthcare professionals disclosed in the aggregate?
10. If healthcare professionals in your country have to consent to the transfers of value being publicly disclosed, does your association have any specific guidelines on how HCP disclosure rates could be improved by its Member Companies? If so, I would be grateful if you could share these guidelines with me?
11. If healthcare organisations have to consent to the transfers of value being publicly disclosed could you provide examples of reasons that would necessitate placing them in the aggregate disclosure?
12. Please could you state the threshold for the acceptable value of meals and drinks that was set by your association?
13. If some transfers of value need to be disclosed in a database run by a public institution, are there any transfers of value (e.g. research and development) that are still disclosed by pharmaceutical companies themselves. If so, I would be grateful if you could specify what these transfers of value are and why they are disclosed by pharmaceutical companies.
14. As far as your Association is aware, do companies disclosing their transfers of value use "unique country identifiers" recommended in the EFPIA Code of Practice, and not only recipient names or locations, to distinguish payment recipients? If so, are these identifiers

For peer review only

shared among different companies so that they can identify the same recipient using the same identifier?

15. Did members of your Association (or other companies following your Association's code of practice) disclose transfers of value to healthcare professionals and organisations in 2020 (i.e. covering payments made in the 2019 calendar year)?

If members of your Association (or other companies following your organisation's code of practice) disclosed transfers of value to healthcare professionals and organisations in 2020 (i.e. covering the 2019 calendar year) please could you answer the following questions.

16. How many pharmaceutical companies in total disclosed transfers of value to healthcare professionals and organisations made in 2019? How many of those companies were members of your Association?
17. Is the number of companies which did not disclose any transfers of value made in 2019 known? If so, please could you state this number? How many of those companies were members of your Association?
18. What was the overall share of healthcare professionals consenting to their transfers of value being disclosed in 2019?
19. What was the overall share of healthcare organisations consenting to their transfers of value being disclosed in 2019?
20. What was the total value of all non-research transfers of value to all healthcare professionals and organisations made in 2019?
21. What was the total value of all non-research transfers of value to all healthcare organisations made in 2019?
22. What was the value of all research-related (i.e. R&D) transfers of value to healthcare professionals or organisations made in 2019?
23. What share of the overall value of transfers of value made to healthcare professionals disclosed at the individual level in your country in 2019?
24. What share of the overall value of transfers of value made to healthcare organisations was disclosed at the individual level in your country in 2019?
25. What was the value of grants and donations made to healthcare organisations in 2019?
26. What was the value of fees for services and consultancy made to healthcare organisations in 2019?
27. What was the value of contributions to costs of events made to healthcare organisations in 2019?
28. What was the value of fees for services and consultancy made to healthcare professionals in 2019?
29. What was the value of contributions to costs of events made to healthcare professionals in 2019?

Finally, I would be grateful if you could answer the following questions regarding the public disclosure of transfers of value to patient organisations.

30. Please could you send me the most recent version of your Association's code of practice as it relates to working with patient organisations (if available, I would appreciate an English version of the document)?
31. Are there any companies that are not members of your Association which disclose transfers of value to patient organisations in line with the EFPIA Code of practice?
32. How many companies disclosed transfers of value to patient organisations made in 2019? How many of those companies were members of your Association?
33. What was the total value of all transfers of value to patient organisations made in 2019?

For peer review only

Stakeholder survey, Part 2. Questions to public or multistakeholder bodies overseeing pharmaceutical industry payment disclosure

Dear Sir or Madam

I would be grateful if you could answer a few questions listed below regarding the way in which your institution discloses payments made by drug companies to individuals and organisations within the healthcare system in your country. Your answers would be very helpful in informing scholarly publications on which I am currently working that are seeking to present a full and accurate picture of payments made by the pharmaceutical industry in Europe. Please let me know should you have any questions about this research.

Some of the matters covered in my questions have been touched upon on your website. However, I would be extremely grateful for your answers to avoid any misunderstandings which may be caused by language issues, particularly as they relate to complex regulatory matters.

Thank you very much in anticipation for your valuable time in answering my questions.

I look forward to hearing from you.

Sincerely yours

Piotr Ozieranski

For peer review only

I would be grateful if you could answer the following questions regarding the nature of payments disclosed by [insert name of institution]

1. What industries are covered by the disclosure requirements (e.g. pharmaceutical, medical device, veterinary)?
2. Please could you briefly describe what is meant by each type of the industry covered by the disclosure?
3. What types of payment recipients are covered by the disclosure requirements (e.g. healthcare professionals, healthcare organisations, patient organisations)? Are these definitions consistent with the ones used in the EFPIA Code of Practice (https://www.efpia.eu/media/554677/efpia-code-2020.pdf) ?
4. Please could you briefly describe what is meant by each type of recipient (e.g. what is meant by the healthcare processional or healthcare organisation)? Are these definitions consistent with the ones used in the EFPIA Code of Practice?
5. What are the categories of payments covered by the disclosure managed by your institution (e.g. sponsorship of conference attendance, consultancy fees, research and development)? Are they the same one as those used in the EFPIA Code of Practice?
6. Please could you briefly describe what is meant by each type of payment (e.g. what is meant sponsorship of by conference attendance)? Are these definitions consistent with the ones used in the EFPIA Code of Practice?
7. If the definitions of payments or recipients used by your institution are not consistent with the ones used in the EFPIA Code of Practice, please could you explain why this is the case? In other words, why alternative definitions have been created for the reporting purposes of your institution as opposed to using the ones introduced by the EFPIA Code of Practice?
8. Are all payments to all payment recipients publicly disclosed by your organisations or are any payments or recipient types exempted from public disclosure?
9. Are payments reported by your institution reported on an individual basis (i.e. each payment has a separate database entry) or are they aggregated on a yearly basis (i.e. all payments of a certain type from a certain company are reported jointly)?
10. What are the responsibilities of companies making payments in relation to their disclosure?
11. What are the responsibilities of payment recipients in relation to their disclosure (e.g. do they need to disclose payments themselves or just verify disclosures made by companies making payments)?
12. Are payment disclosures managed by your institution reported based on calendar years or financial years? If payments are made based on financial years, please could you specify what are the start and end dates of a financial year in your country?
13. Are payment values made publicly available by your institution the same as those received by payment recipients (e.g. do they include the value of any taxes, such as VAT, paid by companies making the payments)?
14. If payments reported in by your institution are not reported consistently with or without relevant taxes (e.g. VAT), where might database users find information on approaches to tax reporting taken by each company making payments.
15. Do all industries covered by the payment disclosures managed by your institution report payments in exactly the same way (e.g. using the same definitions of payments, payment categories and recipients)?
16. Is the database of payments managed by your institution downloadable or not? Please could you explain why the decision has been made to make it downloadable (or not)?
17. Please could you send me the most recent copy of the regulation and/or policy which governs the disclosure of payments made your institution (if available, I would appreciate an English version of the document)?

For peer review only

Please could you answer the following questions regarding the disclosure requirements covered by your institution and the self-regulatory disclosure system overseen by the European Federation of Pharmaceutical Industries (EFPIA), as described in its Code of Practice?

18. Do pharmaceutical companies in your country have to disclose payments with your institution as well as in line with the disclosure system managed by EFPIA (i.e. do companies need to disclose payments to healthcare professionals and organisations with your institution as well as using disclosure reports comprising payments to healthcare professionals and organisations published on their individual websites)?
19. The EFPIA code of practice covers the category of payments related to research and development. Are these payments disclosed by your institution on a named basis or on drug company websites, in line with the EFPIA code of practice?

If payments made in 2019 calendar were reported year please could you answer the following questions regarding payments from <u>all industries</u> whose payments are managed by your institution?

20. How many companies reported payments in 2019?
21. What was the overall number of healthcare professionals receiving payments in 2019?
22. (If relevant) What was the overall number of healthcare organisations receiving payments in 2019?
23. (If relevant) What was the overall number of patient organisations receiving payments in 2019?
24. What was the overall value of payments made to healthcare professionals in 2019?
25. (If relevant) What was the overall value of payments made to healthcare organisations in 2019?
26. (If relevant) What was the overall value of payments made to patient organisations in 2019?

Please could you answer the following questions regarding only payments made by <u>pharmaceutical companies only</u> in the 2019 calendar year?

27. How many pharmaceutical companies reported payments in 2019?
28. Is the number of companies which did not disclose any payments made in 2019 known? If so, please could you state this number?
29. What was the overall number of healthcare professionals receiving payments from pharmaceutical companies in 2019?
30. (If relevant) What was the overall number of healthcare organisations receiving payments receiving payments from pharmaceutical companies in 2019?
31. (If relevant) What was the overall number of patient organisations receiving payments receiving payments from pharmaceutical companies in 2019?
32. What was the overall share of healthcare professionals consenting to their payments being disclosed in 2019?
33. (If relevant) What was the overall share of healthcare organisations consenting to their payments being disclosed in 2019?
34. (If relevant) What was the overall share of patient organisations consenting to their payments being disclosed in 2019?
35. What was the overall value of all payments made to healthcare professionals by pharmaceutical companies in 2019?
36. (If relevant) What was the overall value of payments made to healthcare organisations by pharmaceutical companies in 2019?

For peer review only

37. (If relevant) What was the overall value of payments made to patient organisations by pharmaceutical companies in 2019?
38. Please could you provide the value of each category of payments received by healthcare professionals from pharmaceutical companies in 2019?
39. (If relevant) Please could you provide overall the value of each category of payments received by healthcare organisations from pharmaceutical companies in 2019?
40. (If relevant) Please could you provide overall the value of each category of payments received by patient organisations from pharmaceutical companies in 2019?

Finally, I would be grateful if you could answer a few general questions regarding the direction nature of payments disclosed.

41. The obligatory disclosure of payments by a public body, such as your institution, seem be at odds the approach taken by many other European countries, which support a self-regulatory system managed by the pharmaceutical industry and allowing payment recipients not to have their payments disclosed based on the General Data Protection Regulation (GDPR). How does the system for payment disclosure managed by your institution addresses potential concerns regarding data privacy of payment recipients?
42. What are the advantages of the mandatory disclosure of payments, overseen by your institution, over a self-regulatory payment system based on the EFPIA Code of practice, existing in many other European countries?
43. What is the source of funding of the disclosure system managed by your institution (e.g. general taxation, health insurance, company fees)?
44. What is the yearly cost of maintaining the disclosure system managed by your institution?
45. How there been any examples of healthcare professionals not complying with the requirements of the disclosure system managed by your institution? If so, how were they addressed?
46. (if relevant) How there been any examples of healthcare organisations not complying with the requirements of the disclosure system managed by your institution? If so, how were they addressed?
47. (if relevant) How there been any examples of patient organisations not complying with the requirements of the disclosure system managed by your institution? If so, how were they addressed?
48. How there been any examples of pharmaceutical companies not complying with the requirements of the disclosure system managed by your institution? If so, how were they addressed?
49. Does your institution monitor who uses the disclosed payment data? If so, would you be able to say who the key types of users are?
50. What does your institution do to encourage the use of disclosed data (e.g. public campaigns)?

For peer review only

Online Supplement 2. Responses to stakeholder survey

Part 1. Pharmaceutical industry trade groups

Country	Name of pharmaceutical industry trade group	Reply received	Nature of reply
AUSTRIA	Fachverband der Chemischen Industrie Österreichs	Yes	Answered all questions
BOSNIA AND HERZEGOVINA	Association of Research-based Medicine Producers in Bosnia & Herzegovina	No	N/A
BULGARIA	Association of the Research-based Pharmaceutical Manufacturers in Bulgaria	No	N/A
CROATIA	iF! – Inovativna farmaceutska inicijativa	No	N/A
CYPRUS	The Cyprus Association of Research and Development Pharmaceutical Companies	No	N/A
CZECH REPUBLIC	Asociace inovativního farmaceutického průmyslu	Yes	Answered some questions
DENMARK	LægemiddelindustriforeningenLersø	Yes	Answered some questions
ESTONIA	The Association of Pharmaceutical Manufacturers in Estonia	No	N/A
FINLAND	Lääketeollisuus	Yes	Answered all questions
GERMANY	Freiwillige Selbstkontrolle für die Arzneimittelindustrie	No	N/A
GREECE	Hellenic Association of Pharmaceutical Companies	No	N/A
HUNGARY	Association of Innovative Pharmaceutical Manufacturers	No	N/A
ICELAND	Icelandic Association of the Pharmaceutical Industry	Yes	Holding message
IRELAND	Irish Pharmaceutical Healthcare Association	Yes	Answered all questions
ITALY	Associazione delle imprese del farmaco	No	N/A
LATVIA	Association of International Innovative Pharmaceuticals Producers	Yes	Answered some questions
LITHUANIA	Innovative Pharmaceutical Industry Association	Yes	Answered all questions
LUXEMBOURG	Association pharmaceutique luxembourgeoise	Yes	Answered all questions
NORTH MACEDONIA	Association of Foreign Innovative Manufacturers in Macedonia	Yes	Answered all questions
NORWAY	Legemiddelindustrien	Yes	Answered all questions
POLAND	Związek Pracodawców Innowacyjnych Firm Farmaceutycznych	Yes	Holding message

For peer review only

PORTUGAL	Associação Portuguesa da Indústria Farmacêutica	No	N/A
ROMANIA	Association of International Medicines Manufacturers	No	N/A
RUSSIA	Association of International Pharmaceutical Manufacturers	No	N/A
SERBIA	Innovative Drug Manufacturers' Association	No	N/A
SLOVAKIA	Association of the Innovative Pharmaceutical Industry	No	N/A
SLOVENIA	Forum of International Research and Development Pharmaceutical Companies, EIG	No	N/A
SPAIN	Asociación Nacional Empresarial de la Industria Farmacéutica	Yes	Answered some questions
SWEDEN	Läkemedelsindustriföreningen	Yes	Answered all questions
SWITZERLAND	Science Industries Switzerland	Yes	Holding message
TURKEY	Araştırmacı İlaç Firmaları Derneği	Yes	Answered all questions
UK	Association of the British Pharmaceutical Industry	Yes	Answered all questions
UKRAINE	Association of Pharmaceutical Research and Development	No	N/A
EUROPE	EFPIA	No	N/A

For peer review only

Part 2. Public and multistakeholder bodies overseeing payment disclosure

Country	Name of public or multistakeholder body overseeing payment disclosure	Nature of authority overseeing payment disclosure	Reply received	Nature of reply
BELGIUM	betransparent.be	Multistakeholder body	No	N/A
DENMARK	Danish Medicines Agency	Public body	Yes	Answered some questions
ESTONIA	State Agency of Medicines	Public body	No	N/A
FRANCE	Ministry of Health	Public body	Yes	Holding message
GREECE	National Organisation for Medicines	Public body	No	N/A
HUNGARY	National Institute of Pharmacy and Nutrition	Public body	Yes	Answered all questions
LATVIA	Latvian Health Inspectorate	Public body	Yes	Answered all questions
LITHUANIA	Lithuanian State Medicines Control Agency	Public body	Yes	Answered all questions
THE NETHERLANDS	Vereniging Innovatieve Geneesmiddelen	Multistakeholder body	Yes	Inquiry redirected
PORTUGAL	INFARMED - National Authority of Medicines and Health Products	Public body	Yes	Answered some questions
ROMANIA	National Agency for Medicines and Medical Devices in Romania	Public body	Yes	Holding message
SLOVAKIA	National Health Information Center	Public body	Yes	Answered some questions
TURKEY	Turkish Medicines and Medical Devices Agency	Public body	Yes	Holding message

For peer review only

Online Supplement 3. Examples of deviations of data reporting formats from the EFPIA standard "disclosure template"

Part 1: EFPIA "disclosure template"

ANNEX A (binding)
Standardised disclosure template

ANNEX A - STANDARDISED DISCLOSURE TEMPLATE

Date of publication: ....

	Full Name	**HCPs:** City of Principal Practice HCO**s**: city where registered	Country of Principal Practice	Principal Practice Address	Unique country identifier *OPTIONAL*	Donations and Grants to HCOs *(Art. 3.01.1.a)*	Contribution to costs of Events *(Art. 3.01.1.b & 3.01.2.a)*			Fee for service and consultancy *(Art. 3.01.1.c & 3.01.2.c)*			**TOTAL** *OPTIONAL*
	(Art. 1.01)	*(Art. 3)*	*(Schedule 1)*	*(Art. 3)*	*(Art. 3)*		Sponsorship agreements with HCOs / third parties appointed by HCOs to manage an Event	Registration Fees	Travel & Accommodation	Fees	Related expenses agreed in the fee for service or consultancy contract, including travel & accommodation relevant to the contract		
**HCPs**	***INDIVIDUAL NAMED DISCLOSURE - one line per HCP*** *(i.e. all transfers of value during a year for an individual HCP will be summed up: itemization should be available for the individual Recipient or public authorities' consultation only, as appropriate)*												
	Dr A					N/A	N/A	*Yearly amount*	*Yearly amount*	*Yearly amount*	*Yearly amount*		
	Dr B					N/A	N/A	*Yearly amount*	*Yearly amount*	*Yearly amount*	*Yearly amount*		
	etc.					N/A	N/A	*Yearly amount*	*Yearly amount*	*Yearly amount*	*Yearly amount*		
	OTHER, NOT INCLUDED ABOVE - where information cannot be disclosed on an individual basis for legal reasons												
	**Aggregate amount attributable to transfers of value to such Recipients** - *Art. 3.02*					N/A	N/A	*Aggregate HCPs*	*Aggregate HCPs*	*Aggregate HCPs*	*Aggregate HCPs*		*Optional*
	**Number of Recipients in aggregate disclosure** - *Art. 3.02*					N/A	N/A	*number*	*number*	*number*	*number*		*Optional*
	% of the number of Recipients included in the aggreate disclosure in the total number, by category, of Recipients disclosed - *Art. 3.02*					N/A	N/A	%	%	%	%		N/A
**HCOs**	***INDIVIDUAL NAMED DISCLOSURE - one line per HCO*** *(i.e. all transfers of value during a year for an individual HCO will be summed up: itemization should be available for the individual Recipient or public authorities' consultation only, as appropriate)*												
	HCO 1					*Yearly amount*	*Yearly amount*	*Yearly amount*	*Yearly amount*	*Yearly amount*	*Yearly amount*		*Optional*
	HCO 2					*Yearly amount*	*Yearly amount*	*Yearly amount*	*Yearly amount*	*Yearly amount*	*Yearly amount*		*Optional*
	etc.					*Yearly amount*	*Yearly amount*	*Yearly amount*	*Yearly amount*	*Yearly amount*	*Yearly amount*		*Optional*
	OTHER, NOT INCLUDED ABOVE - where information cannot be disclosed on an individual basis for legal reasons												
	**Aggregate amount attributable to transfers of value to such Recipients** - *Art. 3.02*					*Aggregate HCOs*	*Aggregate HCOs*	*Aggregate HCOs*	*Aggregate HCOs*	*Aggregate HCOs*	*Aggregate HCOs*		*Optional*
	**Number of Recipients in aggregate disclosure** - *Art. 3.02*					*number*	*number*	*number*	*number*	*number*	*number*		*Optional*
	% of the number of Recipients included in the aggreate disclosure in the total number, by category, of Recipients disclosed - *Art. 3.02*					%	%	%	%	%	%		N/A

**R&D**	***AGGREGATE DISCLOSURE***		
	Transfers of Value re Research & Development as defined - *Article 3.04 and Schedule 1*	*TOTAL AMOUNT*	*OPTIONAL*

latest update: 27 June 2019

Note

The screenshot was taken from version the EFPIA Code which was in force the time of writing.[1]

Part 2: Examples of data reporting not following the EFPIA "disclosure template"

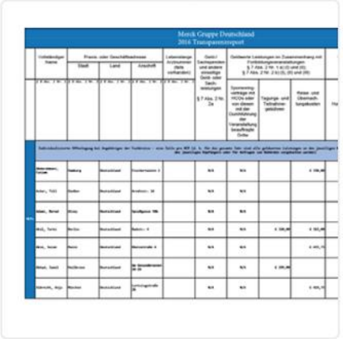

de__merck__2016.pdf
PDF

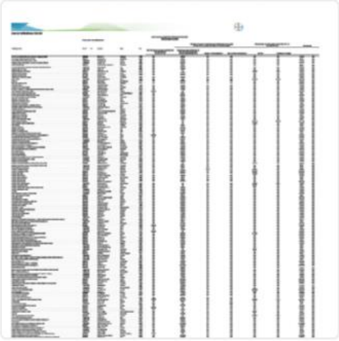

de__bayer__2016.pdf
PDF

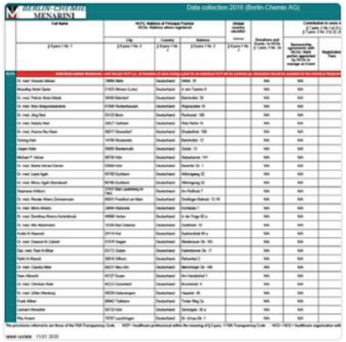

de__berlin-chemie__2016.pdf
PDF

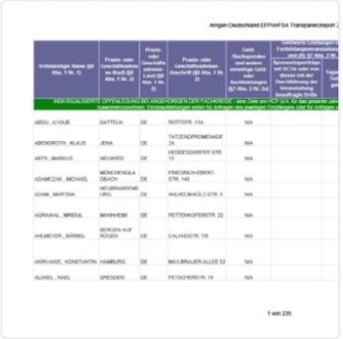

de__amgen__2016.pdf
PDF

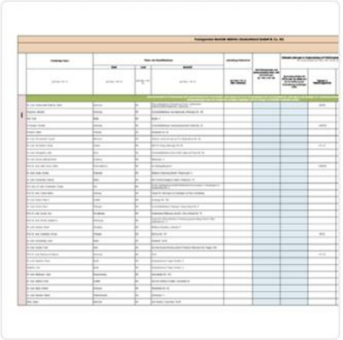

de__abbvie__2016.pdf
PDF

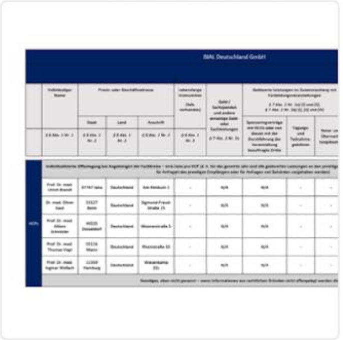

de__bial__2017.pdf
PDF

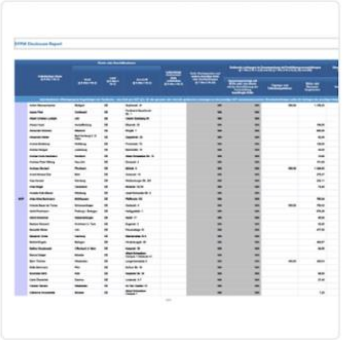

de__biogen__2017.pdf
PDF

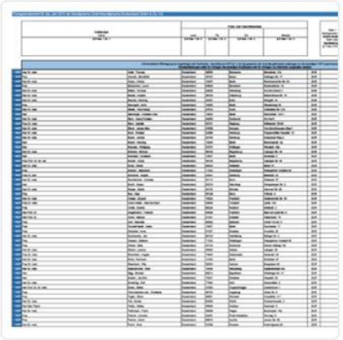

de__mundipharma__2016.pdf
PDF

Note

The screenshots were taken as part of the data curation process involved in creating the eurosfordocs.eu database.[2]

References

1. EFPIA. EFPIA Code of practice 2019 [Available from: https://www.efpia.eu/media/554677/efpia_codes_a5_v3-2021_sm.pdf.
2. Eurosfordocs.eu. Tech documentation, 2021 [Available from: eurosfordocs.eu/on-the-tech-side/tech-documentation/ accessed 4th January 2021.

For peer review only

Online Supplement 4. Approaches to data presentation decreasing the accessibility of payment disclosures

Part A: Techniques used when publishing payment data on individual company websites

1. Publish data as PDF documents (and not csv or XLS files) to make analysis difficult (example: most companies throughout Europe, except where the pharmaceutical industry trade groups created centralised databases).
2. Make the PDF document available only in an online viewer rather than as a separate file to prevent it from being downloaded for analysis (example: Menarini Switzerland).
3. Publish PDF documents consisting of images (and not text) to prevent any searches within the data (e.g. for recipient names) (example: Pfizer Sweden).
4. Reduce the resolution of image-based PDF documents to make them *almost* unreadable without constantly zooming in and out (example: Novo-Nordisk Spain).
5. Create PDF documents with repeated table headers occupying most of each page in the disclosure report. In some cases, if the content is image-based, the resulting PDF can exceed 1800 pages, and over 350 MB, which discourages users from opening or downloading it (example: Novartis Italy).
6. Require users to follow a lengthy process of accepting the "Terms of use" of the disclosed information to discourage engagement with the data (example: Pfizer Spain).

Note: Different techniques can be combined. For example, the disclosure report can be made available only in an online viewer (2), with each page published as an image (3) and in a low resolution (4) (example: Roche Italy).

Part B: Techniques used when publishing payment data in centralised databases.

1. Require users to follow a lengthy process of accepting the "terms of use" of the disclosed information to discourage engaging with the data (example: the Czech Transparentní spolupráce database).
2. Enable searching only for specific recipients, without the possibility of searching for companies or recipient categories (e.g. medical specialties) (the Czech Transparentní spolupráce database).
3. Make searches conditional on obtaining recipient ID numbers from another website (example: the Czech Transparentní spolupráce database)
4. Do not include the possibility of downloading the database as a single file to prevent analysis (Examples: the Irish Transfer of Value database – and all centralised industry platforms except Disclosure UK)

For peer review only

Online supplement 5. Eurosfordocs.eu – database summary (2017-2019)[1]

Country	Disclosure reports	Successfully extracted (parsed) disclosure reports	Parse ratio	Companies associated with parsed disclosure reports	Number of payments to healthcare professionals and organisations	Value of payments (€)[2]
UK	1[3]	1	100%	141	164,112	1,771,785,871
Germany	112	89	79%	32	103,477	1,524,231,568
Spain	60	48	80%	16	370,444	959,704,223
Italy	60	57	95%	19	143,244	954,063,974
Switzerland	138	117	85%	41	36,503	471,638,889
Sweden	184	168	91%	68	15,434	249,913,018
Ireland	1[3]	1	100%	46	18,312	97,259,959
Total	556	481		160	851,526	6,028,597,501

Notes.

[1] – All data is accurate as of January 2021. Eurosfordocs.eu is updated regularly to reflect occasional changes in disclosure reports published by drug companies.

[2] – All payment values in non-euro currencies were converted to euros based the exchange rate obtained from the CurrencyConverter [1] a Python library for exchange rates.

[3] – The UK and Ireland are the only countries reported in the table in which all drug company payments are included in a single database. In all other countries, disclosure reports are published on individual websites for each company.

References

1. CurrencyConverter. CurrencyConverter 0.14.4 2020 [Available from: https://pypi.org/project/CurrencyConverter/ accessed 19th January 2021.

For peer review only

BMJ Open

Accessibility and quality of drug company disclosures of payments to healthcare professionals and organisations in 37 countries: A European policy review

Journal:	*BMJ Open*
Manuscript ID	bmjopen-2021-053138.R2
Article Type:	Original research
Date Submitted by the Author:	29-Oct-2021
Complete List of Authors:	Ozieranski, Piotr; University of Bath, Department of Social and Policy Sciences<br>Martinon, Luc; Euros for Docs<br>Jachiet, Pierre-Alain; Euros for Docs, Department of Social and Policy Sciences<br>Mulinari, Shai; Lunds Universitet, Sociology
**Primary Subject Heading**:	Health policy
Secondary Subject Heading:	Ethics
Keywords:	ETHICS (see Medical Ethics), Health policy < HEALTH SERVICES ADMINISTRATION & MANAGEMENT, Protocols & guidelines < HEALTH SERVICES ADMINISTRATION & MANAGEMENT

SCHOLARONE™
Manuscripts

I, the Submitting Author has the right to grant and does grant on behalf of all authors of the Work (as defined in the below author licence), an exclusive licence and/or a non-exclusive licence for contributions from authors who are: i) UK Crown employees; ii) where BMJ has agreed a CC-BY licence shall apply, and/or iii) in accordance with the terms applicable for US Federal Government officers or employees acting as part of their official duties; on a worldwide, perpetual, irrevocable, royalty-free basis to BMJ Publishing Group Ltd ("BMJ") its licensees and where the relevant Journal is co-owned by BMJ to the co-owners of the Journal, to publish the Work in this journal and any other BMJ products and to exploit all rights, as set out in our licence.

The Submitting Author accepts and understands that any supply made under these terms is made by BMJ to the Submitting Author unless you are acting as an employee on behalf of your employer or a postgraduate student of an affiliated institution which is paying any applicable article publishing charge ("APC") for Open Access articles. Where the Submitting Author wishes to make the Work available on an Open Access basis (and intends to pay the relevant APC), the terms of reuse of such Open Access shall be governed by a Creative Commons licence – details of these licences and which Creative Commons licence will apply to this Work are set out in our licence referred to above.

Other than as permitted in any relevant BMJ Author's Self Archiving Policies, I confirm this Work has not been accepted for publication elsewhere, is not being considered for publication elsewhere and does not duplicate material already published. I confirm all authors consent to publication of this Work and authorise the granting of this licence.

For peer review only

Accessibility and quality of drug company disclosures of payments to healthcare professionals and organisations in 37 countries: A European policy review

For peer review only

Piotr Ozieranski [1]
Luc Martinon[2]
Pierre-Alain Jachiet[2]
Shai Mulinari [3]

[1] Department of Social and Policy Sciences, University of Bath
[2] Euros for Docs
[3] Department of Sociology, Lund University

Correspondence to:
Piotr Ozieranski
Department of Social and Policy Sciences
University of Bath
BA2 7AY
Email: p.ozieranski@bath.ac.uk
Phone: +44 (0) 1225 384204

Key words: pharmaceutical industry, self-regulation, public regulation, transparency, financial disclosure, conflicts of interest

Wordcount: 5388

For peer review only - http://bmjopen.bmj.com/site/about/guidelines.xhtml

Abstract

Objectives: To examine the accessibility and quality of drug company payment data in Europe.

Design: Comparative policy review of payment data in countries with different regulatory approaches to disclosure.

Setting: 37 European countries.

Participants: European Federation of Pharmaceutical Industries and Associations, its trade group and their drug company members; eurosfordocs.eu, an independent database integrating payments disclosed by companies and trade groups; regulatory bodies overseeing payment disclosure.

Main outcome measures: Regulatory approaches to disclosure (self-regulation, public regulation, combination of the two); data accessibility (format, structure, searchability, customisable summary statistics, downloadability) and quality (spectrum of disclosed characteristics, payment aggregation, inclusion of taxes, recipient or donor identifiers).

Results: Of 30 countries with self-regulation five had centralised databases, with Disclosure UK displaying the highest accessibility and quality. In 23 of the remaining countries with self-regulation and available data, disclosures were published as PDFs on individual company websites, preventing the public from understanding payment patterns. Eurosfordocs.eu had greater accessibility than any industry-run database, but the match between of the value of payments integrated in eurosfordocs.eu and summarised separately by industry in seven countries ranged between 56%-100% depending on country. Eurosfordocs.eu shared quality shortcomings with the underlying industry data, including ambiguities in identifying payments and their recipients. Public regulation was found in 15 countries, used either alone (3), in combination (4) or in parallel with (8) self-regulation. Of these countries, 13 established centralised databases with widely ranging accessibility and quality and sharing some shortcomings with the industry-run databases. The French database, Transparence Santé, had the highest accessibility and quality, exceeding that of Disclosure UK.

Conclusions: The accessibility and quality of payment data disclosed in European countries are typically low, hindering investigation of financial conflicts of interest. Some improvements are straightforward but reaching the standards characterising the widely researched US Open Payments database requires major regulatory change.

Strengths and limitations

- We investigate the quality and accessibility of drug company payment disclosure data in 37 European countries.
- We use a set of measures relevant for countries with industry self-regulation, public regulation, and a combination of the two.
- We present our results as a “heat map” showing the least and most problematic aspects of payment data accessibility and quality.

For peer review only

- One key limitation is that that we did not quantify some aspects of the accessibility and quality of payment data.

For peer review only

1. Introduction

Financial conflicts of interest (FCOIs) can bias healthcare research, practice, education, and policy.[1-3] The last decade has seen a global trend towards addressing concerns about FCOIs by publishing drug company payments to the healthcare sector.[4-8] It is best exemplified by the US Sunshine Act, establishing Open Payments, a database triggering extensive research on payment distribution,[9 10] and its links with drug prescription[11] and cost.[12 13] Open Payments increases transparency of FCOIs by enabling cross-checking information collected by professional organisations,[14] conference organisers,[15] and scientific journals.[16] It also aids identifying corruption by highlighting unusual payment patterns.[17 18]

Unlike the US, in most European countries drug company payments are disclosed via industry self-regulation.[4 6] In Europe, the prevalent form of self-regulation draws on the Code of Practice of the European Federation of Pharmaceutical Industries and Associations (EFPIA), with its minimum requirements transposed into the codes of EFPIA's national trade group members.[19] Self-regulation allows the industry to develop, implement, and oversee the rules of payment disclosure.[4 20] Compared to the US Sunshine Act, one key shortcoming of self-regulation, resulting from the industry's interpretation of European privacy laws, is making company disclosures conditional on consent granted by payment recipients.[21-23] Other problems include broader, and therefore difficult to interpret, payment categories (grants and donations, contributions to costs of events, fees for service and consultancy),[22] which are also fewer than in the US, excluding royalties, ownership and investments. Additionally, research payments are only disclosed as lump sums per company without named recipients.[5 24] One advantage of self-regulation is a greater scope of covered healthcare professionals, including not only physicians but also nurses (to be included in the US starting from 2022[25]), pharmacists, and others. [5 21] Further, self-regulation includes, like in the US, hospital recipients of payments but also general practice surgeries, professional associations, and other healthcare organisations.[5 24]

Only few European countries, including France, Portugal and Latvia, use government regulation, principally legislation, to impose disclosure requirements for donors and recipients, including mandatory disclosure.[4 6] Finally, one country, the Netherlands, has been identified as using a combination of self- and public regulation, with the disclosure regulations developed with government's input, lacking a legal basis and enforced via self-regulation.[4]

The scrutiny of European payment data has been limited, except for case studies of payment distribution in the UK,[21 24] Germany,[26] and Ireland,[27] and a comparative analysis of payments shares not disclosed by recipients in the UK, Germany, Sweden, Switzerland, Italy, Ireland, and Spain.[28] However, France is the sole country where relationships between payments and prescribing have been investigated.[29] Similarly, the potential for detecting organisational-level FCOIs is unrealised, with only two studies examining discrepancies in payments reported separately by companies and some healthcare providers[30] and commissioners[31] in England. Further, corrupt relationships identified via official investigations pertaining to Greece,[32] Poland and Russia[33] might have been revealed earlier by examining payment patterns, following the US' example.[17 18] Therefore, the evidence

For peer review only

base for any policy reform is thin, leaving the industry as the only stakeholder likely to have in-depth understanding of payment data, particularly in countries with self-regulation.

The likely reasons behind the scant disclosure research are the low accessibility and quality of payment data. Regarding accessibility, a study of European disclosure approaches has found that of six countries with self-regulation five lacked centralised payment databases.[4] In one of these countries, Germany, the dispersal of disclosures on drug company websites was a major obstacle in data analysis.[19 26 28] A recent remedial initiative by activist data scientists has involved creating a database called eurosfordocs.eu. Inspired by a similar German project,[26] eurosfordocs.eu integrates data disclosed separately by many companies in countries with self-regulation.[28 34] Contrastingly, of the four countries identified as having government regulation or combining it with self-regulation three had databases integrating payments reported by all companies.[4]

A related aspect of low accessibility both in countries with self-regulation and government regulation is poor user interface.[4] Of the six studied countries with self-regulation only Disclosure UK, the database run by the Association of the British Pharmaceutical Industry (ABPI), was judged as user friendly.[4] However, of the three databases in countries using government regulation or combining it with self-regulation the Dutch and Portuguese databases were described as “partially” user friendly, while the French was deemed “not” user friendly.[4] Challenges in the interface of the French database were only addressed by the independent data platform eurosfordocs.fr, stimulating journalistic investigations into FCOIs.[35-37]

The second problem, payment data quality, has only been examined in countries with self-regulation. For example, analyses of Disclosure UK revealed inconsistencies in reporting of payment values and recipients,[21 24] compounded by the absence of unique recipient identifiers.[38] Similar shortcomings, including duplicate entries, were found in Germany,[26] indicating that they might characterise self-regulation more broadly.

Therefore, important gaps exist in our understanding of the accessibility and quality of European payment data. First, ongoing debates on the introduction of public regulation in some countries[5] suggest that the only comprehensive European regulatory overview[6] might have missed key regulatory developments, potentially with implications for data accessibility and quality.

Second, the implementation of the requirements of the EFPIA Code[19] has not been fully scrutinised. For example, although some trade groups will only meet the minimum standards (e.g. by expecting companies to publish data on their websites), others might exceed them (e.g. by creating centralised databases).[4 28] The need for establishing a comprehensive pattern of compliance is underscored by findings from Sweden and the UK suggesting failure of self-regulation of drug marketing to meet some of its own key promises.[20 39]

Third, regulatory approaches in many European countries have escaped scrutiny,[4] making it unclear whether payment data reported in these countries shares the strengths and weaknesses identified elsewhere. Consequently, although some aspects of government

For peer review only - http://bmjopen.bmj.com/site/about/guidelines.xhtml

regulation, such as a greater scope of covered industries, have been demonstrated as superior to self-regulation,[4 6] it remains uncertain whether this is reflected by payment data accessibility or quality.[4]

Finally, the to-date evaluative criteria need refinement, as some, such as "user friendliness" have attracted a contrasting appraisal of the same disclosure database by different expert commentators.[4 22]

We have two objectives. First, to identify regulatory approaches to payment disclosure in Europe. Second, to examine the accessibility and quality of payment data disclosed in countries with different approaches to disclosure.

2. Methods

2.1. Data collection

2.1.1. Identification of regulatory approaches

To identify regulatory approaches to payment disclosure in Europe, PO and LM identified available peer-reviewed English-language research on the regulation of drug company payment disclosure. We searched Scopus using the terms "Sunshine Act", "Open Payments", as well as "European Federation of Pharmaceutical Industries and Associations" and "EFPIA" combined with "disclosure". We applied the same terms in the Google search engine to identify "grey literature", including non-peer reviewed reports.

Subsequently, PO and LM conducted iterative searches on websites dedicated to industry payment disclosure, including EFPIA's website and its national trade group members' websites. We also examined the country profiles published by MediSpend[40] and the websites of four major companies with presence across Europe (Amgen, GSK, Merck Serono, and Bayer) providing access to company disclosure methodologies which reflect local regulatory requirements. Finally, we considered the websites of public or multistakeholder bodies which the previous steps identified as involved in overseeing payment disclosure.

Finally, PO surveyed industry trade groups and public or multistakeholder bodies overseeing payment disclosure (Online Supplement 1). The first round of standardised questions was emailed in mid-November 2020, followed up by reminder messages in late December 2020, asking recipients to provide answers by the end of the 1st week of January 2021. Of 34 approached pharmaceutical trade groups 17 replied. Of those, 14 answered at least some of the questions, while the remaining ones sent holding messages. Of 13 approached public or public or multistakeholder bodies ten replied. Of those, six answered at least some of the questions, three sent holding messages, and one redirected us to another institution (Online Supplement 2).

For peer review only - http://bmjopen.bmj.com/site/about/guidelines.xhtml

2.1.2. Data on accessibility and quality of payment disclosures

First, in countries with self-regulation, we considered industry codes, reports, press releases, trade group websites, and industry-run databases. Second, LM and PAJ recorded their observations regarding the format and structure of payment data when designing scripts for scraping company and trade group websites to be integrated in eurosfordocs.eu.[28] Third, in countries with disclosure overseen by public or multistakeholder bodies the data included relevant legislation, the websites of bodies managing payment disclosure, and disclosure databases. Fourth, in both countries with self-regulation and public regulation we considered responses from our stakeholder survey. Finally, in countries with self-regulation and covered by eurosfordocs.eu we collected – for verification purposes – national-level summary statistics published by EFPIA, industry trade groups, and survey responses from the trade groups.

2.2. Data analysis

2.2.1. Content analysis

Most of the source material was available in English. If this was not the case, we used Google Translate and Deepl.com, clarifying any linguistic issues by cross-checking with other online sources and consulting with relevant national bodies and colleagues with language expertise.

We coded the regulatory approaches deductively building on an earlier categorisation which distinguished countries with self-regulation, government regulation, and a combination of the two.[4] We modified it by considering new regulatory developments, such as the 2016 decision by the Spanish Data Protection Agency[41] making disclosure by healthcare professionals compulsory without new government regulation.[28] Therefore, we replaced "government regulation" with "public regulation", comprising "government regulation", i.e. legislation relating directly to payment disclosure, and "regulatory intervention", i.e. decisions by data protection agencies clarifying the rules of payment disclosure based on other existing legislation.

Deductive codes relating to data accessibility and quality were developed using earlier research.[4 5 24] Inductive coding was applied to the types of disclosed information and company techniques of decreasing data accessibility, which were identified when integrating industry data within eurosfordocs.eu

The data was coded by PO and results were validated by team discussions, resolving any differences by agreement. In analysing industry-self regulation, we set the characteristics of disclosed data against recommendations from the EFPIA Code. Similar comparison was not necessary in relation public regulation as it does not introduce any optionality.

2.2.2. Descriptive statistical analysis

For peer review only - http://bmjopen.bmj.com/site/about/guidelines.xhtml

As eurosfordocs.eu involved data extraction using disclosures published by individual companies and industry trade groups, we estimated the match between the database and the underlying data by comparing the value of payments calculated in specific countries using eurosfordocs.eu with national-level summaries obtained from industry sources.

2.2.3. Outcome measures

We had one primary outcome measure identifying the regulatory approaches to payment disclosure in each country – self-regulation, public regulation and a combination of the two. As we identified both self-regulation and public regulation in some countries, we noted the number of regulatory approaches in each country – single (only self-regulation, public regulation, or a combination of the two) or two (self-regulation and public regulation used in parallel).

In countries with self-regulation, we recorded whether it was based on the EFPIA Code, including shared payment, donor, and recipient categories, or involved a distinct national industry code. For countries following the EFPIA Code, we specified whether trade groups were obliged to do so as EFPIA members or did this voluntarily as non-members.

Considering countries with public regulation, we distinguished those using government regulation, regulatory intervention, or both. In countries with government regulation, we distinguished those introducing bespoke legislation focusing on payment disclosure or incorporating new provisions into existing pharmaceutical or medical device legislation. In countries where public-and self-regulation were used in parallel, we recorded whether any overlap existed between the donors, recipients and payments covered by each approach.

In countries combining self-and public regulation, we denoted the form of both self-regulation and public regulation and how they were integrated.

The measures of accessibility and quality reflected the heterogeneity of payment data presentation. The basic measure of accessibility applied in all countries was whether it was disclosed on a centralised database or multiple websites. In addition, for countries with centralised databases, we created a "heat map" aiding data synthesis and interpretation (Table 1).

For peer review only

Table 1 Heat map of measures of accessibility and quality of payment databases.

		**Measures of payment data accessibility**		
		**Higher accessibility**	**□--------------□**	**Lower accessibility**
Database format	How is the database published (i.e. PDF, XLS, CSV, webpage)?	Webpage, XLS or CSV	Readable PDFs	Image-based PDFs
Database structure	Does the data from all companies follow a single template consistently?	Yes	N/A	No
Database searchability	Can the database be searched? If so, can database searches be carried out without data users providing any additional information?	Yes	Database searchable but additional information needed for searches	No
Customisable summary statistics	Does the database offer users the possibility of generating real-time, dynamic data summaries based on selected database characteristics?	Yes	N/A	No
Downloadability	Can the database be downloaded (e.g. as a single CSV or XLS file) for further analysis?	Yes	N/A	No
		**Measures of payment data quality**		
		**Higher quality**	**□--------------□**	**Lower quality**
Spectrum of disclosed characteristics	What characteristics are included in relation to donors, recipients, and payments?	All characteristics from the EFPIA disclosure template covered as well as some additional ones	All characteristics from the EFPIA disclosure template covered	At least some characteristics from the EFPIA disclosure template not covered, including instances where some additional characteristics are provided
Aggregation of payments	Are payments itemised (i.e. all payments have separate entries) or are they aggregated on an annual basis (e.g. per recipient and/or payment category)?	All payments itemised	Some payments itemised, other s aggregated	All payments aggregated
Inclusion of taxes	Is it clear whether payments are reported inclusive or exclusive of any taxes, such as VAT?	Single rule for all companies and payments	No single rule, each company sets its own rules for VAT reporting which are published separately from payment disclosures[1]	Rules around tax reporting are unclear
Unique identifiers	Do reported donors (drug companies) or recipients (healthcare professionals or organisations) have unique identifiers?	All donors and recipients	Some donors or recipients	No unique identifiers

Notes

[1] – The EFPIA Code stipulates that companies must publish documents, called "methodological notes", which should explain their approach to reporting VAT and other taxes. Companies publish these documents separately from payment disclosures but consulting them is necessary to understand, compare, and aggregate payment values.

For peer review only - http://bmjopen.bmj.com/site/about/guidelines.xhtml

On top of the measures included in Table 1 we had one additional measure of quality for eurosfordocs.eu as a database derived from payment disclosures published by drug companies and industry trade groups. We estimated the comprehensiveness of data extraction by comparing the value of payments available in eurosfordocs.eu with those reported separately in national-level industry data summaries. We set three arbitrary levels of match – exact (no difference between eurosfordocs.eu and summary industry data), close (difference between eursofrdocs.eu and industry data worth less than 10% of summary industry data) and low (difference exceeding 10% of summary industry data).

Finally, in countries with self-regulation but without centralised databases we examined whether industry trade groups created gateways leading to disclosure documents, as recommended by EFPIA.[19] To illustrate challenges in data accessibility we also generated lists of examples of, first, deviations from the EFPIA-recommended data presentation format ("EFPIA disclosure template"[19]); and, second, the ways of presenting data which decreased its accessibility.

2.3. Ethics

This study did not require a full ethics approval as no individual payment data was processed. Its ethical implications were approved via a peer ethics review process at the Department of Social and Policy Sciences, University of Bath in April 2016.

2.4. Patient and public involvement

We did not involve patient groups or the public. Our policy recommendations seek to increase public engagement with payment data by enhancing its accessibility and quality.

3. Results

We first map the regulatory approaches to payment disclosure in Europe. We then examine the accessibility and quality of payment data published by pharmaceutical companies and trade groups in countries with self-regulation. Subsequently, we focus on industry data in the subset of countries with self-regulation and covered by eurosfordocs.eu. Finally, we analyse payment data in countries with public regulation or combining public regulation with industry self-regulation.

3.1. Mapping European regulatory approaches to payment disclosure

Before analysing the accessibility and quality of industry payment data we must describe how it is disclosed in each European country (Box 1).

Box 1. Approaches to regulating payment disclosure in European countries

Country[1]	Regulatory approaches to payment disclosure: Self-regulation	Public regulation	Combination of self- and public regulation
AUSTRIA	✓		
BOSNIA AND HERZEGOVINA	✓		
BULGARIA	✓		
CROATIA	✓		
CYPRUS	✓		
CZECH REPUBLIC	✓		
GERMANY	✓		
ICELAND	✓		
IRELAND	✓		
ITALY	✓		
LUXEMBOURG	✓		
NORTH MACEDONIA	✓		
MALTA	✓		
NORWAY	✓		
POLAND	✓		
RUSSIA	✓		
SERBIA	✓		
SLOVENIA	✓		
SWEDEN	✓		
SWITZERLAND	✓		
UK	✓		
UKRAINE	✓		
DENMARK	✓	✓	
ESTONIA	✓	✓	
GREECE	✓	✓	
HUNGARY	✓	✓	
LATVIA	✓	✓	
LITHUANIA	✓	✓	
ROMANIA	✓	✓	
SLOVAKIA	✓	✓	
FRANCE		✓	
PORTUGAL		✓	
TURKEY		✓	
BELGIUM			✓
FINLAND			✓
THE NETHERLANDS			✓

For peer review only - http://bmjopen.bmj.com/site/about/guidelines.xhtml

SPAIN			✓
n = 37	n = 30	n = 11	n = 4

Notes

[1] – Excluded countries: Albania, Andorra, Belarus, Lichtenstein, Monaco, Montenegro, San Marino, and Vatican City.

For peer review only

We identified self-regulation in 30 countries in the form of codes issued and overseen by industry trade groups.[19] In 28 of those, the industry codes incorporate the provisions of the EFPIA Code[19 42] as a necessary requirement of trade groups membership in EFPIA. This makes self-regulation the "default approach" to payment disclosure in Europe, with EFPIA holding power to exempt certain countries from following its Code.[43] The first exception is Luxembourg. While the Luxembourgish trade group is not an EFPIA member, it decides voluntarily to implement the regulation of payment disclosure modelled on the EFPIA Code.[44] The second exception is Denmark. Although the Danish trade group is an EFPIA member, EFPIA exempts Denmark from following its Code, given the country's separate public regulation provisions.[43] As the public regulation of payment disclosure in Denmark covers only healthcare professionals,[45] the Danish pharmaceutical trade group developed a code covering only "grants and donations" to hospitals.[46]

We found public regulation in 11 countries. In all cases, it takes the form of government regulation, in which provisions relating to payment disclosure are included either in bespoke new legislation (France, Lithuania, and Romania) or are incorporated into existing pharmaceutical legislation (the remaining countries). In addition, in Greece, the Data Protection Agency made a regulatory intervention by issuing an interpretation of the government regulation.[47]

Only in France, Portugal and Turkey public regulation is the sole regulatory approach, replacing self-regulation entirely. EFPIA excepted France and Portugal from applying the EFPIA Code considering the nature of their public regulation; [43] however, the implementation of the EFPIA Code in Turkey is only suspended while its compatibility with the EFPIA Code is being reviewed.[48]

In the remaining 8 countries with public regulation, there is also parallel self-regulation. In 4 of these (Denmark, Lithuania, Romania, and Slovakia), self- and public regulation cover different donors, payments, or recipients, whereas in the remaining ones (Estonia, Greece, Hungary, and Latvia) donors, recipients and payments disclosed via public and self-regulation may overlap. Consequently, the existence of parallel self-and public regulation in the 8 countries means that self-regulation is used exclusively in 22 of the 30 countries with this approach.

Self- and public regulation are combined as a single approach in four countries. Contrasting with countries with public regulation, here the industry contributes to managing payment disclosure. However, unlike in countries with self-regulation, the industry derives at least some of its regulatory power from public authorities, often sharing it with other stakeholders. In two of the four countries, public regulation takes the form of government regulation (Belgium and Finland) and, and in the two others – regulatory intervention (Spain and the Netherlands).

Belgium regulates payment disclosure via a bespoke "Sunshine Act" but the interpretation of its key provisions is left to betransparent.be, a multi-stakeholder body involving industry and professional organisations,[49 50] which also runs the Transparency Register integrating company disclosures.[51 52] In Finland, new provisions have been introduced into the Medicines Act, stipulating that drug companies "must keep available for public review" a list

For peer review only

of all payments to “associations in the fields of medicine and health care”[53] but in practice the disclosure takes place following the EFPIA Code.

In Spain, public regulation involves an intervention by the Data Protection Authority[41] confirming that the publication of named payment recipients does not require recipient consent.[28] However, like in Belgium and Finland, disclosure is managed by companies based on the EFPIA Code. In the Netherlands, payments are disclosed using self-regulatory rules developed by the Foundation for the Code for Pharmaceutical Advertising, which are separate from the EFPIA Code. Like in Belgium, the central platform is a multistakeholder body involving the industry and healthcare providers.[54] However, public authorities triggered the policy debate on payment disclosure and, having considered self-regulation preferable to public regulation, they lent it financial support and monitor its performance.[55] [56] Further, consistent with the regulatory intervention in Spain, the Dutch Data Protection Authority confirmed that recipient consent is not required.[56]

3.2. Data disclosed via self-regulation by pharmaceutical companies and trade groups

We were able to collect information on accessibility and quality of payment data in 28 of the 30 countries with self-regulation.

Regarding data accessibility, the EFPIA Code allows companies within each country to disclose payments either on a centralised platform or individual websites.[19] However, only five trade groups have established databases for all companies, including four countries following the EFPIA Code and one using its own Code (Danish Association of the Pharmaceutical Industry, LIF). Of the five industry-run databases none had customisable summary statistics (Table 2). Moreover, only one was fully searchable (i.e. without additional information required for searches) and just two were downloadable. Overall, Disclosure UK had by far the highest data accessibility.

Turning to data quality, only the Czech database used unique donor and recipient identifiers consistently, but, because they were required for searches, they paradoxically decreased data accessibility. The second most frequent problem across the databases was tax reporting. While in the four databases established under the EFPIA Code the rules on tax reporting might be reconstructed using "methodological notes" published separately by each company,[19] the Danish database had no information regarding tax. Taken altogether, Disclosure UK had the highest data quality, although here it was more closely matched by the Czech database.

For peer review only - http://bmjopen.bmj.com/site/about/guidelines.xhtml

Table 2 Accessibility and quality of drug company payment data disclosed via centralised industry databases and eurosfordocs.eu

			Payment data accessibility[1]					Payment data quality[1]			
**Country**	**Name of regulation**	**Overseeing authority and database web link [2, 3, 4]**	**Document format**	**Single data template**	**Database searchable**	**Customisable summary statistics**	**Database downloadable**	**Characteristics included**	**Aggregation of payments**	**Payments with or without taxes**	**Unique identifiers**
**Self-regulation at the European level – minimum requirements**											
EFPIA	EFPIA Code	EFPIA	Not regulated	Yes ("EFPIA disclosure template"), but deviations allowed	Not regulated	Not regulated	Not regulated	Donors; recipients; recipient location; payment categories and amounts; year	Annually per payment type	No single rule, each company sets its own rules for VAT reporting which are published separately from payment disclosures	Optional
**Centralised online industry databases**											
UNITED KINGDOM	ABPI Code of Practice	Association of the British Pharmaceutical Industry	Website, XLS	Yes	Yes	No	Yes	Donors; recipients; recipient categories (healthcare professionals) and location; payment categories and amounts; year; web links with further descriptions for some payments	Annually per payment type for healthcare professionals; payments to healthcare organisations itemised	No single rule, each company sets its own rules for VAT reporting which are published separately from payment disclosures	No
CZECH REPUBLIC	Eticky Kodex AIPF	Asociace inovativního farmaceutického průmyslu	Website	Yes	Yes (but requires donor or recipient identifiers)	No	No	Donors; donor location; recipients; recipient location; payment categories and amounts; year	Annually per payment type	No single rule, each company sets its own rules for VAT reporting which are published separately from payment disclosures	Recipient and donor identifiers
DENMARK	Ethical rules for the pharmaceutical industry's donations and grants	Lægemiddelindustriforeningen	Readable PDFs	Yes	No	No	Yes	Donors; project name; recipients; product name; funded activity; payment goal; timescale of funded activity; payment amount and form (cash or benefit in kind)	No	Unclear	No

For peer review only - http://bmjopen.bmj.com/site/about/guidelines.xhtml

GREECE	SFEE Code of Conduct	Hellenic Association of Pharmaceutical Companies	Website	Yes	No	No	No	Donors; recipients; recipient categories; payment descriptions, categories goals, and amounts; date	No	No single rule, each company sets its own rules for VAT reporting which are published separately from payment disclosures	No
IRELAND	Code of Practice of the Pharmaceutical Industry	Irish Pharmaceutical Healthcare Association	Website	Yes	No	No	No	Donors; recipients; recipient location; payment categories and amounts; year	Annually per payment type	No single rule, each company sets its own rules for VAT reporting which are published separately from payment disclosures	Partial (recipient identifiers used by some companies)
**Industry data integrated within an independent database[2]**											
EUROS FORDOCS. EU	Codes of conduct in countries where data was collected[5]	N/A	Website, XLS	Yes	Yes	Yes	Yes	Donors; recipients; recipient location; payment categories and amounts; year	Annually per payment type for healthcare professionals (all countries); Annually per payment type for healthcare organisations in all countries but the UK, where payments to healthcare organisations are itemised	No single rule, each company sets its own rules for VAT reporting which are published separately from payment disclosures	Spain: recipient identifiers; Other countries: No

Notes

[1] – Lighter colours indicate, respectively, higher, and darker colours – lower, data accessibility and quality. In the upper part of the table, the centralised industry databases are presented in the descending order of their overall data accessibility and quality, that is, the greater overall number of lighter cells a database has the higher its position within the table. Databases with equal numbers of lighter and darker cells are sorted alphabetically.

[2] – The disclosure requirements ordinarily cover both healthcare professionals and organisations. The exceptions are the database run by the Danish pharmaceutical industry trade group (donations to hospitals) and the database run by the Greek pharmaceutical industry trade group (only payments to healthcare organisations).

[3] – Web links are accurate as of May 2021.

[4] – Some pharmaceutical industry trade groups create and delegate some responsibility for the everyday operation of their codes to sub-divisions such as the Ethical Committee for the Pharmaceutical Industry (established by Denmark's LIF) or the Prescription Medicines Code of Practice Authority (established by the UK's ABPI). However, the ultimate responsibility for managing and overseeing the codes is with the trade group.

[5] – Ireland, Italy, Germany, Spain, Sweden, Switzerland, and the UK.

For peer review only - http://bmjopen.bmj.com/site/about/guidelines.xhtml

In 23 of the remaining countries with self-regulation and available data, disclosures were published on individual websites for each company. Of these, in 18 countries, trade groups had the EFPIA-recommended gateways to these websites.[19 23] Nevertheless, without EFPIA's explicit guidance on the electronic format of disclosure documents disclosures published on company websites in countries with and without gateways were typically PDFs. While some of these documents were "readable", allowing for copying and pasting of information, they offered limited possibilities for efficient searches and integrating data from different companies. Additionally, some companies presented data without strictly following the "EFPIA disclosure template"[19], which further impeded possibilities for cross-company comparisons (Online Supplement 3 has examples of these deviations). Some firms apparently manipulated data presentation using low-resolution, image-based PDFs, which prevented any searches (Online Supplement 4 summarises these techniques).

Given the low accessibility of payment data, analysing its quality was practically impossible in countries without centralised databases. Therefore, we do this using eurosfordocs.eu, a database covering drug company disclosures in countries with self-regulation (Ireland, Italy, Germany, Sweden, Switzerland, and the UK); in this part of the analysis, we also include Spain, a country with a combination of self-and public regulation as it helps illustrate problems characteristic of self-regulation.

3.3. Industry data disclosed via self-regulation and integrated within eurosfordocs.eu

Eurosfordocs.eu had data accessibility superior to all industry-run databases (Table 2). While the Irish and UK databases were also searchable, eurosfordocs.eu offered customisable queries using combinations of donor and recipient names and payment categories. [57] It was the only database offering customisable summary statistics enhancing data exploration. In addition, only eursofordos.eu and Disclosure UK were downloadable for further analysis.

A specific consideration regarding data is estimating how closely eurosfordocs.eu matches the underlying industry disclosures (Table 3). Complete data extraction was only possible in the UK and Ireland, the two countries with centralised trade group databases (Online Supplement 5 summarises the data extraction statistics). Elsewhere data scraping prioritised the 20 largest donors known from the countries with complete data; more data was scraped whenever allowed by formats used by companies.[28] For four of the six countries, the resulting dataset closely or exactly matched the industry's summary country-level data. The two countries with a low match were Germany and Spain, given a high proportion of image-based PDFs hindering data extraction.[28]

For peer review only - http://bmjopen.bmj.com/site/about/guidelines.xhtml

For peer review only

Table 3 Estimation of the comprehensiveness of industry payment data extracted for eurosfordocs.eu (2019)

Country[1]	Total value of payments reported in summary industry data (€m)[2, 3]	Total value of payments extracted to eurosfordocs.eu (€m)[4, 5]	Difference (€m)	Difference as a share of summary industry data (%)[6]	Level of match between summary industry data and eurosfordocs.eu
GERMANY	629	499	130	21%	Low
IRELAND	35	35	0	0%	Exact
SWEDEN	90	82	8	9%	Close
SWITZERLAND	167	155	12	7%	Close
SPAIN	601	337	264	44%	Low
UK	619	611	8	1%[5]	Exact/Close

[1] – Only countries covered by both eurosfordocs.eu and available national-level summary data generated by industry trade groups are included.

[2] – Sources of national-level summary payment data.

- Germany,[58] Spain,[59] Switzerland[60] – publicly available pharmaceutical industry summary data published by the pharmaceutical industry trade groups.
- Ireland – a combination of an Europe-wide report published by EFPIA[61] and email communication with the Irish pharmaceutical industry trade group.[62]
- Sweden – email communication with the pharmaceutical industry trade group.
- The UK – calculations based on data obtained from Disclosure UK, the centralised database of industry payments run by the Association of the British Pharmaceutical Industry.[63]

[3] – All payment values in non-euro currencies were converted to euros based on the average yearly exchanged rates published by the European Central Bank.

[4] – The source of payment values reported in this column are centralised pharmaceutical industry payment databases (Ireland and the UK) and payment reports covering payments made by individual companies (Germany, Spain, Sweden, and Switzerland).

[5] – All payment values in non-euro currencies were converted to euros based the exchange rate obtained from the CurrencyConverter,[64] a Python library for exchange rates.

[6] – Some of the difference between the value of payments based on summary industry data and extracted to eurosfordocs.eu results from the differences in the exchange rates. This is exemplified by the examples of Ireland (both values in euro, no difference) and the UK (original values in the sterling, the difference is caused by different exchange rates used to convert the sterling to euro). By contrast, the 1% difference between eurosfordoscs.eu and Disclosure UK results from two marginally different exchange rates used to convert the sterling to euros

For peer review only

Nevertheless, other aspects of the data quality in eurosfordocs.eu share key limitations with the underlying company disclosures.

First is a narrow spectrum of reported recipient, donor, and payment characteristics. Eurosfordocs.eu does not present payment distribution within the healthcare system due to the incoherent use or omission of recipient categories by drug companies. Of all countries covered by eurosfordocs.eu the UK is the only one where the industry trade group categorised healthcare professionals receiving payments,[65] albeit incoherently;[21] healthcare organisations were nowhere categorised.

Second, consistent with the EFPIA Code[19] payments to healthcare professionals are not itemised but aggregated annually per recipient within each payment category. The same applies to payments to healthcare organisations, except for the UK, where the ABPI mandates that payments to healthcare organisations be itemised.[65] This UK-specific rule might explain the large difference in the number of payments reported with Germany, a country with a similar overall value of payments (Online Supplement 5). However, it is equally possible that not all companies in the remaining six countries covered by the database aggregate payments consistently as some list more than one payment per recipient, which might also indicate that although these recipients have the same names, they are different entities.

Third, the reported payment values must be interpreted cautiously as it is unclear whether they include taxes without consulting the separately published “methodological notes”.[19] Some companies have different approaches to tax reporting depending on payment or recipient categories. Consequently, establishing the value of payments made by each company requires additional forensic work.[24]

Finally, while EFPIA introduces the option of unique recipient identifiers in disclosed payment data,[19] of the seven countries covered by eurosfordocs.eu only the Spanish trade group followed this recommendation. Elsewhere the number of recipients per company and, consequently, the value of payments per recipient remains unknown. Given inconsistent naming approaches in disclosures made by the same or different companies, the same recipient can have different names, and, conversely, different recipients may have the same name.[24] Further, the same recipient can be identified at different levels of aggregation (e.g. hospital wards, departments or hospitals), with self-regulation at least in some countries placing the onus of identifying possible multiple records on payment recipients and not companies.[24 66] Lastly, without identifiers payment data cannot be connected to other databases.

3.4. Data disclosed via public regulation or a combination of public and self-regulation

Having examined countries with self-regulation, we proceed to those with public regulation or a combination of public and self-regulation.

For peer review only - http://bmjopen.bmj.com/site/about/guidelines.xhtml

Of the 15 countries with public regulation or a combination of self-and public regulation, all but two had centralised databases. The exceptions were Finland and Spain, where disclosures were made on individual drug company websites, consistent with the EFPIA Code. Of the thirteen countries with centralised databases, one had a database which was not publicly available (Turkey) and two others had separate databases for different payment categories (Denmark) and healthcare professionals and organisations (Greece). As the information included in the separate Danish and Greek databases did not differ according to our outcome measures, we consider them jointly (Table 4).

For peer review only

Table 4 Accessibility and quality of drug company payment data disclosed via public regulation or a combination of self-regulation and public regulation

Country	Name of regulation	Overseeing authority and database web link [2, 3, 4]	Payment data accessibility[1]					Payment data quality[1]			
			Document format	Single data template	Database searchable	Customisable summary statistics	Database downloadable	Characteristics included	Aggregation of payments	Payments with or without taxes	Unique identifiers
FRANCE	Law No. 2011-2012 (Law Bertrand)	Ministry of Social Affairs and Health	Webpage	Yes	Yes	No	Yes	Donors; donor categories; recipients; recipient categories; payment categories and amounts; date; recipient address	No	Inclusive of VAT	Donors (multiple entries for subsidiaries), recipients (partial)
LATVIA	Regulation No. 378 (2014)	Health Inspectorate	XLS	Yes	No	No	Yes	Donors; recipients; recipient categories; payment name, description, category and amount; date; recipient address	No	Unclear	Donors, recipients
BELGIUM	Sunshine Act of 2016	Federal Agency for Medicines and Health Products	Webpage	Yes	Yes	No	No	Donors; recipients; recipient categories; payment categories and amounts; recipient address; years	Annually per payment type	Unclear	Donors, recipients
LITHUANIA	Law on Pharmacy (provisions from 2019), Ministerial Order No. V-1537 (2020)	State Medicines Control Agency	XLS	Yes	No	No	Yes	Donors; recipients; recipient categories; payment name; date; recipient address	No	Unclear	Donors (not publicly available), recipients (publicly available)
PORTUGAL	Decree Law 20/2013 and 128/2013	National Authority of Medicines and Health Products	Webpage	Yes	Yes	No	No	Donors; donor categories; recipients; payment descriptions and amounts; years	No	Inclusive of VAT	No
ROMANIA	Orders of the Minister of Health 194/2015 and 874/2015	National Agency for Medicines and Medical Devices	Webpage	Yes	Yes	No	No	Donors; recipients; recipient categories; payment descriptions, categories, and amounts; recipient address; date	No	Unclear	No
SLOVAKIA	Act No. 362/2011 on Medicines and Medical Devices	National Health Information Center	XLS	Yes	No	No	Yes	Donors; recipients; recipient categories (only healthcare professionals); payment descriptions, categories and amounts; clinical trial numbers; product names; recipient address; date	No	Unclear	No
DENMARK	Health Act of 2014, Executive Order No. 1153	Danish Medicines Agency 1) conferences abroad ; 2) professional affiliations	Webpage	Yes	Yes	No	No	Conferences abroad – donors; recipients; recipient categories; recipient address; Professional affiliations – donors; recipients; recipient categories; recipient address; payment amounts	Annually per payment type	Unclear	Recipients

HUNGARY	Act XCVIII of 2006 (provisions introduced in 2011)	National Institute of Pharmacy and Nutrition	Webpage	Yes	Yes	No	No	Donors; payment names, descriptions and amounts; date; recipient address	No	Unclear	No
THE NETHER-LANDS	Code of Conduct for Pharmaceutical Advertising (2012)	Foundation for the Code for Pharmaceutical Advertising	Webpage	Yes	Yes (recipient identifiers needed)	No	No	Donors; recipients; recipient categories; payment categories and amounts; year	Annually per payment type	Unclear	Recipients
GREECE	Law 4316/2014; Opinion No. 5/2016 and 2/2017 of the Data Protection Authority; circular No. 17770/2016 of the National Authority for Medicines	National Organisation for Medicines 1) payments to conference participants; 2) payments to conference organisers; drug company websites	PDFs – image-based	Yes	No	No	Yes	Payments to conference participants – donors; recipients; payment categories (types of conference expenditure) and amounts; year; Payments to conference organisers – donors; payment amounts; year	Payments to conference participants – annually per recipient; Payments to conference organisers – donors	Unclear	Donors
ESTONIA	Medicinal Products Act of 2005 (provisions introduced in 2013)	State Agency of Medicines	XLS	Yes	No	No	Yes	Donors; payment categories and amounts; payment location (country); year	Annually per donor	Unclear	No
TURKEY	Regulation on Promotional Activities of Medicinal Products for Human Use 2015	Ministry of Health (database not publicly available)	Unclear	Unclear	Unclear	Unclear	Unclear	Unclear	Unclear	Unclear	Unclear

Notes.

1 – Lighter colours indicate, respectively, higher, and darker colours – lower, data accessibility and quality. The databases are presented in the descending order of their overall data accessibility and quality, that is, the greater overall number of lighter cells a database has the higher its position within the table. Databases with equal numbers of lighter and darker cells are sorted alphabetically.

2 – This column provides the dates when public regulation of payment disclosure was first introduced. If public regulation of payment disclosure forms part of a larger piece of government regulation, it is specified – where appropriate – whether the regulation of payment disclosure was introduced as a change already existing government regulation. The dates reported here do not cover changes to or refinements of provisions focusing on payment disclosure.

3 – The disclosure requirements ordinarily cover both healthcare professionals and organisations. The exceptions are the Danish databases (only healthcare professionals) and the Turkish database (it is unclear whether disclosure requirements also cover healthcare organisations).

4 – Web links are accurate as of May 2021.

5 – The recipient addresses ordinarily refer to the location of the payment recipient. In the case of Hungarian, Latvian and Lithuanian databases we considered that the event addresses were equivalent to recipient addresses.

For peer review only - http://bmjopen.bmj.com/site/about/guidelines.xhtml

The databases established via public regulation or a combination of public and self-regulation had the pattern of accessibility similar to the industry-run databases. Of the thirteen databases none had customisable summary statistics, and only six were downloadable and fully searchable. Overall, Transparence Santé was the frontrunner.

The most frequent data quality shortcoming was unclear tax reporting, with only two databases providing relevant rules. However, over half of the databases had at least partial donor or recipient identifiers, which was the most frequent problem in the industry-run databases. Further, just five databases covered a spectrum of donor or recipient characteristics exceeding the minimum recommendations from the EFPIA Code . Transparence Santé again had the highest overall data quality.

In sum, Transparence Santé had combined data accessibility and quality exceeding that of Disclosure UK, the frontrunner industry database.

4. Discussion

Our policy review suggests that payment data disclosure does not automatically increase transparency of financial relationships between drug companies and the healthcare sector.[4 5] Consistently with research on disclosure of aspects of health policymaking by both public and private-sector actors, we find that achieving “practical” or “actionable” transparency is no less important than introducing transparency rules themselves.[67-69]

Although EFPIA calls payment data generated via self-regulation “open to public scrutiny”,[70] establishing the entanglement of any recipient, let alone a system-level picture, is impossible given the dispersal of disclosures on company websites in most European countries. Additionally, documents published as PDFs, sometimes in ways suggesting deliberate attempts to impede user engagement, fall below the Australian industry-endorsed regulations requiring firms to use an analysable format.[5] Therefore, self-regulation cannot address “the issues of perceived conflict of interest”,[71] as promised by EFPIA. More broadly, the evidence of some companies and trade groups meeting only the minimum requirements from the EFPIA Code, or fulfilling them in ways inconsistent with the Code’s spirit, reflects the limited success of self-regulation in modifying corporate behaviour in areas of public health policy such as reduction of sugar content in food[72] or managing viewers’ exposure to alcohol advertising.[73]

EFPIA is clearly aware of at least some of the problems in payment data accessibility. For example, in 2019, it listed “improv[ing] access” via “[c]reateing platforms with [a] searchable tool” as one of the “main topics” to be considered by EFPIA itself and its member trade groups.[23] However, little evidence exists of subsequent discussions on this issue except for a planned “feasibility study” of possible “options for improving the disclosure” to be considered from 2021 to 2023.[74] Further, EFPIA does not seem to have recognised or engaged with the issues of low payment data quality.

For peer review only - http://bmjopen.bmj.com/site/about/guidelines.xhtml

Against this background, eurosfordocs.eu radically enhances data accessibility in countries without centralised industry databases, also enabling comparative investigations of country payment patterns,[28] which is important given the accelerating EU-wide health initiatives.[75] Although the customisable opportunities for data exploration are new to the public, data analytics firms have offered them as a consultancy service to drug companies.[76 40] Consequently, eurosfordocs.eu may contribute to changing what may be the de-facto status of payment data as a commodity used to monitor internal compliance with disclosure requirements and potentially inform marketing strategies targeting healthcare professionals.[77]

In countries with self-regulation, the challenges in data accessibility and quality are exacerbated by non-disclosed payments. EFPIA admits the problem of "[c]onsent issues in general but also by country and by speciality",[23] while evidence also exists of varying consent rates between companies.[28] In addition, some companies may not disclose all their payments, as suggested by instances of underreporting of payments to patient organisations, with their disclosure also regulated by the EFPIA Code but with distinct policies.[78 79] Further, self-regulation only covers companies and trade groups that have ratified the EFPIA Code or its transposition into country-level codes. Therefore, disclosure requirement may not extend to companies focusing on generic or over-the-counter medicines and even major manufacturers of branded prescription medicines (e.g. Vertex does not follow the ABPI Code). However, some non-member companies may choose to follow the trade group codes. For example, the list of Disclosure UK participants exceeds ABPI membership.[79] Further, some companies may belong to other trade groups (e.g. generic or small biotech trade groups), which, in some countries, require their members to abide by the national Codes (e.g. Sweden, Denmark). Problems with underreported payments may be particularly prominent in countries with parallel self- and public regulation due to possible confusion relating to where payments should be reported. For example, some healthcare organisations in England underreported some of the payments they had received given their implicit or explicit expectations that the payments would be disclosed via self-regulation.[30 31]

Although data reported in the US Open Payments database has attracted some criticism,[25 80] its accessibility and quality are vastly superior to European data disclosed using self-regulation but also public regulation. While Transparence Santé indicates that public regulation can generate payment data outpacing industry-run databases, it often shares major shortcomings with self-regulation, including the lack of recipient identifiers or payment itemisation.[24 26] Moreover, in some databases the spectrum of disclosed characteristics is even narrower than the minimum which EFPIA recommends for the industry. Nevertheless, public regulation eliminates optionality characterising the EFPIA Code, regarding, for example, centralised databases. The legally binding nature of public regulation should also involve high levels of compliance. However, instances of inaccurate or incomplete reporting by some companies are possible.[25]

Inconsistencies in the approaches to public regulation between European counties are highlighted by EFPIA and used as a key argument in favour of self-regulation, which, in EFPIA's words, represents a "global and consistent approach for companies across Europe and common understanding for the public".[23] France is one country in which problems in

For peer review only - http://bmjopen.bmj.com/site/about/guidelines.xhtml

data accessibility and "ergonomics" have been recognised by the Ministry of Health in 2018.[81] Following this, a new version of Transparence Santé is due to be launched in late 2021, and is expected to adopt approaches to data presentation, including visualisations, similar to those developed earlier for eurosfordocs.fr. We are not aware of similar discussions in other countries with public regulation or combining self- and public regulation.

Therefore, one key area of further study would involve using qualitative methods to identify and trace relationships between the likely causes of limited corrective action seeking to address the shortcomings of the current reporting systems in European countries. Of particular importance would be examining the incentive structures and motivations of public authorities, industry trade groups and companies, healthcare professional associations and patient organisations at the national and EU levels.

4.1. Limitations

This study has several limitations. Our measures of data accessibility could be expanded. For example, some databases are difficult to find, including web links to the Greek and Latvian databases published within news releases, without permanent online location. Similarly, although Transparence Santé can be downloaded, the size of the dataset prevents it from being opened using the standard Excel package. Data quality could be scrutinised further by considering the types of disclosed donors, payments and recipients.[4] Further, qualitative insights from data users would be essential for ranking the outcome measures and attributing weights to their values, such as degrees of user-friendliness.

Our focus on the database level might obscure cross-company differences. For example, the widely ranging consent rates achieved by companies from healthcare professionals suggests that similar differences can occur in data quality and accessibility.[21 28] Further, we did not calculate company-level aspects of data accessibility (e.g. the share of image-based PDFs) and quality (e.g. the share of duplicate entries, consistency in using donor or recipient categories and identifiers, missing data, and mistakes, such as negative values). Undertaking these calculations would have necessitated extensive forensic work.[24] However, these problems are likely to be widespread and serious, affecting even Transparence Santé, the database we ranked the highest based on its quality .[21 24 26]

4.2. Conclusions and policy recommendations

We formulate suggestions for enhancing public engagement with disclosed payment data. (Table 5), which are also relevant for non-European countries, such as Japan, experiencing problems similar to those identified in this study.[82]

For peer review only - http://bmjopen.bmj.com/site/about/guidelines.xhtml

Table 5 How can public authorities and the pharmaceutical industry improve the transparency of payment data?

	**Recommendations for improving accessibility of payment data**
1	Create national-level databases searchable for companies, recipients, and payment categories.
2	Make the databases in the CSV or XLS format for further analysis, while ensuring that the released data can be split using different variables, for example, by year or recipient type to make it manageable for users.
3	Enable users to explore the data by allowing them to generate data summaries placing payments made or received in a broader context (e.g. payments made by other companies or received by the same or other recipient categories, such as medical specialty).
	**Recommendations for improving quality of payment data**
4	Publish unique identifiers for payment recipients shared by all companies and used consistently over time.
5	Introduce clear rules on the levels of aggregation for identifying recipients (e.g. clinic, ward, or hospital) to enhance the consistency of reporting.
6	Introduce categories of recipients to enable mapping the distribution of payments in the healthcare system. The categories relating to healthcare professionals could include a standardised list of medical specialties. The categories covering healthcare organisations could reflect their functions in the healthcare system as providers, commissioners, or professional organisations.
7	State clearly whether reported payments should include VAT or other taxes so that payment values from different companies can be compared reliably.
8	Publish each payment individually instead of aggregating them annually per recipient.
9	Publish payment descriptions so that the public can understand the activities they fund as well as their context. This requirement would follow the self-regulatory rules existing in relation to the disclosure of payments to patient organisations.
10	Enforce and publish detail of data quality checks: eliminate missing values, payments with the value of zero, and ensure that each recipient has a unique name and is reported at the same level of aggregation by all companies. Other data quality checks should involve cross-checking recipient name and address information to ensure consistency and avoid duplicate reporting.

For peer review only - http://bmjopen.bmj.com/site/about/guidelines.xhtml

Payment data accessibility can be enhanced with only minor revisions of the existing regulatory approaches, with the top priority being centralised databases offering possibilities for payment exploration and contextualisation.

Improving payment data quality would require new comprehensive public regulation, preferably at the European level.[4 28] Following Open Payments, payments should be reported together with information on related products to allow exploring company marketing strategies.[24 83] Another vital piece of information to include might be the numbers of clinical trials associated with payments, as exemplified by the database run by the Slovak National Health Information Center. Further, granular disclosure is vital for capturing payments of different sizes, with some US studies suggesting that even small payments impact prescribing behaviour,[84 85] while others indicating a more complex dose-effect relationship.[11 29 86 87] Data interpretation can be enhanced by descriptions of funded activities (e.g. specific conferences or projects), consistent with the EFPIA Code's requirements regarding payments to patient organisations.[79 88] Recipient characteristics should be also expanded, reflecting how the public engages with the healthcare system.[24] Lastly, Open Payments highlights that recipient identifiers are necessary for reliable analysis and connecting payment data to datasets with details of prescription and procurement.[11 29 84 85 87] Data integration and management requires strong compliance mechanisms, including penalties for providing data of inadequate quality.[25]

Additionally, in European countries with self-regulation, eliminating possibilities for refusing disclosure by recipients is necessary to reduce high levels of missing data.[28] The decision by the Spanish Data Protection Authority is illustrative here, exempting payment data from the provisions of the European data protection legislation (GDPR).[28]

Finally, transparency alone cannot address FCOIs. Even the increased transparency brought in by Open Payments does not seem have decreased physicians' acceptance of FCOIs or increased patient concerns about their possible effects on the care they receive.[89] Paradoxically, transparency may normalise FCOIs or increase their impact via moral licensing.[89] Therefore, transparency should be accompanied by policy measures seeking to reduce or eliminate certain FCOIs. Key European examples include banning some financial relationships,[90] including payments to healthcare professionals for conference participation in Sweden[28] or prohibiting sponsored meals over €60 in France.[91]

For peer review only - http://bmjopen.bmj.com/site/about/guidelines.xhtml

Contributors

PO is Senior Lecturer at the Department of Social and Policy Sciences, University of Bath. PO conceived and wrote the paper, collected and analysed the data. LM is a data scientist and the President of the Euros for Docs Association. LM created the eurosfordocs.eu database, analysed the data and contributed to writing. PAJ formerly presided the Euros for Docs Association. PAJ collaborated with LM on creating eurosfordocs.eu. PAJ conceived the paper and contributed to writing. SM is Associate Professor at the Department of Sociology, Lund University. SM conceptualized the paper and contributed to writing.

Acknowledgements

We would like to express gratitude to all pharmaceutical industry trade groups and public authorities which have responded to our request for information and were willing to explain the nature of local regulatory solutions. We thank, in particular, the ABPI's Disclosure UK Team for their continued interest in our research as well as professionalism and openness in sharing the fine detail of their important work. We extend our thanks to colleagues who took the time to read drafts of this paper or offer linguistic assistance: Marcell Csanadi, Eniko Csanadi, Alice Fabbri, Kevin Jean, Fatma Korkmaz, Olga Loblova, Savvas Morris, Emily Rickard, Eszter Saghy, Christos Vasilakis, and Weronika Ozieranska.

We are also thankful to the BMJ Open Editors and three Reviewers for their helpful and constructive comments.

Funding

This work was supported by The Swedish Research Council (VR), grant number 2020-01822 ("Following the money: cross-national study of pharmaceutical industry payments to medical associations and patient organisations").

Details of the role of study sponsors

The funding body played no part in the design or conduct of this study

Statement of independence of researchers from funders

All researchers working on this study are independent from the funding body.

Competing interests

We have read and understood the BMJ Group policy on declaration of interests and declare the following interests:

For peer review only

PO's PhD student was supported by a grant from Sigma Pharmaceuticals, a UK pharmacy wholesaler and distributor (not a pharmaceutical company). The PhD work funded by Sigma Pharmaceuticals is unrelated to the subject of this paper.
LM and PAJ are members of Euros for Docs, a non-profit organization registered in France that seeks to promote transparency of drug company funding in the healthcare sector by making payment data accessible and complete across Europe.
PAJ is employed by Haute Autorité de Santé, the French independent health technology assessment organisation.
SM's partner is employed by PRA Health Sciences, a global Contract Research Organization whose costumers include many pharmaceutical companies.

Patient consent

None required.

Ethical approval

No ethical approval was needed. The ethical implications of this study article were approved via a peer ethics review process at the Department of Social and Policy Sciences, University of Bath in April 2016. This study did not require a full ethical approval as it relied on publicly available data aggregated at the organisational or country level.

Data sharing

We have included all relevant data as supplementary information forming part of this submission.

Exclusive license

The Corresponding Author has the right to grant on behalf of all authors and does grant on behalf of all authors, a worldwide licence to the Publishers and its licensees in perpetuity, in all forms, formats and media (whether known now or created in the future), to i) publish, reproduce, distribute, display and store the Contribution, ii) translate the Contribution into other languages, create adaptations, reprints, include within collections and create summaries, extracts and/or, abstracts of the Contribution, iii) create any other derivative work(s) based on the Contribution, iv) to exploit all subsidiary rights in the Contribution, v) the inclusion of electronic links from the Contribution to third party material where-ever it may be located; and, vi) licence any third party to do any or all of the above."

Transparency declaration

The guarantor (Piotr Ozieranski) affirms that this manuscript is an honest, accurate, and transparent account of the study being reported; that no important aspects of the study have been omitted; and that any discrepancies from the study as planned (and, if relevant, registered) have been explained.

For peer review only

References

1. Lo B, Field MJ. Conflict of Interest in Medical Research, Education, and Practice: National Academic Press; 2009 [Available from: https://www.nap.edu/catalog/12598/conflict-of-interest-in-medical-research-education-and-practice.
2. Lundh A, Lexchin J, Mintzes B, et al. Industry sponsorship and research outcome. *Cochrane Database of Systematic Reviews* 2017(2) doi: 10.1002/14651858.MR000033.pub3
3. Torgerson T, Wayant C, Cosgrove L, et al. Ten years later: a review of the US 2009 institute of medicine report on conflicts of interest and solutions for further reform. *BMJ Evidence-Based Medicine* 2020:bmjebm-2020-111503. doi: 10.1136/bmjebm-2020-111503
4. Fabbri A, Santos Al, Mezinska S, et al. Sunshine Policies and Murky Shadows in Europe: Disclosure of Pharmaceutical Industry Payments to Health Professionals in Nine European Countries. *International Journal of Health Policy and Management* 2018;7(6):504-09. doi: 10.15171/ijhpm.2018.20
5. Grundy Q, Habibi R, Shnier A, et al. Decoding disclosure: Comparing conflict of interest policy among the United States, France, and Australia. *Health Policy* 2018;122(5):509-18. doi: https://doi.org/10.1016/j.healthpol.2018.03.015
6. Mental Health Europe. Sunshine and transparency laws and regulations and codes across Europe 2017 [updated April 2017. Available from: https://mhe-sme.org/wp-content/uploads/2017/09/Mapping-of-Sunshine-Laws-in-Europe.pdf.
7. Santos A. The sun shines on Europe: transparency of financial relationships in the healthcare sector 2017 [10.
8. McDermott Will & Emery. Snapshot of Sunshine rules in EU countries for the pharmaceutical industry 2014 [updated June 2014. Available from: https://mcdermott-will-emery-2793.docs.contently.com/v/special-report-snapshot-of-sunshine-rules-in-eu-countries-for-the-pharmaceutical-industry1.
9. Moynihan R, Albarqouni L, Nangla C, et al. Financial ties between leaders of influential US professional medical associations and industry: Cross sectional study. *The BMJ* 2020;369 doi: 10.1136/bmj.m1505
10. Garstka ME, Monlezun D, DuCoin C, et al. The Sunshine Act and Surgeons: A Nation-Wide Analysis of Industry Payments to Physicians. *Journal of Surgical Research* 2019;233:41-49. doi: https://doi.org/10.1016/j.jss.2018.07.004
11. Yeh JS, Franklin JM, Avorn J, et al. Association of Industry Payments to Physicians With the Prescribing of Brand-name Statins in Massachusetts. *JAMA Internal Medicine* 2016;176(6):763-68. doi: 10.1001/jamainternmed.2016.1709
12. Mejia J, Mejia A, Pestilli F. Open data on industry payments to healthcare providers reveal potential hidden costs to the public. *Nature Communications* 2019;10(1):4314. doi: 10.1038/s41467-019-12317-z
13. Mitchell AP, Trivedi NU, Gennarelli RL, et al. Are financial payments from the pharmaceutical industry associated with physician prescribing? A systematic review. *Annals of Internal Medicine* 2020;Epub ahead of print doi: doi:10.7326/M20-5665 [published Online First: 24 Nov 2020]

For peer review only

14. Wayant C, Walters C, Zaaza Z, et al. Evaluation of Financial Conflicts of Interest Among Physician-Authors of American College of Rheumatology Clinical Practice Guidelines. *Arthritis & rheumatology* 2020;72(9):1427-34. doi: 10.1002/art.41224
15. Journal of Oral and Maxillofacial Surgery. *Journal of Oral and Maxillofacial Surgery*
16. El-Rayess H, Khamis AM, Haddad S, et al. Assessing concordance of financial conflicts of interest disclosures with payments' databases: a systematic survey of the health literature. *Journal of clinical epidemiology* 2020;127:19-28. doi: 10.1016/j.jclinepi.2020.06.040
17. Ornstein C, Weber T, Jones RG. We Found Over 700 Doctors Who Were Paid More Than a Million Dollars by Drug and Medical Device Companies, 2019.
18. Department of Justice - Office of Public Affairs. Medtronic to Pay Over $9.2 Million To Settle Allegations of Improper Payments to South Dakota Neurosurgeon, 2020.
19. EFPIA. EFPIA Code of practice (2019) 2019 [Available from: https://www.efpia.eu/relationships-code/the-efpia-code/.
20. Mulinari S, Davis C, Ozieranski P. Failure of Responsive Regulation? Pharmaceutical Marketing, Corporate Impression Management and Off-Label Promotion of Enzalutamide in Europe. *Journal of White Collar and Corporate Crime* 2020:2631309X097047. doi: 10.1177/2631309x20970477
21. Mulinari S, Ozieranski P. Disclosure of payments by pharmaceutical companies to healthcare professionals in the UK: analysis of the Association of the British Pharmaceutical Industry's Disclosure UK database, 2015 and 2016 cohorts. *BMJ Open* 2018;8(10):e023094. doi: 10.1136/bmjopen-2018-023094
22. Adlington K, Godlee F. Disclosure UK: transparency should no longer be an optional extra. *BMJ* 2016;354:i3730. doi: 10.1136/bmj.i3730
23. EFPIA. Codes Committee Activities Report (2018) 2019 [Available from: https://www.efpia.eu/media/554642/efpia-code-report-2018.pdf.
24. Ozieranski P, Csanadi M, Rickard E, et al. Analysis of Pharmaceutical Industry Payments to UK Health Care Organizations in 2015. *JAMA Network Open* 2019;2(6):e196253-e53. doi: 10.1001/jamanetworkopen.2019.6253
25. Adashi EY, Cohen IG. Enforcement of the Physician Payments Sunshine Act: Trust and Verify. *JAMA* 2021 doi: 10.1001/jama.2021.13156
26. Stoll M, Hubenschmid L, Koch C, et al. Voluntary disclosures of payments from pharmaceutical companies to healthcare professionals in Germany: a descriptive study of disclosures in 2015 and 2016. *BMJ Open* 2020;10(9):e037395. doi: 10.1136/bmjopen-2020-037395
27. Moriarty F, Larkin J, Fahey T. Payments reported by the pharmaceutical industry in Ireland from 2015 to 2019: an observational study. *Health Policy* 2021 doi: https://doi.org/10.1016/j.healthpol.2021.07.016
28. Mulinari S, Martinon L, Jachiet P-A, et al. Pharmaceutical industry self-regulation and non-transparency: country and company level analysis of payments to healthcare professionals in seven European countries. *Health Policy* 2021 doi: https://doi.org/10.1016/j.healthpol.2021.04.015
29. Goupil B, Balusson F, Naudet F, et al. Association between gifts from pharmaceutical companies to French general practitioners and their drug prescribing patterns in 2016: retrospective study using the French Transparency in Healthcare and National Health Data System databases. *BMJ* 2019;367:l6015. doi: 10.1136/bmj.l6015

For peer review only

30. Moberly T. NHS joint working with industry is out of public sight. *BMJ* 2019;364:l1353. doi: 10.1136/bmj.l1353
31. Moberly T. CCGs fail to declare pharma funding. *BMJ* 2018;360:j5911. doi: 10.1136/bmj.j5911
32. U.S. Attorney's Office - District of New Jersey. Novartis AG and Subsidiaries to Pay $345 Million to Resolve Foreign Corrupt Practices Act Cases 2020 [updated 25th June 2020. Available from: https://www.justice.gov/usao-nj/pr/novartis-ag-and-subsidiaries-pay-345-million-resolve-foreign-corrupt-practices-act-cases.
33. Securities and Exchange Commission. SEC Charges Eli Lilly and Company with FCPA Violations 2012 [updated 20th December 2012. Available from: https://www.sec.gov/news/press-release/2012-2012-273htm.
34. Eurosfordocs.eu. Metabase 2021 [Available from: https://eurosfordocs.eu/metabase/.
35. Anon. Hôpitaux : les CHU ont reçu 170 millions d'euros de laboratoires pharmaceutiques en 2018 2020 [updated 10th January 2020. Available from: https://www.lemonde.fr/sante/article/2020/01/10/hopitaux-les-chu-ont-recu-170-millions-d-euros-de-laboratoires-pharmaceutiques-en-2018_6025479_1651302.html
36. Anon. Procès du Mediator : « Le terreau des conflits d'intérêts à l'hôpital reste fertile » 2020 [updated 4th February 2020. Available from: https://www.lemonde.fr/idees/article/2020/02/04/le-terreau-des-conflits-d-interets-a-l-hopital-reste-fertile_6028302_3232.html.
37. Condomines A. Qu'est-ce que la plateforme EurosForDocs, qui recense les liens d'intérêts avec les industriels pharmaceutiques? 2021 [updated 4th January 2021. Available from: https://www.liberation.fr/checknews/2021/01/04/qu-est-ce-que-la-plateforme-eurosfordocs-qui-recense-les-liens-d-interets-avec-les-industriels-pharm_1809498.
38. Eaton L. Declarations of pharma funding to doctors are hit by covid-19 crisis. *BMJ* 2020;370:m2689. doi: 10.1136/bmj.m2689
39. Zetterqvist AV, Merlo J, Mulinari S. Complaints, Complainants, and Rulings Regarding Drug Promotion in the United Kingdom and Sweden 2004–2012: A Quantitative and Qualitative Study of Pharmaceutical Industry Self-Regulation. *PLOS Medicine* 2015;12(2):e1001785. doi: 10.1371/journal.pmed.1001785
40. MediSpend. Global Compliance Platform 2021 [Available from: https://www.medispend.com/solutions/global-compliance/ accessed 9th January 2021 2021.
41. Agencia Española de Protección de Datos. INFORME 1438818/2016 2016 [
42. EFPIA. EFPIA HCP/HCO DISCLOSURE CODE. EFPIA CODE ON DISCLOSURE OF TRANSFERS OF VALUE FROM PHARMACEUTICAL COMPANIES TO HEALTHCARE PROFESSIONALS AND HEALTHCARE ORGANISATIONS 2014 [Available from: https://www.efpia.eu/media/25837/efpia-disclosure-code.pdf.
43. Shire. EFPIA Disclosure Code 2018 Disclosures Shire Pharmaceuticals 2018 [Available from: https://www.takeda.com/4ab3b3/siteassets/system/who-we-are/values-and-corporate-governance/transparency-disclosures/efpia-disclosure-code-reports/2018/methodology_serbia_english.pdf.
44. APL. Code de déontologie 2020 [Available from: https://www.apl-pharma.lu/docs/33850f/text/2021-code-de-deontologie-apl.pdf.
45. Danish Medicines Agency. Healthcare professionals having a relationship with a company 2020 [updated 23 November 2020. Available from:

For peer review only

https://laegemiddelstyrelsen.dk/en/licensing/relationships/ accessed 17th August 2021.

46. Ethical Committe for the Pharmaceutical Industry. Ethical rules for the pharmaceutical industry’s donations and grants (Donation Code) N/A [Available from: https://www.enli.dk/media/49893/ethical-rules-on-donations-and-grants-version-20.pdf.
47. Amgen. Methodological Note. EFPIA & Local Industry Association Disclosure Codes 2020 [Available from: https://search.disclosureuk.org.uk/Public/GetMethodNoteFileForDownload?year=2019&pharmaCompanyId=29 accessed 25th February 2020.
48. GSK. Engaging with healthcare professionals - Turkey 2020 [Available from: https://www.gsk.com/en-gb/responsibility/operating-responsibly/engaging-with-healthcare-professionals/europe/turkey/ accessed 18th January 2021.
49. betransparent.be. About us 2020 [Available from: https://www.betransparent.be/en/about-us/ accessed 18th January 2020.
50. Sunshine act 2016 [Available from: https://www.betransparent.be/wp-content/uploads/2017/01/Moniteur-Belge-2016-12-27-Belgisch-Staatsblad.pdf.
51. betransparent.be. Transparency in the interest of the patient 2020 [Available from: https://www.betransparent.be/en/about-the-transparency-register/ accessed 18th January 2020.
52. GSK. Engaging with healthcare professionals - Belgium 2020 [Available from: https://www.gsk.com/en-gb/responsibility/operating-responsibly/engaging-with-healthcare-professionals/europe/belgium/ accessed 18th January 2021.
53. Medicines Act 395/1987 1987 [Available from: https://www.fimea.fi/documents/160140/765540/18580_Laakelaki_englanniksi_paivitetty_5_2011.pdf.
54. Stichting Transparantieregister Zorg. Over het Transparantieregister 2020 [Available from: https://www.transparantieregister.nl/over-het-transparantieregister accessed 18th January 2020.
55. Stichting Transparantieregister Zorg. Hoe werken we? 2020 [Available from: https://www.transparantieregister.nl/over-het-transparantieregister/hoe-werken-we accessed 28th February 2021.
56. Stichting Transparantieregister Zorg. Veelgestelde vragen 2020 [Available from: https://www.transparantieregister.nl/veelgestelde-vragen.
57. Eurosfordocs.eu. Tech documentation 2021 [Available from: https://eurosfordocs.eu/on-the-tech-side/tech-documentation/ accessed 9th January 2021.
58. FSA. Transparenzkodex 2020 [updated 30th June 2020. Available from: https://www.fsa-pharma.de/de/mitteilungen/presse/archiv/transparenzveroffentlichungen-2019/.
59. FARMAINDUSTRIA. Las compañías farmacéuticas dedicaron el año pasado 483 millones a actividades de investigación y formación científica con organizaciones y profesionales sanitarios 2020 [updated 26th June 2020. Available from: https://www.farmaindustria.es/web/prensa/notas-de-prensa/2020/06/26/las-companias-farmaceuticas-dedicaron-el-ano-pasado-483-millones-a-actividades-de-investigacion-y-formacion-cientifica-con-organizaciones-y-profesionales-sanitarios/.
60. ScienceIndustries. Disclosure obligation since 2016 2020 [updated 22nd September 2020. Available from:

For peer review only

https://www.scienceindustries.ch/en/article/13958/disclosure-obligation-since-2016.
61. EFPIA. Report on Ethics & Compliance Activities (2019) 2020 [Available from: https://www.efpia.eu/media/554639/efpia-code-report-2019.pdf.
62. IPHA. Email communication, 2020.
63. ABPI. Disclosure UK 2020 [Available from: https://www.abpi.org.uk/our-ethics/disclosure-uk/.
64. CurrencyConverter. CurrencyConverter 0.14.4 2020 [Available from: https://pypi.org/project/CurrencyConverter/ accessed 19th January 2021.
65. ABPI. Code of practice for the pharmaceutical industry 2019 [Available from: https://www.abpi.org.uk/media/6655/abpi-code-of-practice-2019.pdf.
66. ABPI. Email communication, 2021.
67. Ozierański P, Löblová O, Nicholls N, et al. Transparency in practice: Evidence from 'verification analyses' issued by the Polish Agency for Health Technology Assessment in 2012–2015. *Health Economics, Policy and Law* 2019;14(2):182-204. doi: 10.1017/S1744133117000342 [published Online First: 2018/01/08]
68. Bullement A, Taylor M, McMordie ST, et al. NICE, in Confidence: An Assessment of Redaction to Obscure Confidential Information in Single Technology Appraisals by the National Institute for Health and Care Excellence. *PharmacoEconomics* 2019;37(11):1383-90. doi: 10.1007/s40273-019-00818-0
69. Csanádi M, Ozierański P, Löblová O, et al. Shedding light on the HTA consultancy market: Insights from Poland. *Health Policy* 2019;123(12):1237-43. doi: https://doi.org/10.1016/j.healthpol.2019.08.008
70. EFPIA. Annual Review of 2011 and Outlook for 2012 2011 [Available from: https://www.efpia.eu/media/25261/annual-review-of-2011-and-outlook-for-2012.pdf.
71. EFPIA. Annual Review of 2011 and Outlook for 2012. 2011
72. Seferidi P, Millett C, Laverty AA. Industry self-regulation fails to deliver healthier diets, again. *BMJ* 2021;372:m4762. doi: 10.1136/bmj.m4762
73. Noel JK, Babor TF, Robaina K. Industry self-regulation of alcohol marketing: a systematic review of content and exposure research. *Addiction* 2017;112(S1):28-50. doi: https://doi.org/10.1111/add.13410
74. EFPIA. EFPIA Report on Ethics & Compliance Activities (2020) 2021 [Available from: https://www.efpia.eu/media/602865/efpia-code-report-2020-20210629.pdf.
75. European Commission. COMMUNICATION FROM THE COMMISSION TO THE EUROPEAN PARLIAMENT, THE COUNCIL, THE EUROPEAN ECONOMIC AND SOCIAL COMMITTEE AND THE COMMITTEE OF THE REGIONS Pharmaceutical Strategy for Europe 2020 [Available from: https://eur-lex.europa.eu/legal-content/EN/TXT/?uri=CELEX:52020DC0761.
76. IQVIA. Get to better business processes with embedded compliance 2021 [Available from: https://www.iqvia.com/solutions/integrated-global-compliance/commercial-compliance/ accessed 9th January 2021 2021.
77. Anand R, Collier D, Jiang Y, et al. Leveraging CMS Open Payments Data to Identify Channel Preferences and Gather Competitive Intelligence, Thereby Improving HCP Targeting ND [Available from: https://www.pmsa.org/jpmsa-vol05-article06.

For peer review only

78. Ozieranski P, Csanadi M, Rickard E, et al. Under-reported relationship: a comparative study of pharmaceutical industry and patient organisation payment disclosures in the UK (2012–2016). *BMJ Open* 2020;in press
79. Rickard E, Ozieranski P, Mulinari S. Evaluating the transparency of pharmaceutical company disclosure of payments to patient organisations in the UK. *Health Policy* 2019
80. Department of Health and Human Services - Office of Inspector General. Open Payments Data: Review of Accuracy, Precision, and Consistency in Reporting 2018 [Available from: https://oig.hhs.gov/oei/reports/oei-03-15-00220.pdf.
81. Ministère des Solidarités et de la Santé. Discours d'Agnès Buzyn: remise du rapport sur l'amélioration de l'information des usagers et des professionnels de santé sur le médicament 2018 [updated 3rd September 2018. Available from: https://solidarites-sante.gouv.fr/actualites/presse/discours/article/discours-d-agnes-buzyn-remise-du-rapport-sur-l-amelioration-de-l-information accessed 29th October 2021.
82. Ozaki A, Saito H, Senoo Y, et al. Overview and transparency of non-research payments to healthcare organizations and healthcare professionals from pharmaceutical companies in Japan: Analysis of payment data in 2016. *Health Policy* 2020;124(7):727-35. doi: https://doi.org/10.1016/j.healthpol.2020.03.011
83. Greenway T, Ross JS. US drug marketing: how does promotion correspond with health value? *BMJ* 2017;357:j1855. doi: 10.1136/bmj.j1855
84. DeJong C, Aguilar T, Tseng C-W, et al. Pharmaceutical Industry–Sponsored Meals and Physician Prescribing Patterns for Medicare Beneficiaries. *JAMA Internal Medicine* 2016;176(8):1114-22. doi: 10.1001/jamainternmed.2016.2765
85. Fleischman W, Agrawal S, King M, et al. Association between payments from manufacturers of pharmaceuticals to physicians and regional prescribing: cross sectional ecological study. *BMJ* 2016;354:i4189. doi: 10.1136/bmj.i4189
86. Mitchell AP, Trivedi NU, Gennarelli RL, et al. Are Financial Payments From the Pharmaceutical Industry Associated With Physician Prescribing? *Annals of Internal Medicine* 2020;174(3):353-61. doi: 10.7326/M20-5665
87. Nguyen TD, Bradford WD, Simon KI. Pharmaceutical payments to physicians may increase prescribing for opioids. *Addiction* 2019;114(6):1051-59. doi: https://doi.org/10.1111/add.14509
88. Ozieranski P, Rickard E, Mulinari, Shai. Exposing drug industry funding of UK patient organisations. *BMJ* 2019;365:l1806. doi: 10.1136/bmj.l1806
89. Lexchin J, Fugh-Berman A. A Ray of Sunshine: Transparency in Physician-Industry Relationships Is Not Enough. *Journal of General Internal Medicine* 2021 doi: 10.1007/s11606-021-06657-0
90. Parker L, Karanges EA, Bero L. Changes in the type and amount of spending disclosed by Australian pharmaceutical companies: an observational study. *BMJ Open* 2019;9(2):e024928. doi: 10.1136/bmjopen-2018-024928
91. Ganem-Chabenet D. LOI ANTI-CADEAU : LES NOUVEAUTÉS DU DÉCRET DU 15 JUIN 2020 SUR LES AVANTAGES OFFERTS AUX PROFESSIONNELS DE SANTÉ 2020 [Available from: https://emservice.fr/ems/index.php/2020/09/10/breve_dgc_septembre_2020/ accessed 19th March 2021.

For peer review only

Online Supplement 1. Stakeholder survey

Stakeholder survey, Part 1: Questions to national pharmaceutical industry trade groups

Dear Sir or Madam

I would be grateful if you could answer a few questions listed below regarding the way in which your organisation and its Members disclose transfers of value to healthcare professionals and organisations as well as patient organisations. Your answers would be very helpful in informing scholarly publications on which I am currently working that are seeking to present a full and accurate picture of transfers of value in Europe. Please let me know should you have any questions about this research.

[if relevant] In particular, your answers would help me expand on the information your Association provided in a recent EFPIA report and on its webpages, including here. Some of the matters covered in my questions have been touched upon in the sources of data referred to above, although incompletely. I would therefore be grateful if you could answer my questions so that I report correct and accurate information in any research outputs.

Thank you very much in anticipation for your valuable time in answering my questions.

I look forward to hearing from you.

Sincerely yours

Piotr Ozieranski

For peer review only

I would be grateful if you could answer the following questions regarding your Association

1. What is the number of member companies in your Association?
2. How many companies which are not members of your Association follow its Code of Practice?
3. Please could you send me the most recent version of your Association's code of practice (if available, I would appreciate an English version of the document)?

Please could you answer the following questions regarding the platform and mechanisms of disclosure of transfers of value.

4. What is the platform for disclosing transfers of value to healthcare professionals and organisations (e.g. individual pharmaceutical company websites, a single database of transfers of value from all companies)?
5. If transfers of value are disclosed on individual pharmaceutical company websites, does your Association have a website providing links to these websites? If so, please could you provide me with a web link?
6. If transfers of value are disclosed on individual pharmaceutical company websites, has your Association considered creating a single database of transfers of value from all companies?
7. Do healthcare professionals in your country have to consent to the transfers of value being publicly disclosed; or is the disclosure of transfers of value to healthcare professionals mandatory (i.e. healthcare professionals are not asked to consent)? If the disclosure of transfers of value they received is mandatory, please could you state the regulation which makes them mandatory?
8. If healthcare professionals in your country have to consent to the transfers of value being publicly disclosed, does your Association have any expectations regarding the minimum HCP consent rate that should be achieved by companies signing up to its Code of Practice? If so, please could you specify this consent rate? Further, does your Association request companies achieving lower-than-expected healthcare professional consent rates to explain why this might be the case and identify possible ways of improving the consent rates?
9. If healthcare professionals in your country have to consent to the transfers of value being publicly disclosed, has your association considered implementing a simple summary disclosure rate statistic that is easily comparable between companies, such as the percent of the amount of transfers of value disclosed in the aggregate and/or the percent of healthcare professionals disclosed in the aggregate?
10. If healthcare professionals in your country have to consent to the transfers of value being publicly disclosed, does your association have any specific guidelines on how HCP disclosure rates could be improved by its Member Companies? If so, I would be grateful if you could share these guidelines with me?
11. If healthcare organisations have to consent to the transfers of value being publicly disclosed could you provide examples of reasons that would necessitate placing them in the aggregate disclosure?
12. Please could you state the threshold for the acceptable value of meals and drinks that was set by your association?
13. If some transfers of value need to be disclosed in a database run by a public institution, are there any transfers of value (e.g. research and development) that are still disclosed by pharmaceutical companies themselves. If so, I would be grateful if you could specify what these transfers of value are and why they are disclosed by pharmaceutical companies.
14. As far as your Association is aware, do companies disclosing their transfers of value use "unique country identifiers" recommended in the EFPIA Code of Practice, and not only recipient names or locations, to distinguish payment recipients? If so, are these identifiers

For peer review only

shared among different companies so that they can identify the same recipient using the same identifier?

15. Did members of your Association (or other companies following your Association's code of practice) disclose transfers of value to healthcare professionals and organisations in 2020 (i.e. covering payments made in the 2019 calendar year)?

If members of your Association (or other companies following your organisation's code of practice) disclosed transfers of value to healthcare professionals and organisations in 2020 (i.e. covering the 2019 calendar year) please could you answer the following questions.

16. How many pharmaceutical companies in total disclosed transfers of value to healthcare professionals and organisations made in 2019? How many of those companies were members of your Association?
17. Is the number of companies which did not disclose any transfers of value made in 2019 known? If so, please could you state this number? How many of those companies were members of your Association?
18. What was the overall share of healthcare professionals consenting to their transfers of value being disclosed in 2019?
19. What was the overall share of healthcare organisations consenting to their transfers of value being disclosed in 2019?
20. What was the total value of all non-research transfers of value to all healthcare professionals and organisations made in 2019?
21. What was the total value of all non-research transfers of value to all healthcare organisations made in 2019?
22. What was the value of all research-related (i.e. R&D) transfers of value to healthcare professionals or organisations made in 2019?
23. What share of the overall value of transfers of value made to healthcare professionals disclosed at the individual level in your country in 2019?
24. What share of the overall value of transfers of value made to healthcare organisations was disclosed at the individual level in your country in 2019?
25. What was the value of grants and donations made to healthcare organisations in 2019?
26. What was the value of fees for services and consultancy made to healthcare organisations in 2019?
27. What was the value of contributions to costs of events made to healthcare organisations in 2019?
28. What was the value of fees for services and consultancy made to healthcare professionals in 2019?
29. What was the value of contributions to costs of events made to healthcare professionals in 2019?

Finally, I would be grateful if you could answer the following questions regarding the public disclosure of transfers of value to patient organisations.

30. Please could you send me the most recent version of your Association's code of practice as it relates to working with patient organisations (if available, I would appreciate an English version of the document)?
31. Are there any companies that are not members of your Association which disclose transfers of value to patient organisations in line with the EFPIA Code of practice?
32. How many companies disclosed transfers of value to patient organisations made in 2019? How many of those companies were members of your Association?
33. What was the total value of all transfers of value to patient organisations made in 2019?

For peer review only

Stakeholder survey, Part 2. Questions to public or multistakeholder bodies overseeing pharmaceutical industry payment disclosure

Dear Sir or Madam

I would be grateful if you could answer a few questions listed below regarding the way in which your institution discloses payments made by drug companies to individuals and organisations within the healthcare system in your country. Your answers would be very helpful in informing scholarly publications on which I am currently working that are seeking to present a full and accurate picture of payments made by the pharmaceutical industry in Europe. Please let me know should you have any questions about this research.

Some of the matters covered in my questions have been touched upon on your website. However, I would be extremely grateful for your answers to avoid any misunderstandings which may be caused by language issues, particularly as they relate to complex regulatory matters.

Thank you very much in anticipation for your valuable time in answering my questions.

I look forward to hearing from you.

Sincerely yours

Piotr Ozieranski

For peer review only

I would be grateful if you could answer the following questions regarding the nature of payments disclosed by [insert name of institution]

1. What industries are covered by the disclosure requirements (e.g. pharmaceutical, medical device, veterinary)?
2. Please could you briefly describe what is meant by each type of the industry covered by the disclosure?
3. What types of payment recipients are covered by the disclosure requirements (e.g. healthcare professionals, healthcare organisations, patient organisations)? Are these definitions consistent with the ones used in the EFPIA Code of Practice (https://www.efpia.eu/media/554677/efpia-code-2020.pdf) ?
4. Please could you briefly describe what is meant by each type of recipient (e.g. what is meant by the healthcare processional or healthcare organisation)? Are these definitions consistent with the ones used in the EFPIA Code of Practice?
5. What are the categories of payments covered by the disclosure managed by your institution (e.g. sponsorship of conference attendance, consultancy fees, research and development)? Are they the same one as those used in the EFPIA Code of Practice?
6. Please could you briefly describe what is meant by each type of payment (e.g. what is meant sponsorship of by conference attendance)? Are these definitions consistent with the ones used in the EFPIA Code of Practice?
7. If the definitions of payments or recipients used by your institution are not consistent with the ones used in the EFPIA Code of Practice, please could you explain why this is the case? In other words, why alternative definitions have been created for the reporting purposes of your institution as opposed to using the ones introduced by the EFPIA Code of Practice?
8. Are all payments to all payment recipients publicly disclosed by your organisations or are any payments or recipient types exempted from public disclosure?
9. Are payments reported by your institution reported on an individual basis (i.e. each payment has a separate database entry) or are they aggregated on a yearly basis (i.e. all payments of a certain type from a certain company are reported jointly)?
10. What are the responsibilities of companies making payments in relation to their disclosure?
11. What are the responsibilities of payment recipients in relation to their disclosure (e.g. do they need to disclose payments themselves or just verify disclosures made by companies making payments)?
12. Are payment disclosures managed by your institution reported based on calendar years or financial years? If payments are made based on financial years, please could you specify what are the start and end dates of a financial year in your country?
13. Are payment values made publicly available by your institution the same as those received by payment recipients (e.g. do they include the value of any taxes, such as VAT, paid by companies making the payments)?
14. If payments reported in by your institution are not reported consistently with or without relevant taxes (e.g. VAT), where might database users find information on approaches to tax reporting taken by each company making payments.
15. Do all industries covered by the payment disclosures managed by your institution report payments in exactly the same way (e.g. using the same definitions of payments, payment categories and recipients)?
16. Is the database of payments managed by your institution downloadable or not? Please could you explain why the decision has been made to make it downloadable (or not)?
17. Please could you send me the most recent copy of the regulation and/or policy which governs the disclosure of payments made your institution (if available, I would appreciate an English version of the document)?

For peer review only

Please could you answer the following questions regarding the disclosure requirements covered by your institution and the self-regulatory disclosure system overseen by the European Federation of Pharmaceutical Industries (EFPIA), as described in its Code of Practice?

18. Do pharmaceutical companies in your country have to disclose payments with your institution as well as in line with the disclosure system managed by EFPIA (i.e. do companies need to disclose payments to healthcare professionals and organisations with your institution as well as using disclosure reports comprising payments to healthcare professionals and organisations published on their individual websites)?
19. The EFPIA code of practice covers the category of payments related to research and development. Are these payments disclosed by your institution on a named basis or on drug company websites, in line with the EFPIA code of practice?

If payments made in 2019 calendar were reported year please could you answer the following questions regarding payments from <u>all industries</u> whose payments are managed by your institution?

20. How many companies reported payments in 2019?
21. What was the overall number of healthcare professionals receiving payments in 2019?
22. (If relevant) What was the overall number of healthcare organisations receiving payments in 2019?
23. (If relevant) What was the overall number of patient organisations receiving payments in 2019?
24. What was the overall value of payments made to healthcare professionals in 2019?
25. (If relevant) What was the overall value of payments made to healthcare organisations in 2019?
26. (If relevant) What was the overall value of payments made to patient organisations in 2019?

Please could you answer the following questions regarding only payments made by <u>pharmaceutical companies only</u> in the 2019 calendar year?

27. How many pharmaceutical companies reported payments in 2019?
28. Is the number of companies which did not disclose any payments made in 2019 known? If so, please could you state this number?
29. What was the overall number of healthcare professionals receiving payments from pharmaceutical companies in 2019?
30. (If relevant) What was the overall number of healthcare organisations receiving payments receiving payments from pharmaceutical companies in 2019?
31. (If relevant) What was the overall number of patient organisations receiving payments receiving payments from pharmaceutical companies in 2019?
32. What was the overall share of healthcare professionals consenting to their payments being disclosed in 2019?
33. (If relevant) What was the overall share of healthcare organisations consenting to their payments being disclosed in 2019?
34. (If relevant) What was the overall share of patient organisations consenting to their payments being disclosed in 2019?
35. What was the overall value of all payments made to healthcare professionals by pharmaceutical companies in 2019?
36. (If relevant) What was the overall value of payments made to healthcare organisations by pharmaceutical companies in 2019?

For peer review only

37. (If relevant) What was the overall value of payments made to patient organisations by pharmaceutical companies in 2019?
38. Please could you provide the value of each category of payments received by healthcare professionals from pharmaceutical companies in 2019?
39. (If relevant) Please could you provide overall the value of each category of payments received by healthcare organisations from pharmaceutical companies in 2019?
40. (If relevant) Please could you provide overall the value of each category of payments received by patient organisations from pharmaceutical companies in 2019?

Finally, I would be grateful if you could answer a few general questions regarding the direction nature of payments disclosed.

41. The obligatory disclosure of payments by a public body, such as your institution, seem be at odds the approach taken by many other European countries, which support a self-regulatory system managed by the pharmaceutical industry and allowing payment recipients not to have their payments disclosed based on the General Data Protection Regulation (GDPR). How does the system for payment disclosure managed by your institution addresses potential concerns regarding data privacy of payment recipients?
42. What are the advantages of the mandatory disclosure of payments, overseen by your institution, over a self-regulatory payment system based on the EFPIA Code of practice, existing in many other European countries?
43. What is the source of funding of the disclosure system managed by your institution (e.g. general taxation, health insurance, company fees)?
44. What is the yearly cost of maintaining the disclosure system managed by your institution?
45. How there been any examples of healthcare professionals not complying with the requirements of the disclosure system managed by your institution? If so, how were they addressed?
46. (if relevant) How there been any examples of healthcare organisations not complying with the requirements of the disclosure system managed by your institution? If so, how were they addressed?
47. (if relevant) How there been any examples of patient organisations not complying with the requirements of the disclosure system managed by your institution? If so, how were they addressed?
48. How there been any examples of pharmaceutical companies not complying with the requirements of the disclosure system managed by your institution? If so, how were they addressed?
49. Does your institution monitor who uses the disclosed payment data? If so, would you be able to say who the key types of users are?
50. What does your institution do to encourage the use of disclosed data (e.g. public campaigns)?

For peer review only

Online Supplement 2. Responses to stakeholder survey

Part 1. Pharmaceutical industry trade groups

Country	Name of pharmaceutical industry trade group	Reply received	Nature of reply
AUSTRIA	Fachverband der Chemischen Industrie Österreichs	Yes	Answered all questions
BOSNIA AND HERZEGOVINA	Association of Research-based Medicine Producers in Bosnia & Herzegovina	No	N/A
BULGARIA	Association of the Research-based Pharmaceutical Manufacturers in Bulgaria	No	N/A
CROATIA	iF! – Inovativna farmaceutska inicijativa	No	N/A
CYPRUS	The Cyprus Association of Research and Development Pharmaceutical Companies	No	N/A
CZECH REPUBLIC	Asociace inovativního farmaceutického průmyslu	Yes	Answered some questions
DENMARK	LægemiddelindustriforeningenLersø	Yes	Answered some questions
ESTONIA	The Association of Pharmaceutical Manufacturers in Estonia	No	N/A
FINLAND	Lääketeollisuus	Yes	Answered all questions
GERMANY	Freiwillige Selbstkontrolle für die Arzneimittelindustrie	No	N/A
GREECE	Hellenic Association of Pharmaceutical Companies	No	N/A
HUNGARY	Association of Innovative Pharmaceutical Manufacturers	No	N/A
ICELAND	Icelandic Association of the Pharmaceutical Industry	Yes	Holding message
IRELAND	Irish Pharmaceutical Healthcare Association	Yes	Answered all questions
ITALY	Associazione delle imprese del farmaco	No	N/A
LATVIA	Association of International Innovative Pharmaceuticals Producers	Yes	Answered some questions
LITHUANIA	Innovative Pharmaceutical Industry Association	Yes	Answered all questions
LUXEMBOURG	Association pharmaceutique luxembourgeoise	Yes	Answered all questions
NORTH MACEDONIA	Association of Foreign Innovative Manufacturers in Macedonia	Yes	Answered all questions
NORWAY	Legemiddelindustrien	Yes	Answered all questions
POLAND	Związek Pracodawców Innowacyjnych Firm Farmaceutycznych	Yes	Holding message

For peer review only

PORTUGAL	Associação Portuguesa da Indústria Farmacêutica	No	N/A
ROMANIA	Association of International Medicines Manufacturers	No	N/A
RUSSIA	Association of International Pharmaceutical Manufacturers	No	N/A
SERBIA	Innovative Drug Manufacturers' Association	No	N/A
SLOVAKIA	Association of the Innovative Pharmaceutical Industry	No	N/A
SLOVENIA	Forum of International Research and Development Pharmaceutical Companies, EIG	No	N/A
SPAIN	Asociación Nacional Empresarial de la Industria Farmacéutica	Yes	Answered some questions
SWEDEN	Läkemedelsindustriföreningen	Yes	Answered all questions
SWITZERLAND	Science Industries Switzerland	Yes	Holding message
TURKEY	Araştırmacı İlaç Firmaları Derneği	Yes	Answered all questions
UK	Association of the British Pharmaceutical Industry	Yes	Answered all questions
UKRAINE	Association of Pharmaceutical Research and Development	No	N/A
EUROPE	EFPIA	No	N/A

For peer review only

Part 2. Public and multistakeholder bodies overseeing payment disclosure

Country	Name of public or multistakeholder body overseeing payment disclosure	Nature of authority overseeing payment disclosure	Reply received	Nature of reply
BELGIUM	betransparent.be	Multistakeholder body	No	N/A
DENMARK	Danish Medicines Agency	Public body	Yes	Answered some questions
ESTONIA	State Agency of Medicines	Public body	No	N/A
FRANCE	Ministry of Health	Public body	Yes	Holding message
GREECE	National Organisation for Medicines	Public body	No	N/A
HUNGARY	National Institute of Pharmacy and Nutrition	Public body	Yes	Answered all questions
LATVIA	Latvian Health Inspectorate	Public body	Yes	Answered all questions
LITHUANIA	Lithuanian State Medicines Control Agency	Public body	Yes	Answered all questions
THE NETHERLANDS	Vereniging Innovatieve Geneesmiddelen	Multistakeholder body	Yes	Inquiry redirected
PORTUGAL	INFARMED - National Authority of Medicines and Health Products	Public body	Yes	Answered some questions
ROMANIA	National Agency for Medicines and Medical Devices in Romania	Public body	Yes	Holding message
SLOVAKIA	National Health Information Center	Public body	Yes	Answered some questions
TURKEY	Turkish Medicines and Medical Devices Agency	Public body	Yes	Holding message

For peer review only

Online Supplement 3. Examples of deviations of data reporting formats from the EFPIA standard "disclosure template"

Part 1: EFPIA "disclosure template"

ANNEX A (binding)
Standardised disclosure template

ANNEX A - STANDARDISED DISCLOSURE TEMPLATE

Date of publication: ....

	Full Name	**HCPs:** City of Principal Practice **HCOs:** city where registered	Country of Principal Practice	Principal Practice Address	Unique country identifier *OPTIONAL*	Donations and Grants to HCOs *(Art. 3.01.1.a)*	Contribution to costs of Events *(Art. 3.01.1.b & 3.01.2.a)*			Fee for service and consultancy *(Art. 3.01.1.c & 3.01.2.c)*			**TOTAL** *OPTIONAL*
	(Art. 1.01)	*(Art. 3)*	*(Schedule 1)*	*(Art. 3)*	*(Art. 3)*		Sponsorship agreements with HCOs / third parties appointed by HCOs to manage an Event	Registration Fees	Travel & Accommodation	Fees	Related expenses agreed in the fee for service or consultancy contract, including travel & accommodation relevant to the contract		
**HCPs**	***INDIVIDUAL NAMED DISCLOSURE - one line per HCP*** *(i.e. all transfers of value during a year for an individual HCP will be summed up: itemization should be available for the individual Recipient or public authorities' consultation only, as appropriate)*												
	Dr A					N/A	N/A	*Yearly amount*	*Yearly amount*	*Yearly amount*	*Yearly amount*		
	Dr B					N/A	N/A	*Yearly amount*	*Yearly amount*	*Yearly amount*	*Yearly amount*		
	etc.					N/A	N/A	*Yearly amount*	*Yearly amount*	*Yearly amount*	*Yearly amount*		
	OTHER, NOT INCLUDED ABOVE - where information cannot be disclosed on an individual basis for legal reasons												
	**Aggregate amount attributable to transfers of value to such Recipients** - *Art. 3.02*					N/A	N/A	*Aggregate HCPs*	*Aggregate HCPs*	*Aggregate HCPs*	*Aggregate HCPs*		*Optional*
	**Number of Recipients in aggregate disclosure** - *Art. 3.02*					N/A	N/A	*number*	*number*	*number*	*number*		*Optional*
	% of the number of Recipients included in the aggreate disclosure in the total number, by category, of Recipients disclosed - *Art. 3.02*					N/A	N/A	%	%	%	%		N/A
**HCOs**	***INDIVIDUAL NAMED DISCLOSURE - one line per HCO*** *(i.e. all transfers of value during a year for an individual HCO will be summed up: itemization should be available for the individual Recipient or public authorities' consultation only, as appropriate)*												
	HCO 1					*Yearly amount*	*Yearly amount*	*Yearly amount*	*Yearly amount*	*Yearly amount*	*Yearly amount*		*Optional*
	HCO 2					*Yearly amount*	*Yearly amount*	*Yearly amount*	*Yearly amount*	*Yearly amount*	*Yearly amount*		*Optional*
	etc.					*Yearly amount*	*Yearly amount*	*Yearly amount*	*Yearly amount*	*Yearly amount*	*Yearly amount*		*Optional*
	OTHER, NOT INCLUDED ABOVE - where information cannot be disclosed on an individual basis for legal reasons												
	**Aggregate amount attributable to transfers of value to such Recipients** - *Art. 3.02*					*Aggregate HCOs*	*Aggregate HCOs*	*Aggregate HCOs*	*Aggregate HCOs*	*Aggregate HCOs*	*Aggregate HCOs*		*Optional*
	**Number of Recipients in aggregate disclosure** - *Art. 3.02*					*number*	*number*	*number*	*number*	*number*	*number*		*Optional*
	% of the number of Recipients included in the aggreate disclosure in the total number, by category, of Recipients disclosed - *Art. 3.02*					%	%	%	%	%	%		N/A

**R&D**	***AGGREGATE DISCLOSURE***		
	Transfers of Value re Research & Development as defined - *Article 3.04 and Schedule 1*	*TOTAL AMOUNT*	*OPTIONAL*

latest update: 27 June 2019

Note

The screenshot was taken from version the EFPIA Code which was in force the time of writing.[1]

Part 2: Examples of data reporting not following the EFPIA "disclosure template"

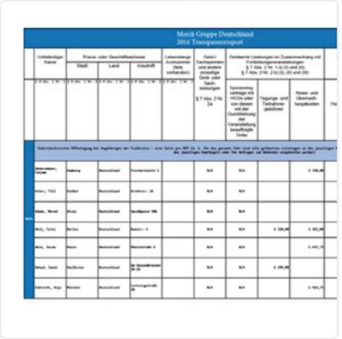

de__merck__2016.pdf
PDF

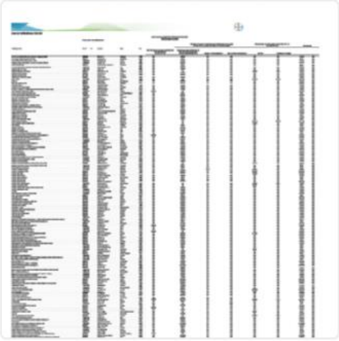

de__bayer__2016.pdf
PDF

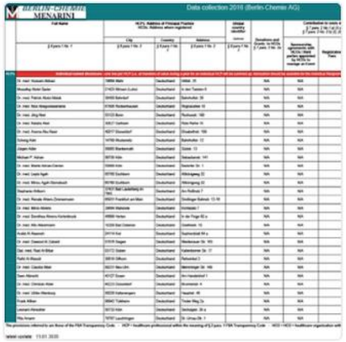

de__berlin-chemie__2016.pdf
PDF

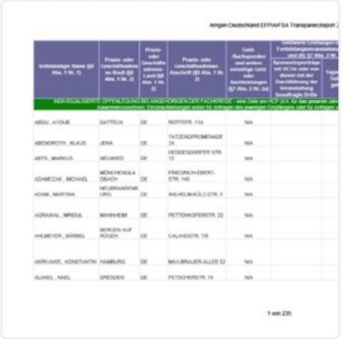

de__amgen__2016.pdf
PDF

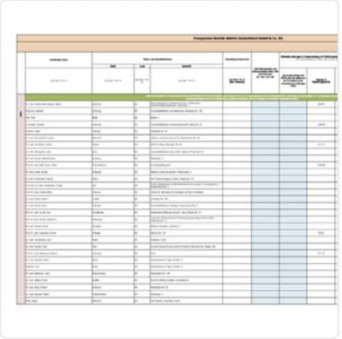

de__abbvie__2016.pdf
PDF

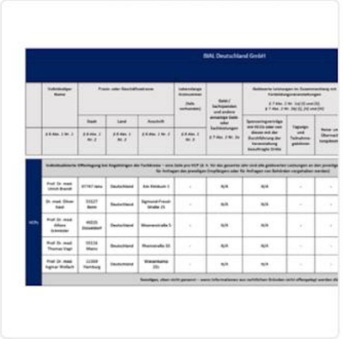

de__bial__2017.pdf
PDF

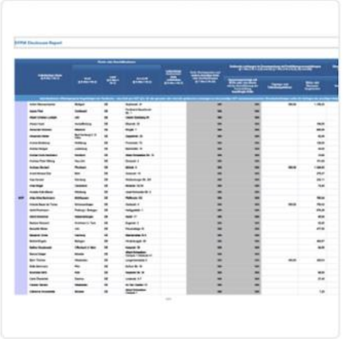

de__biogen__2017.pdf
PDF

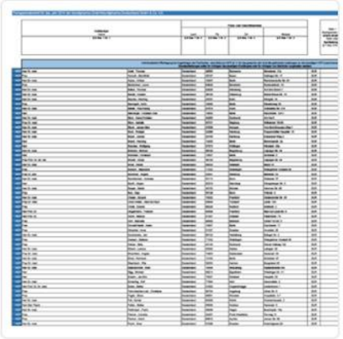

de__mundipharma__2016.pdf
PDF

Note

The screenshots were taken as part of the data curation process involved in creating the eurosfordocs.eu database.[2]

References

1. EFPIA. EFPIA Code of practice 2019 [Available from: https://www.efpia.eu/media/554677/efpia_codes_a5_v3-2021_sm.pdf.
2. Eurosfordocs.eu. Tech documentation, 2021 [Available from: eurosfordocs.eu/on-the-tech-side/tech-documentation/ accessed 4th January 2021.

For peer review only

Online Supplement 4. Approaches to data presentation decreasing the accessibility of payment disclosures

Part A: Techniques used when publishing payment data on individual company websites

1. Publish data as PDF documents (and not csv or XLS files) to make analysis difficult (example: most companies throughout Europe, except where the pharmaceutical industry trade groups created centralised databases).
2. Make the PDF document available only in an online viewer rather than as a separate file to prevent it from being downloaded for analysis (example: Menarini Switzerland).
3. Publish PDF documents consisting of images (and not text) to prevent any searches within the data (e.g. for recipient names) (example: Pfizer Sweden).
4. Reduce the resolution of image-based PDF documents to make them *almost* unreadable without constantly zooming in and out (example: Novo-Nordisk Spain).
5. Create PDF documents with repeated table headers occupying most of each page in the disclosure report. In some cases, if the content is image-based, the resulting PDF can exceed 1800 pages, and over 350 MB, which discourages users from opening or downloading it (example: Novartis Italy).
6. Require users to follow a lengthy process of accepting the “Terms of use” of the disclosed information to discourage engagement with the data (example: Pfizer Spain).

Note: Different techniques can be combined. For example, the disclosure report can be made available only in an online viewer (2), with each page published as an image (3) and in a low resolution (4) (example: Roche Italy).

Part B: Techniques used when publishing payment data in centralised databases.

1. Require users to follow a lengthy process of accepting the “terms of use” of the disclosed information to discourage engaging with the data (example: the Czech Transparentní spolupráce database).
2. Enable searching only for specific recipients, without the possibility of searching for companies or recipient categories (e.g. medical specialties) (the Czech Transparentní spolupráce database).
3. Make searches conditional on obtaining recipient ID numbers from another website (example: the Czech Transparentní spolupráce database)
4. Do not include the possibility of downloading the database as a single file to prevent analysis (Examples: the Irish Transfer of Value database – and all centralised industry platforms except Disclosure UK)

For peer review only

Online supplement 5. Eurosfordocs.eu – database summary (2017-2019)[1]

Country	Disclosure reports	Successfully extracted (parsed) disclosure reports	Parse ratio	Companies associated with parsed disclosure reports	Number of payments to healthcare professionals and organisations	Value of payments (€)[2]
UK	1[3]	1	100%	141	164,112	1,771,785,871
Germany	112	89	79%	32	103,477	1,524,231,568
Spain	60	48	80%	16	370,444	959,704,223
Italy	60	57	95%	19	143,244	954,063,974
Switzerland	138	117	85%	41	36,503	471,638,889
Sweden	184	168	91%	68	15,434	249,913,018
Ireland	1[3]	1	100%	46	18,312	97,259,959
Total	556	481		160	851,526	6,028,597,501

Notes.

[1] – All data is accurate as of January 2021. Eurosfordocs.eu is updated regularly to reflect occasional changes in disclosure reports published by drug companies.

[2] – All payment values in non-euro currencies were converted to euros based the exchange rate obtained from the CurrencyConverter [1] a Python library for exchange rates.

[3] – The UK and Ireland are the only countries reported in the table in which all drug company payments are included in a single database. In all other countries, disclosure reports are published on individual websites for each company.

References

1. CurrencyConverter. CurrencyConverter 0.14.4 2020 [Available from: https://pypi.org/project/CurrencyConverter/ accessed 19th January 2021.

For peer review only
